# Characters evolution of *Encyclia* (Laeliinae-Orchidaceae) reveals a complex pattern not phylogenetically determined: insights from macro- and micromorphology

**DOI:** 10.1186/s12870-023-04664-3

**Published:** 2023-12-20

**Authors:** Monika M. Lipińska, Natalia Olędrzyńska, Magdalena Dudek, Aleksandra M. Naczk, Dorota Łuszczek, Peter Szabó, Manfred Speckmaier, Dariusz L. Szlachetko

**Affiliations:** 1 Department of Plant Taxonomy and Nature Conservation, Faculty of Biology, University of Gdańsk, Wita Stwosza 59, Gdańsk, 80308 Poland; 2 Foundation Polish Orchid Association, Sopot, 81825 Poland; 3 Department of Evolutionary Genetics and Biosystematics, Faculty of Biology, University of Gdańsk, Wita Stwosza 59, Gdańsk, 80308 Poland; 4 Laboratory of Electron Microscopy, Faculty of Biology, University of Gdańsk, Wita Stwosza 59, Gdańsk, 80308 Poland; 5 Individual Researcher, Vasvár, 9800 Hungary; 6 Botanischer Garten, Universität Wien, Rennweg 14/2, Raum G-10, Vienna, 1030 Austria

**Keywords:** Anatomy, Epidermis, Laeliinae, Morphology, Neotropics, Orchids, Taxonomy

## Abstract

*Encyclia* is the second-largest genus in the neotropical subtribe Laeliinae (Orchidaceae) and has more than 150 species, which are characterized by fairly consistent flower morphology. Its taxonomy and species boundaries, however, seem to be still under debate. In the present study, we first examined the lip micromorphology of 61 species of *Encyclia *sensu stricto. We correlated our results with external flower morphology and phylogenetic analyses performed on a combined dataset that included both nuclear (ITS, *Xdh*, *Phy*C) and plastid markers (*ycf*1, *rpl*32, and *trn*L-*trn*F). Phylogenetic reconstruction showed that *Encyclia *sensu stricto species form a coherent, monophyletic group. However, it is difficult to determine the relationships between the different groups within one larger clade. The groups all form distinct lineages that evolved from a common ancestor. The UPGMA cluster analysis for the seven qualitative micromorphological features clearly divides the genus into two main groups, the larger of which is further subdivided into two subgroups. None of these, however, overlap with any of the phylogeographic units distinguished in previously published papers or in presented article. It is worth noting that the groups resulting from the UPGMA analysis cannot be defined by macromorphological features. The pattern of similarities between species, taking into account both macro- and micromorphological features, is eminently mosaic in nature, and only a multifaceted approach can explain this enigmatic group.

**Supplementary Information:**

The online version contains supplementary material available at 10.1186/s12870-023-04664-3.

## Introduction

With more than 150 species [1], *Encyclia* Hook. is the second largest genus of the Neotropical subtribe Laeliinae Benth. (Orchidaceae Juss.), although several more taxa have been described in the last decades and others are still waiting for the formal description. The genus representatives are distributed from Florida and Mexico to southern Brazil [1, 2] and stand out for occupying habitats extremes when compared to other genera of the subtribe, mainly in relation to exposure to sun and drought [1].

Genus *Encyclia* was established in 1828 by Hooker [3] who based its description on what he described as *Encyclia viridiflora* Hook. Lindley (in 1853) [4] did not accept *Encyclia* as a taxon distinct from *Epidendrum* and transferred *E. viridiflora* to *Epidendrum* and included it with other similar species in the *Epidendrum* subgen. *Encyclium* Lindl. He based his decision on the presence of four pollinia and the lip being partially fused to the column (which is not, in fact, the case). Later, in 1881, Bentham [5] subdivided the section *Encyclium* into three series: *Dinema*, *Prosthechea*, and *Encyclia*. Until Schlechter’s revision in 1914 [6], the name *Encyclia* has not been applied. Since then, the taxonomy of *Encyclia* has been under ongoing discussion and many species have been moved into and out of the genus by various taxonomists who defended a broad concept of *Epidendrum* by including these species as a section (e.g. [7]). In this way, even in the 20th century, several *Encyclia* species were described as members of *Epidendrum* and were only later transferred to *Encyclia* [8]. Porto & Brade (in 1935) [9] and Hoehne (in 1952) [10] followed Schlechter’s concept and proposed combinations for most *Encyclia* species originally described in *Epidendrum*. Lemee (in 1955) [11] transferred five taxa from *Epidendrum* subgenus *Aulizeum* Lindl. to *Encyclia* and by this, enlarged the circumscription of *Encyclia* proposed by Schlechter. In 1960, Brieger [12] proposed the transfer of several taxa into the genus *Hormidium* Dressler & Pollard. One year later, Dressler [13] redefined *Encyclia* and expanded Bentham’s concept by describing two sections, *Encyclia* sect. *Encyclia* and *Encyclia* sect. *Osmophytum* Dressler. Subsequently, Dressler and Pollard (in 1971) [14] revised the genus and divided it into six sections and three subgenera. They kept *Encyclia* subgen. *Osmophytum* Dressler & Pollard, subdivided it into three sections: *Osmophytum*, *Hormidium*, and *Euchile* Dressler & Pollard. In *Encyclia* subgen. *Encyclia* they recognized four sections: sect. *Encyclia*, sect. *Brachycolumna* Dressler & Pollard, sect. *Leptophyllum* Dressler & Pollard, and sect. *Dinema* Dressler & Pollard. Later, in 1974, the same authors [15] raised the sect. *Dinema* to the subgeneric level (*Encyclia* subgen. *Dinema* Dressler & Pollard), with the single species *E. polybulbon* Dressler, and kept the remaining species in the subgenera and sections as they were previously circumscribed. Right after Dressler assembled the genus, other taxonomists began to disassemble it. Pabst et al. (in 1981) [16] refined Brieger’s concepts and moved additional taxa. They have raised *Encyclia* section *Hormidium* Dressler to the rank of genus and transferred part of the taxa of *Encyclia* section *Osmophytum* to *Anacheilium* Rchb. *ex* Hoffmanns. Many taxa treated under the *Encyclia* subgen. *Osmophytum* (including sects. *Hormidium* and *Euchile*) were later recognized by Higgins (in 1997) [17] as belonging to *Prosthechea* Knowles & Westcott. In 2001 Higgins [18] proposed *Oestlundia* as a new genus and validated *Microepidendrum* Brieger *ex* Higgins, neither of them strictly related to *Encyclia *sensu stricto. Subsequently, in 2003, Higgins et al. [2] used nuclear and plastid DNA sequence data (nrITS, plastid *mat*K and *trn*L*-trn*F) to estimate the phylogeny of *Encyclia *sensu Dressler and found that *Encyclia *sensu lato was polyphyletic. To maintain the criterion of monophyly, the genus *Encyclia *sensu Dressler has been divided into six genera: *Encyclia*, *Euchile*, *Dinema*, *Oestlundia*, *Prosthechea*, and *Microepidendrum*. Later studies revealed that *Euchile* and *Hormidium* should be considered synonyms of *Prosthechea*.

*Encyclia s.s.* (sensu Higgins et al. [2]) can be characterized by a lip bearing a cymbiform callus and a variable apex and column with two lateral wings (= staminodes) and an elongate filament giving the gynostemium an appearance of the three-toothed apex. Recent phylogenetic analysis of the genus utilizing nuclear and plastid DNA sequences [19–21] revealed that *Encyclia* is composed of lineages that are strongly correlated geographically, with some clades fully restricted to particular biogeographical areas of Neotropics. These clades are confined to such areas as Megamexico II (e.g., the *E. adenocarpos* clade), the extra-Megamexican portion of the Central American Isthmus (Costa Rica and Panama; e.g., the *E. mooreana* alliance), the West Indies (e.g., the *E. plicata* alliance), northern South America, the Andean foothills, the Guiana Shield, the Amazon Basin, or several areas of Brazil (e.g., the *E. argentinensis* alliance) such as the Mata Atlantica, the Cerrado, or the Caatinga ([21] and references therein). Only a few lineages of *Encyclia* (e.g., the *Encyclia ceratistes* species complex), and only a handful of species (e.g., *Encyclia cordigera* (Kunth) Dressler) occur in two or more of these major areas [21].

In general, *Encyclia *sensu Schlechter [6] has a uniform vegetative habit [19]. The plants are usually caespitose and have short internodes that connect ovoid to pyriform pseudobulbs. Each pseudobulb bears one to four articulate fleshy leaves and a single terminal paniculate inflorescence [19]. In contrast, the flowers are variable among species, within species, and within populations [22, 23]. Pupulin and Bogarin [23] have described the variation between years for the same individual. Some researchers correlate this floral diversity with pollination by food deception and state that it is mainly mediated by different sizes of bees [24, 25]. Flowers are usually resupinated, with a trilobed lip adnate basally to the column, but not connected with it. These features combination may be useful in distinguishing *Encyclia* from *Prosthechea* [19]. The other floral character separating both genera are the texture of segments, which are relatively thin in the former, and thick and rigid in the latter. There is growing evidence of the role played by interspecific natural hybridization within the genus, which has likely been a diversity-increasing factor during the evolution of *Encyclia* ([21] and references therein).

Until now, there is practically no data on the micromorphological features of *Encyclia* representatives. Only several papers dealing with vegetative organs have been published so far. In 2003, Pires et al. [26] investigated the taxonomic separation of the genera *Prosthechea* and *Encyclia* using leaf and root anatomical features. Recently, dos Santos et al. [27] aimed to describe the morphoanatomy of the vegetative system of three *Encyclia* species: *E. chapadensis* L.C. Menezes, *E. linearifolioides* (Kraenzl.) Hoehne, and *E. osmantha* (Barb. Rodr.) Schltr. On the other hand, floral micromorphology in *Encyclia* seems to be completely neglected, despite its undoubted importance in the pollination process. In the presented work we have investigated lip micromorphology of 61 species from *Encyclia *sensu stricto and aimed to correlate our results with the external flower morphology, as well as with phylogenetic analyses performed for the datasets including taxa of *Encyclia *sensu lato.

## Results

### Phylogenetic analysis based on combined matrix

**Fig. 1:**
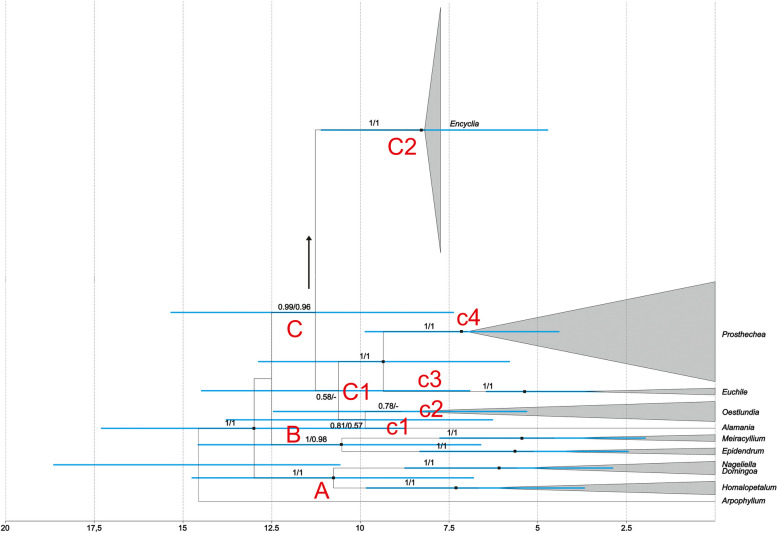
The maximum clade credibility tree for *Encyclia *sensu stricto and related genera, obtained for a combined dataset (nuclear: ITS, *Xdh*, *Phy*C and plastid markers: *ycf*1*, rpl*32*,* and *trn*L*-trn*F) using Bayesian inference. Numbers above branches indicate posterior probability and bootstrap support values from maximum likelihood analysis (PP/BS). The scale at the base of the tree indicates divergence times in millions of years ago (Mya), estimated by uncorrelated relaxed molecular clock analysis using the Yule model of speciation. Calibration points follow Givnish et al. [28]. The major clades were indicated by capital letters (A-C) or capital letters and numbers (C1, C2). The smaller subclades were indicated by lowercase letters and numbers

Species analysed were grouped into 3 main strongly supported clades, denoted A-C. The first, clade A (PP = 1, BS = 100), includes representatives of *Homalopetalum* Rolfe, *Nageliella* L.O. Williams and *Domingoa* Schltr*.*, while clade B (PP = 1, BS = 98) includes species of *Meiracyllium* Rchb. f. and some representatives of *Epidendrum* L. Most of the analysed species of the clade A are restricted to, or have their center of distribution in, Mesoamerica and the Antilles, but can also be found in the northern part of South America. Clade C also has strong support at the node (PP = 0.99, BS = 0.96) and is divided into two evolutionary lineages (marked as C1 and C2). However, within subclade C1, which includes taxa from *Alamania* Lex. (group c1), *Oestlundia* W.E. Higgins (group c2), *Euchile* (Dressler & G.E. Pollard) Withner (group c3) and *Prosthechea *sensu lato (group c4), the relationships between particular groups are not resolved due to polytomy and lack of support at the node of subclade C1 (PP = 0.58, BS less than 50). Certainly, the representatives of these genera are related and closely related to *Encyclia *sensu stricto (clade C2, Fig. 1). However, on the basis of the results obtained, it is not possible to determine the relationship between them.

**Fig. 2:**
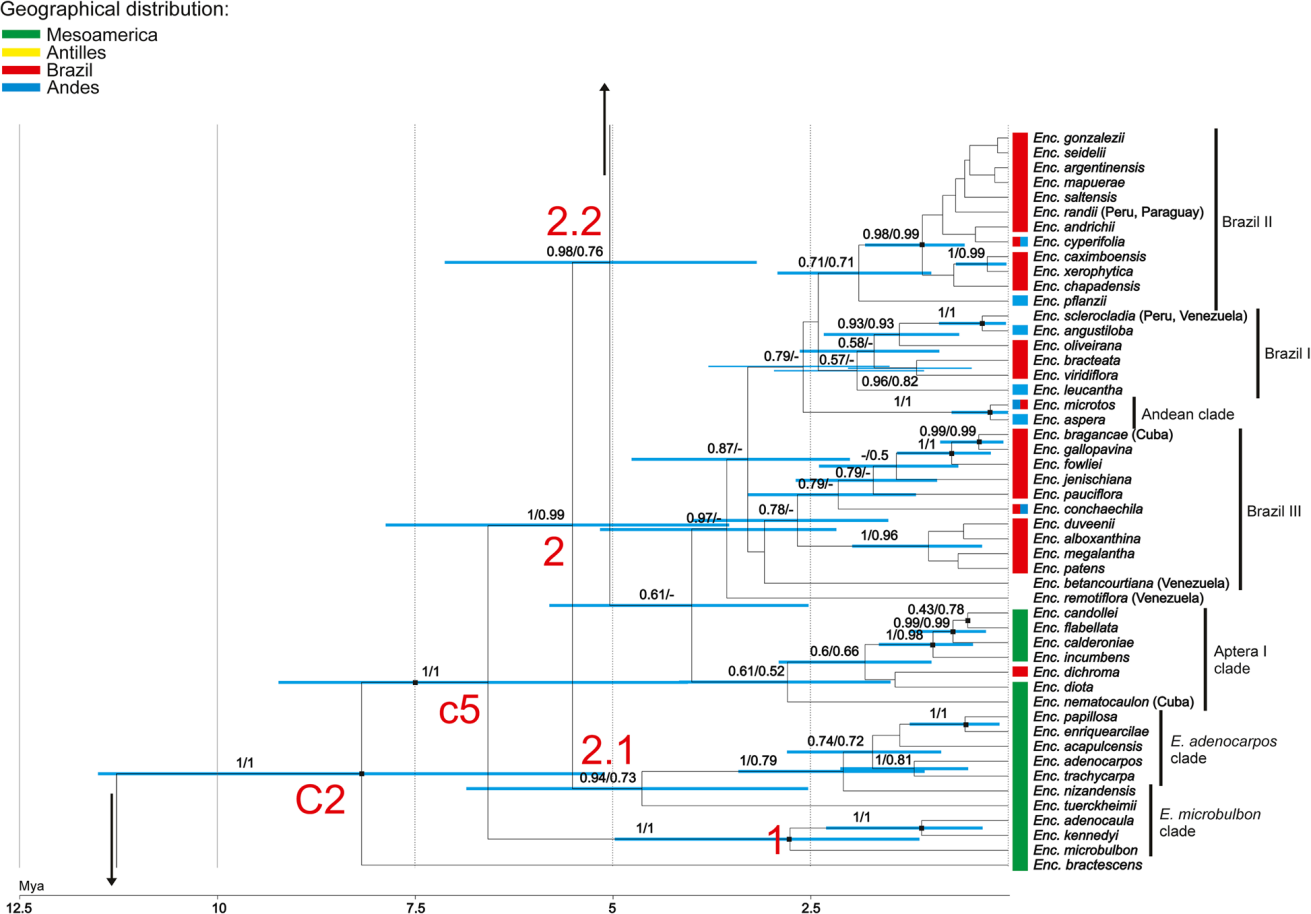
The first part of the maximum clade credibility tree presenting relationships within clade C2 for representatives of *Encyclia *sensu stricto, obtained for a combined dataset (nuclear: ITS, *Xdh*, *Phy*C and plastid markers: *ycf*1*, rpl*32*,* and *trn*L*-trn*F) using Bayesian inference. Numbers above branches indicate values of posterior probability and bootstrap support of maximum likelihood analysis (PP/BS). Rectangles next to taxon names indicate geographic distribution. Discussed subclades are indicated by lowercase letters and numbers. The scale at the base of the tree indicates divergence times in millions of years ago (Mya), as estimated by uncorrelated relaxed molecular clock analysis using the Yule model of speciation

**Fig. 3:**
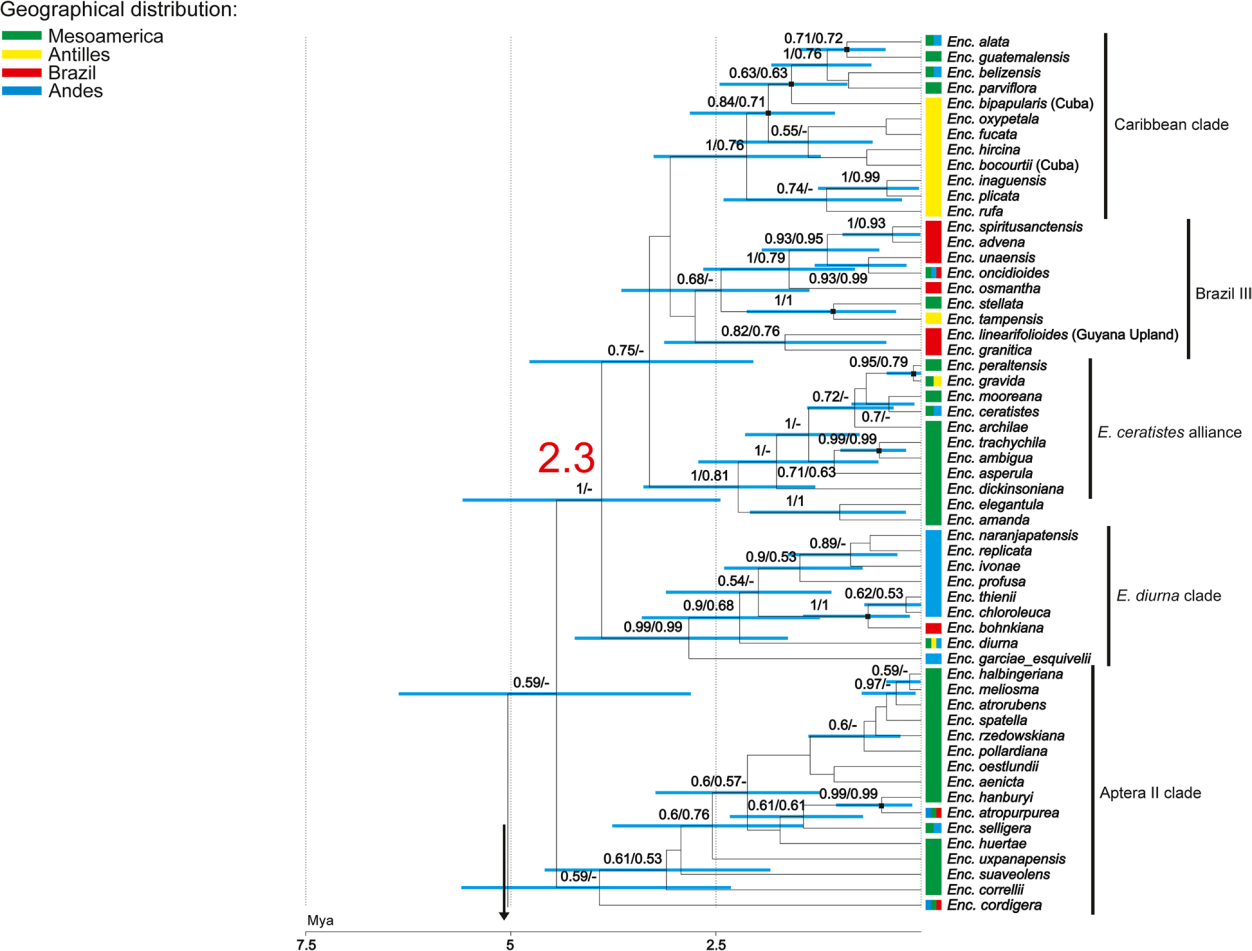
The second part of the maximum clade credibility tree presenting relationships within clade C2 for representatives of *Encyclia *sensu stricto, obtained for a combined dataset (nuclear: ITS, *Xdh*, *Phy*C and plastid markers: *ycf*1*, rpl*32*,* and *trn*L*-trn*F) using Bayesian inference. The numbers above branches indicate values of posterior probability and bootstrap support of maximum likelihood analysis (PP/BS). Rectangles next to taxon names indicate geographic distribution. Discussed subclades are indicated by lowercase letters and numbers. The scale at the base of the tree indicates divergence times in millions of years ago (Mya), as estimated by uncorrelated relaxed molecular clock analysis using the Yule model of speciation

The species of *Encyclia *sensu stricto formed a coherent, monophyletic group (clade C2, PP = 1, BS = 100, Fig. 1), in which the basal taxa is *E*. *bractescens* (Lindl.) Hoehne (Fig. 2). The remaining species of *Encyclia* were grouped in subclade c5 (Figs. 2 and 3). It is further subdivided into two more, sister to each other, groups marked as 1 and 2 (Figs. 2 and 3). The first one is much smaller and contains only 3 species: *E*. *microbulbon* (Hook.) Schltr*.*, *E*. *adenocaula* (Lex.) Schltr*.*, and *E*. *kennedyi* (Fowlie & Withner) Hágsater. On the other hand, within group 2, we can observe that the remaining species of *Encyclia* have divided into two more (2.1 and 2.2), but with average support (PP = 0.94, BS = 73; PP = 0.98, BS = 76, Fig. 2). Within group 2.1 (Fig. 2) the internal clades have a strong support at the nodes, unlike group 2.2 (Figs. 2 and 3) where we did not get high values PP and BS for most of the smaller clades. Therefore, we again have a situation where we cannot determine the relationship between the individual groups within a large clade. It may be that the variability within the DNA sequences of these taxa is not sufficient.

### Divergence times estimates

Dating analysis suggests (Fig. 1), that representatives of the Laeliinae are a relatively young group. We found that their divergence time estimates ranged from 10.5 to 18.5 Mya (million years ago). While the most common ancestor of this subtribe evolved about 14.5 Mya. The ancestor that gave rise to an evolutionary line that includes the present-day representatives of the *Encyclia *sensu stricto appeared about 11.5 Mya (clade C, Fig. 1). Two major divergence events occurred within this lineage at approximately 10.5 (clade C1) and 8 Mya (clade C2). The conclusion is that the immediate ancestor of the *Encyclia* appeared between 8.5 Mya and 8 Mya (Fig. 2).

### Ancestral state reconstruction of micro- and macromorphological features

**Fig. 4:**
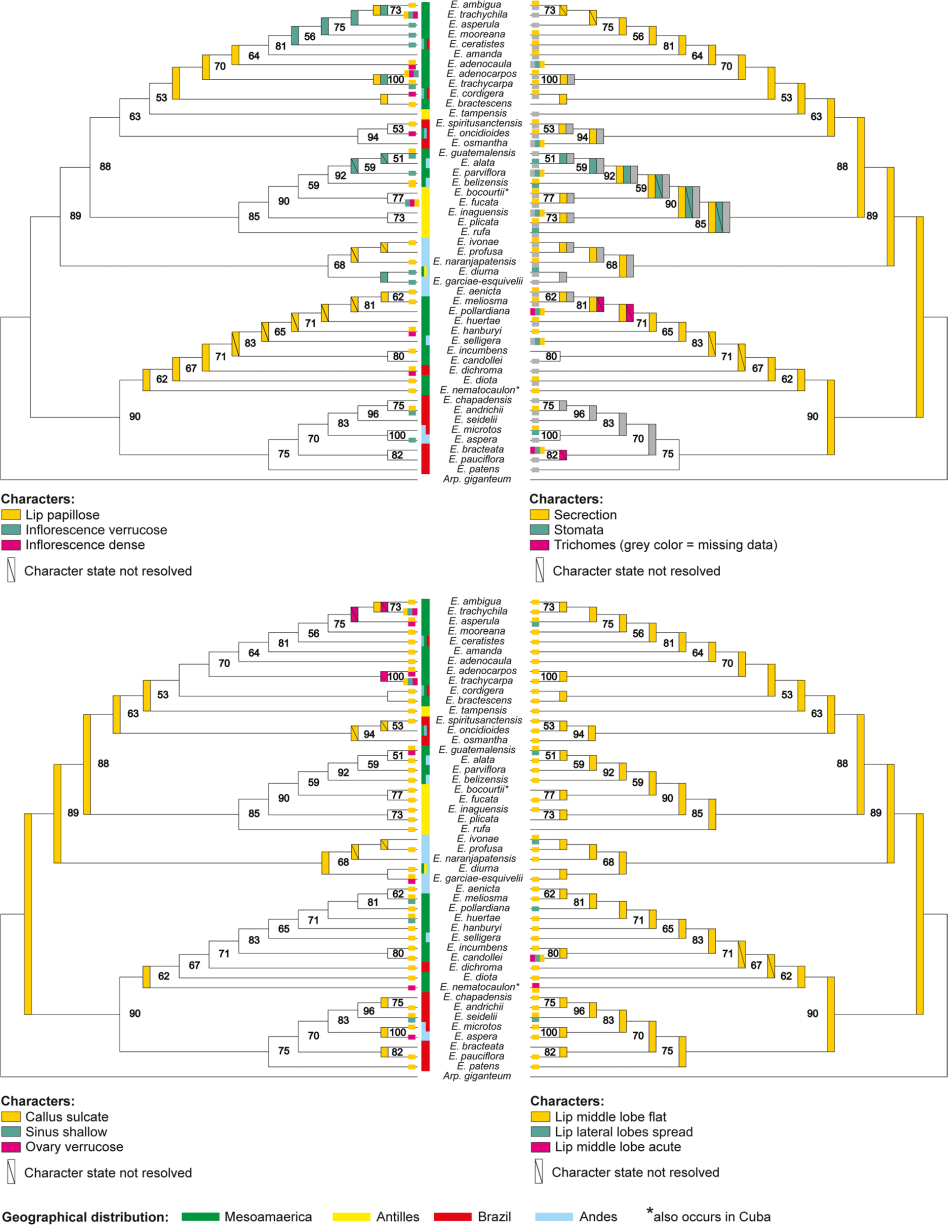
Consensus trees from ML analysis of 48 *Encyclia* species with results of ancestral state reconstruction of micro- and macromorphological features. Numbers at nodes indicates the bootstrap support from ML analysis

The ancestral states were reconstructed for 12 morphological features. Results are presented at four phylogenetic trees (Fig. 4), with bootstrap support values given at the nodes. As both, Bayesian and ML analyses, produced comparable topologies, we decided to present our results on the tree obtained from ML reconstruction, which seems to be little bit better resolved. Despite the high level of polytomy (we considered BS values higher than 75 as reliable ones), we were able to distinguish 2–4 big, well supported clades within *Encyclia*. Our results suggest that most of the studied morphological features arose rather independently within different, separated lineages. The presence of stomata, dense, verrucose inflorescence, verrucose ovary, papillose lip with spread lateral lobes and presence of shallow sinus evolved at least few times in the evolution course and are presented within all recognized, well supported clades. The last common ancestor of all *Encyclia* species were characterized with sulcate callus, flat lip middle lobe and presence of secretion. However, all these features were lost in the course of evolution in some offspring lineages. Finally, only two species (*E. candollei* (Lindl.) Schltr. and *E. nematocaulon* (A. Rich.) Acuña), possess lip with acute middle lobe. This feature was not present in its last common ancestor.

### Micromorphological analysis

Detailed results of the micromorphological study are presented in Table S1 (Additional file 1). A common feature for all species examined was striate cuticule. The lip surface of 25 species was more or less papillose. The lateral lobes of 23 species were covered in different papillae. In 11 cases they were conical in shape (*E. adenocarpa* (Lex.) Schltr., *E. adenocaula* (Lex.) Schltr*., E. cordigera, E. dichroma* (Lindl.) Schltr*., E. diota* (Lindl.) Schltr*., E. hanburyi* (Lindl.) Schltr*., E. meliosma* (Rchb. f.) Schltr*., E. papillosa* (Bateman) Ag.-Olav*., E. spiritusanctensis* L.C. Menezes*, E. trachycarpa* (Lindl.) Schltr*.,* and *E. trachychila* (Lindl.) Schltr*.*), in nine obpyriform (*E. acutifolia* Schltr.*, E. altissima* Schltr.*, E. ambigua* (Lindl.) Schltr*., E. diurna* (Jacq.) Schltr., *E. fucata* (Lindl.) Schltr*., E. moebusii* H.A. Dietr*., E. naranjapatensis* Dodson*, E. oblongata* (A. Rich.) Acuña, and *E. parviflora* (Regel) Withner), finally in three cases they were mixed: mostly conical with some obpyriform (*E. incumbens* (Lindl.) Mabb*.* and *E. virens* Schltr*.*) or mostly obpyriform with some conical (*E. belizensis* (Rchb. f.) Schltr*.*). Callus of 33 species was classified as glabrous. What should be noted, this part of the lip seemed to possess the most variable set of papillae shapes. Eight species had conical papillae (*E. adenocarpa, E. adenocaula, E. dichroma, E. fucata, E. huertae* Soto Arenas & R. Jiménez*, E. papillosa, E. selligera* (Bateman *ex* Lindl.) Schltr.*,* and *E. trachycarpa*), one – conical and obpyriform (*E. andrichii* L.C. Menezes), eight – conical and villiform (*E. amanda* (Ames) Dressler*, E. candollei, E. cordigera, E. hanburyi, E. incumbens, E. mooreana* (Rolfe) Schltr*., E. pollardiana* (Withner) Dressler & G.E. Pollard*,* and *E. trachychila*), three – obpyriform (*E. ambigua, E. belizensis, E. garciae-esquivelii* Carnevali & I. Ramírez), one – obpyriform and villiform (*E. bractescens*), and finally seven – villliform papillae (*E. aenicta* Dressler & G.E. Pollard*, E. bracteate* Schltr. *ex* Hoehne*, E. diota, E. meliosma, E. naranjapatensis, E. nematocaulon,* and *E. pauciflora* (Barb. Rodr.) Porto). In majority of species, namely 41, had the middle lobe glabrous. Three species had conical papillae (*E. adenocaula, E. hanburyi,* and *E. spiritusanctensis*), six – conical and obpyriform (*E. ambigua, E. cordigera, E. incumbens, E. nematocaulon, E. papillosa*, and *E. trachycarpa*), two – conical and villiform (*E. adenocarpa* and *E. dichroma*), and finally nine species had obpyriform papillae (*E. acutifolia, E. altissima, E. belizensis, E. bractescens, E. fucata, E. ivonae* Carnevali & G.A. Romero*, E. phoenicea* (Hook.) Schltr*., E. seidelii* Pabst, and *E. virens*). In general, lip surface of eight species possessed different types of trichomes (Fig. S1 in Additional file 1). These were: clearly single-celled (*E. bractescens, E. hanburyi, E. microtos* and *E. nematocaulon*), one to two-celled (*E. ambigua*), two-celled (*E. incumbens*), and multicellular (*E. bracteata, E. pollardiana*). In nine species the presences of stomata was noted on the lip surface (Fig. S2, S3 in Additional file 1), and these were *E. adenocaula, E. alata* (Bateman) Schltr*., E. altissima, E. belizensis, E. bracteata, E. diurna, E. inaguensis* Nash *ex* Britton & Millsp*., E. microtos* (Rchb. f.) Hoehne, and *E. rufa* (Lindl.) Britton & Millsp. In as many as 38 species residues of some kind of secretion were visible (Figs S4-S7 in Additional file 1). Lastly, on the lip surface of eight species (*E. acutifolia, E. adenocaula, E. altissima, E. microtes, E. odoratissima* (Lindl.) Schltr*., E. osmantha, E. profuse* (Rolfe) Dressler & G.E. Pollard and *E. virens*) some cristal-like were present (Fig. S8 in Additional file 1).

### Micro- and macromorphological variation

**Fig. 5:**
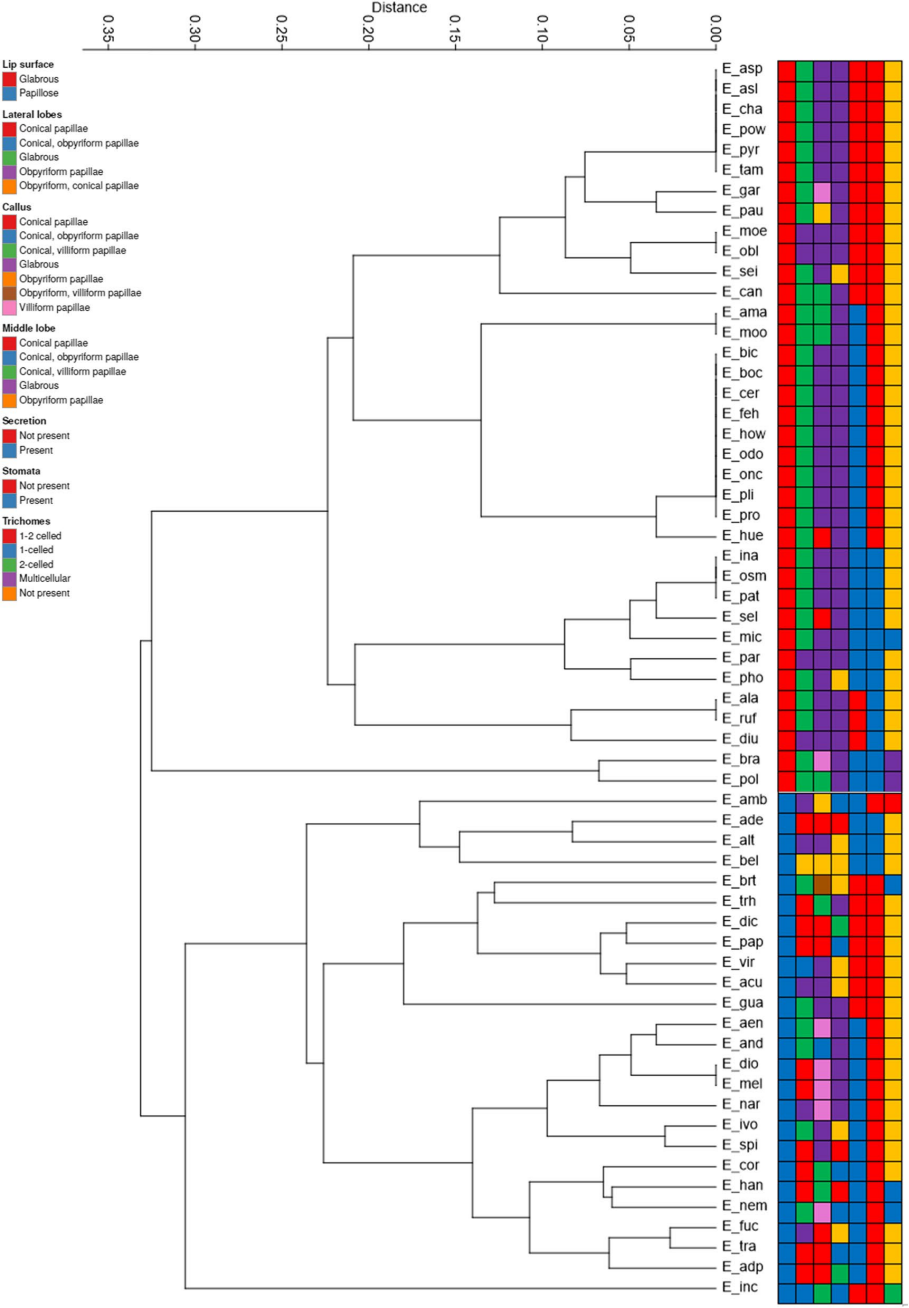
UPGMA cluster analysis of *Encyclia *sensu stricto based on the Gower’s general coefficient for seven qualitative micromorphological characters (according to Table S3 in Additional file 2)

Of the morphological features studied, the clearest division was established based on micromorphological characters. This UPGMA analysis revealed two main groups that differed in terms of lip surface (Fig. 5). The first cluster included species from *E. aspera* to *E. pollardiana*. Species belonging here are characterized by glabrous lip surface together with a glabrous callus, and the lateral and middle lobes are also mostly glabrous. The presence or absence of secretions, or stomata, are in turn the features responsible for further subdivisions into subgroups within this cluster. This group appears to be consistent, with a few exceptions (such as *E. garciae-esquivelii*, *E. pauciflora*, *E. seidelii*, *E. huertae*, *E. selligera*, *E. phoenicea* and *E. bracteata*), in contrast to the second cluster below. This group includes species from *E. ambigua* to *E. incumbens* and is more diverse than the group above in terms of most micromorphological characters. Species in this group have a papillose lip surface, but the lateral and middle lobes, as well as the callus, are varied. In turn, SIMPER analysis indicated that the appearance of callus on the lip was primarily responsible for the differences among *Encyclia* species (Table 1). However, the overall average dissimilarity equalled 72.24% for the micromorphological features used collectively in our study.

**Table 1:** SIMPER analysis results showing the contribution of traits to the discrimination of *Encyclia* species

**Micromorphology (average dissimilarity = 72.24%)**				**Combined analysis (average dissimilarity = 15.39%)**			
**Character**	**Av. dissim**	**Contrib. %**	**Cumulat. %**	**Character**	**Av. dissim**	**Contrib. %**	**Cumulat. %**
Callus	21.35	29.55	29.55	LIP4	1.44	9.35	9.35
Middle lobe	12.43	17.21	46.76	LIP7	1.35	8.78	18.13
Lateral lobes	11.29	15.62	62.39	PL4	1.17	7.57	25.70
Secretion	10.23	14.16	76.55	DS4	1.16	7.51	33.21
Stomata	6.62	9.16	85.71	LS4	1.14	7.39	40.60
Lip surface	6.27	8.68	94.39	LIP6	0.98	6.34	46.94
Trichomes	4.06	5.61	100	PL2	0.80	5.19	52.13
Cuticle	0	0	100	GYN3	0.73	4.77	56.91
**Floral characters (average dissimilarity = 14.49%)**				LIP1	0.69	4.46	61.36
LIP4	1.59	10.98	10.98	Callus	0.56	3.64	65.00
LIP7	1.54	10.59	21.57	DS2	0.53	3.46	68.45
DS4	1.24	8.58	30.15	LS2	0.50	3.26	71.72
LS4	1.24	8.54	38.69	LIP5	0.48	3.11	74.82
PL4	1.19	8.21	46.90	GYN1	0.39	2.51	77.34
LIP6	1.09	7.55	54.44	Lateral lobes	0.33	2.12	79.46
PL2	0.85	5.89	60.33	LIP3	0.30	1.97	81.42
GYN3	0.80	5.55	65.88	Middle lobe	0.23	1.51	82.93
LIP1	0.79	5.48	71.35	PL1	0.21	1.35	84.28
DS2	0.59	4.10	75.46	LIP2	0.21	1.34	85.62
LS2	0.56	3.83	79.29	DS3	0.21	1.33	86.96
LIP5	0.51	3.51	82.80	Trichomes	0.21	1.33	88.29
GYN1	0.42	2.93	85.72	LS3	0.20	1.28	89.57
LIP3	0.32	2.24	87.97	GYN2	0.20	1.27	90.84
PL1	0.26	1.81	89.77	DS1	0.17	1.13	91.96
DS1	0.24	1.63	91.40	LS1	0.16	1.06	93.02
LS1	0.22	1.55	92.95	Secretion	0.16	1.02	94.04
LS3	0.22	1.51	94.46	Stomata	0.15	0.94	94.98
GYN2	0.22	1.51	95.97	Inflorescence verrucose^	0.14	0.91	95.89
DS3	0.21	1.45	97.42	PL3	0.14	0.91	96.80
LIP2	0.21	1.43	98.85	Lml flat^	0.14	0.90	97.70
PL3	0.17	1.15	100	Lip surface	0.12	0.79	98.49
				Lll spread (up)^	0.10	0.62	99.11
				Ovary verrucose^	0.06	0.37	99.48
				Lml acute^	0.05	0.36	99.84
				Inlorescence dense^	0.02	0.16	100

For detailed description of floral traits, see the following Table 2. In turn, macromorphological (external) traits marked by a caret are described in Table S4 in Additional file 2

*Av. dissim.* Average dissimilarity, *Contrib. %* Percentage of dissimilarity explained by individual traits, *Cumulat. %* Cumulative percentage of Bray–Curtis similarity

**Table 2:** Measured floral characters used in the multivariate analyses. A detailed graphical presentation of the measured floral characters can be found in Fig. S11 in Additional file 2

Code	Floral characters
**Dorsal sepals, lateral sepals, petals, respectively**	
DS1, LS1, PL1	apex (the narrowest part of the tepal)
DS2, LS2, PL2	middle part (the widest part of the tepal)
DS3, LS3, PL3	basal part (median width of the tepal)
DS4, LS4, PL4	length of the tepal
**Lip**	
LIP1	lip base
LIP2	base of the callus
LIP3	the narrowest part of the midlobe
LIP4	the widest part of the midlobe
LIP5	the widest part of the side lobes
LIP6	length of the sidelobe
LIP7	length of the lip
**Gynostemium**	
GYN1	the widest part of the column (apical part)
GYN2	column base
GYN3	length of the column

No clear separation of *Encyclia* species was observed in the subsequent cluster analysis, based on macromorphological features (Fig. S9 in Additional file 2), or in the combined analysis, in which micro- and macromorphological traits were included in the matrix (Fig. S10 Additional file 2). In the latter, two main clusters were distinguished, while the second cluster can be divided into three smaller subgroups. Also in this case, the influence of micromorphological features on the observed grouping of *Encyclia* species is more significant than that of macromorphological features.

**Fig. 6:**
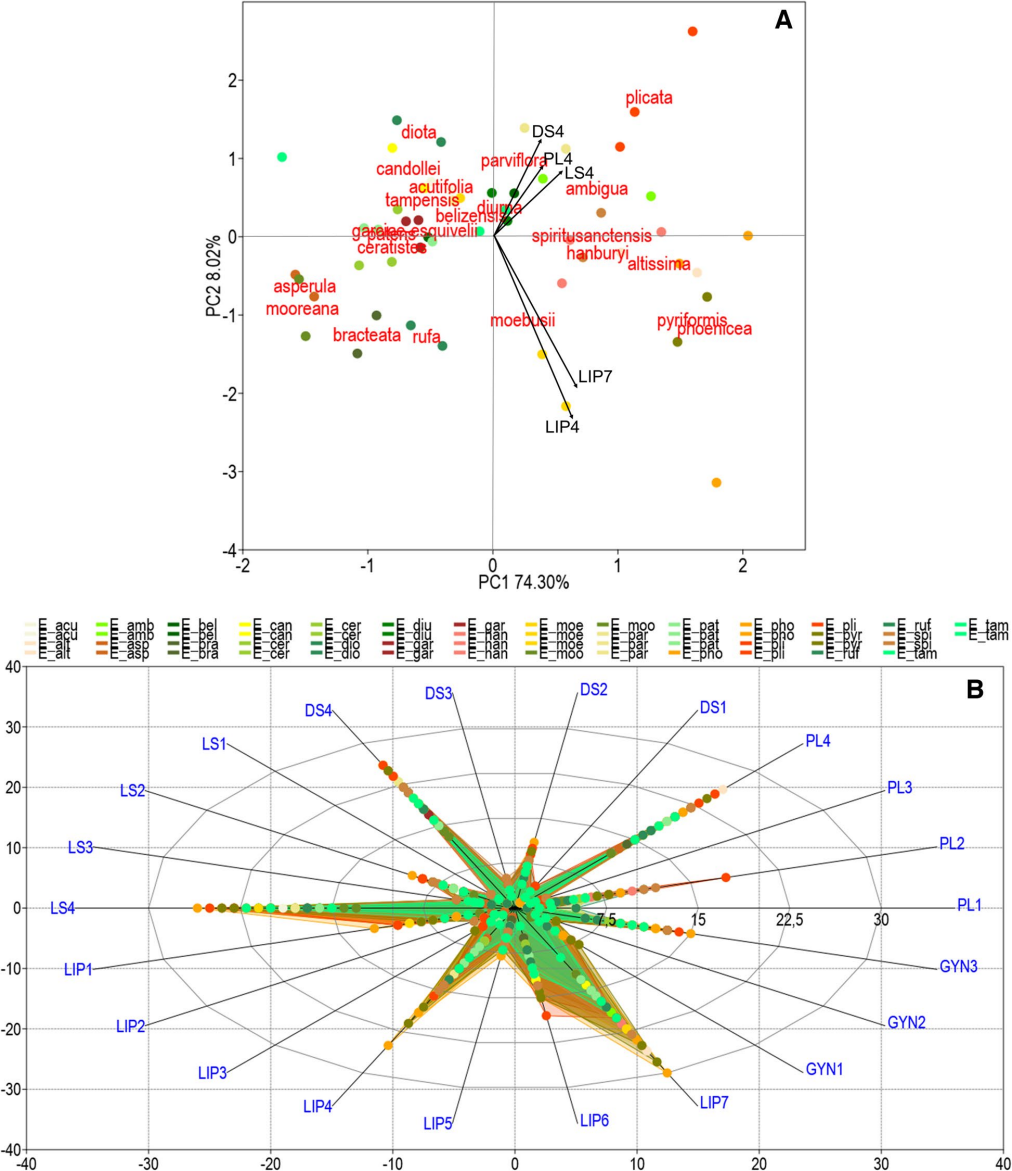
Principal component analysis (PCA) (**A**) and radar chart (**B**) of *Encyclia *sensu stricto based on floral characters only. Variables with the greatest contributions are shown as vectors. Codes for the studied species are given in Table S5 in Additional file 2

**Fig. 7:**
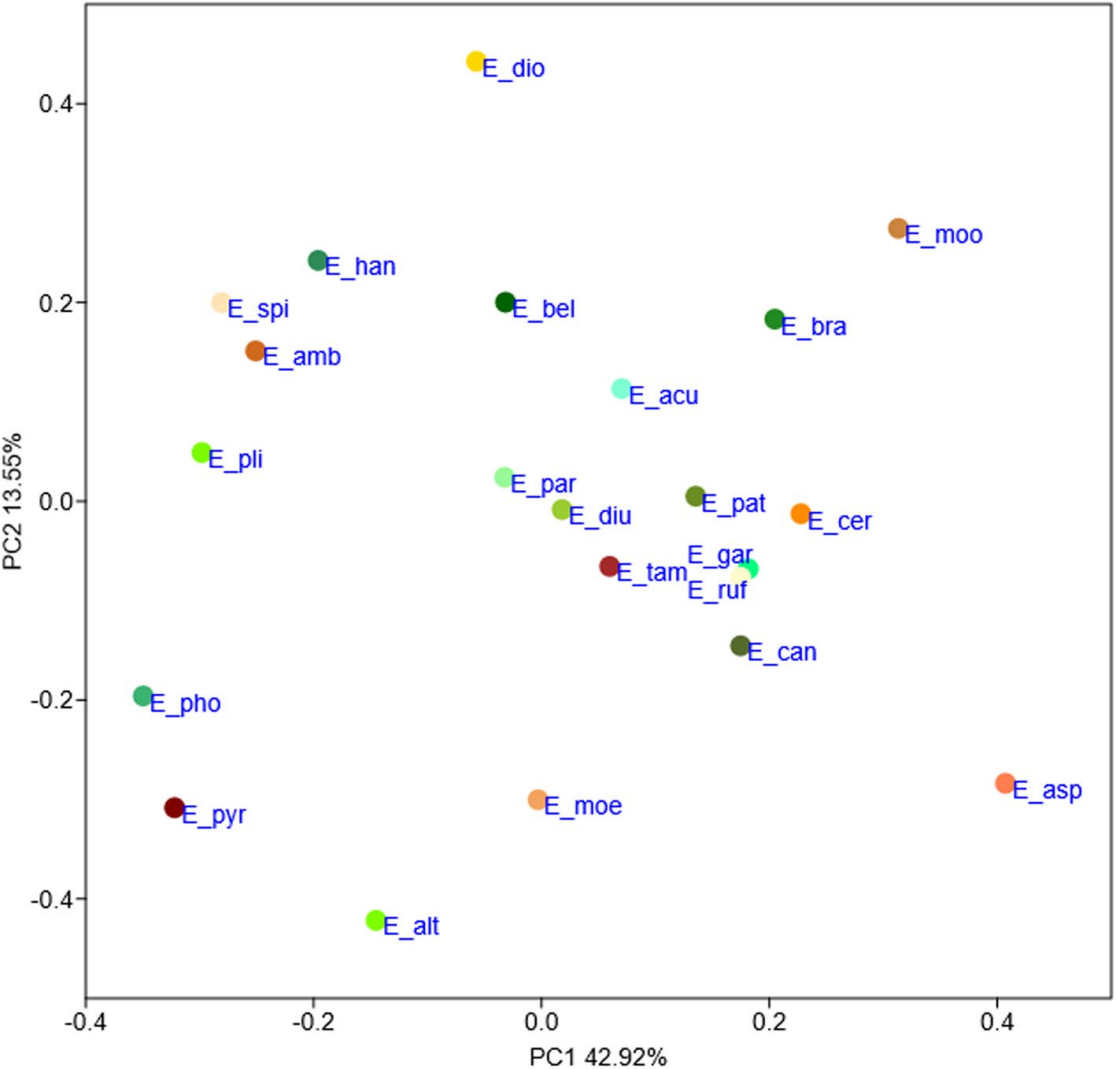
Principal coordinate analysis (PCoA) showing a two-dimensional ordination of 22 *Encyclia* species, based on the combined three data sets (floral, micro- and macromorphological features). Codes for the studied species are given in Table S5 in Additional file 2

The morphological variation of *Encyclia* species shown on the PCA plot was rather substantial based on floral features (the cumulative percentage of explained variance was 82%). However, the specimens did not form a definite grouping pattern and differed the most in terms of the petals, dorsal and lateral sepals length (DS4, LS4, PL4), the widest part of the middle lobe (LIP4), and the length of the lip (LIP7) (Fig. 6A). This is owing to the high amount of minor attributes in the ordination performed, as well as the taxa’s great morphological resemblance, where their ranges of morphological variation coincided. Although, the length of the lip (LIP7) had the largest range of variation for the recognized taxa (Fig. 6B). Along the second PCA principal component (PC2), on the right side of the scatter plot, there is a group of species that had the highest average values for most of the measured floral traits (i.e., *E. altissima*, *E. phoenicea*, *E. ambigua*, *E. hanburyi*, *E. spiritusanctensis*, *E. plicata*, *E. pyriformis*) (Table S5 in Additional file 2). This observation was also confirmed by PCoA, based on 22 *Encyclia* species and for the three combined data sets (i.e. floral, micro- and macromorphological characters) (Fig. 7). Also here, along the second axis (PC2) on the left side of the plot were the species mentioned above. SIMPER analysis identified the traits that were primarily responsible for the observed differences between species, and these were the same features which were also highlighted in the PCA analysis (LIP4, LIP7, PL4, DS4, LS4, in order of contribution, respectively; see Table 1).

## Discussion

### Phylogeny versus morphology

*Encyclia* is a genus with recent divergence [8, 19], resulting in modest sequence divergence in the most loci used for phylogenetic inference. The genus consists of closely related species, forming aggregations of morphologically similar taxa, with a similar geographical distribution. These species are often only distinguishable by careful analysis based on a combination of morphological, ecological and distributional traits.

In the most recent and comprehensive study of the genus *Encyclia* published by Carnevali et al. [20] the authors examined 106 species, representing approximately 60% of the genus content. They based their phylogenetic inferences on the analysis of the following markers: nrITS and plastid *rpl*32*-trn*L, *trn*L*-trn*F*,* and *ycf*1. The authors distinguished two main phylogeographic clades: the Northern Hemisphere clade (including Megamexican species, Caribbean species, and Eastern South American species) and the Southern Hemisphere alliance (with 3 Brazilian clades and the Andean clade). Apart from these two main groups, they defined three additional clades, i.e. the *E. microbulbon* clade, the *E. adenocarpos* clade, and the *E. ceratistes* alliance. Our phylogenetic analyses were extended to include the low-copy genes such as *Xdh* and *Phy*C, and the topology of the phylogenetic tree obtained differs in many cases. The first diverging lineage appears to be *E. bractescens* (C2, Fig. 2), which is sister to all other species of the genus. Then the tree splits into two main clades marked as 1 and 2 (Fig. 2). The first, with strong support (PP/BS = 1/1), includes most of the same species that Carnevali et al. placed in the *E*. *microbulbon* clade. Exceptions are *E*. *nizandensis* and *E*. *tuerckheimii*, which in our analyses formed clade together with the clade species *E*. *adenocarpos *sensu Carnevali et al. [20] (2.1, Fig. 2). In addition, taxa of clade 2.1 (Fig. 2) are in the sister group to other analysed species of *Encylia*, forming a clade 2.2 (Fig. 2) with the strong support of posterior probability (PP = 0.98). Within clade 2.2, it is difficult to determine the relationships between the species studied due to the lack of strong support at the nodes of the larger groupings. Some of the clades distinguished by Carnevali et al. were not confirmed in our analyses. In fact, the clades Aptera I and II, Brazil I, II, and III sensu Carnevali are formed by the same species, but without sufficiently strong support at the nodes. It is interesting to note that the most basal species of the Andean and Brazilian groups is *E. remotiflora*, which is one of the basal species of *E. ceratistes* alliance in Carnevali et al. [22]. According to these authors, this position is occupied by *E*. *betancourtiana*, a species that in our analyses is placed together with Brazil III (part1).

Subclade 2.3 (Fig. 3) is the only strongly supported and large group (PP = 1) within clade 2.2 (Fig. 2), which includes species with the *E*. *diurna* clade, *E*. *ceratistes* alliance, Brazil II (part 2), and the last Caribbean clade sensu Carnevali et al. All except the Brazilian II (part 2) have strong support at the nodal point.

The results of our studies confirm that the evolution of *Encyclia* has begun in Mesomerica (Megamexico + Central American Isthmus). The basal groups within the *Encyclia *sensu stricto, clade C2 (Fig. 2), represent *E*. *bractescens* and *E*. *microbulbon* clade (clade 1, Fig. 2) in our analyses, and they include only representatives from Mesoamerica. Probably some of the species from the Aptera I and II placed in clade 2 (Fig. 2) migrated to South America, giving rise to the Brazilian and Andean species. This is likely because both clades include Mesoamerican, Brazilian, and Andean species. On the other hand, the migration could have occurred in other ways. The species that colonized Mesoamerica could have jumped across the Antilles to the NE part of South America. This would mean that the Brazilian species originated from at least two waves of migration. This is confirmed by the Brazilian II (part 2) clade (Fig. 3), which includes not only Brazilian species. It also includes *E*. *stellata* (Mesoamerican species), *E*. *tampensis* (Antillean species), and *E*. *oncidioides* (found in Mesoamerica, Brazil, and the Andes). In addition, the *E*. *ceratistes* alliance clade (Fig. 3), which includes mostly species from Mesoamerica, also includes *E*. *ceratistes* (Andean and Mesoamerican species) and *E*. *gravida* (species found in the Antilles and Mesoamerica). While the Caribbean clade (Fig. 3) includes taxa from the Antilles as well as species from Mesoamerica and the Andes (*E*. *alata*, *E*. *guatemalensis*, *E*. *belizensis*, *E*. *parviflora*). Phylogenetic analyses strongly suggest that species, shortly after establishing in one area, began to colonize neighbouring regions. All these migrations between geographical areas greatly confuse the pattern of affinities between species groups.

Our UPGMA cluster analysis of *Encyclia *sensu stricto for seven qualitative macromorphological characters clearly divides the genus into two main groups, the larger of which is further divided into two subgroups. However, none of them coincides with any of the phylogeographic units distinguished either by Carnevali et al. [20] or in this paper. It is interesting to note that the groups resulting from the UPGMA analysis cannot be defined using macromorphological features. For example, the verrucose inflorescence axis is characteristic of group A, but this character can be found also in group B in some species.

The UPGMA cluster analysis of *Encyclia *sensu stricto based on Gower’s general coefficient for seven qualitative micromorphological characters shows more complex patterns of the similarities between the species under investigation. Again, here we have a division of species into two main groups, each of which is again divided into two subgroups. The feature that divides the two main groups is the surface of the lip – glabrous versus papillose. The other micromorphological characters are in no way correlated with the above and different character states are found within the two main groups.

The pattern of similarities between species, taking into account both macro- and micromorphological features, presents an eminently mosaic character.

### Pollination, hybridization, and isolation within *Encyclia*

Some of the *Encyclia* species groups we have distinguished in phylogenetic analyses and in those based on morphological characters, consist of vegetatively and florally similar taxa, differing in minor details of perianth structure (mainly labellum) or colouration. This pattern strongly supports a group of species that have recently evolved and continue to undergo evolutionary diversification, with some species likely to diversify among themselves and probably still occasionally hybridize [20, 29–32].

The observed pattern of variation in *Encyclia* species may result from, among other things, hybridization, which is directly related to the pollination process. The genus *Encyclia* includes species that are very similar in terms of floral and vegetative morphology. This floral similarity has led several authors to hypothesis that the similarities are due to their relatedness, however, recent phylogenetic analyses do not indicate this [33]. It is likely that the different floral patterns in *Encyclia* evolved independently in different parts of the Neotropics and this convergent evolution is probably due to evolution in different parts of the range, where different pollinators for each species occur [34].

For *Encyclia*, as for most representatives of the Orchidaceae, pollination plays a very important role in establishing species continuity. Yet, data about pollination and its mechanisms in the genus representatives are extremely random. Dressler [35] postulated that wasps and bees probably pollinate most of the species. Detailed information on this aspect is, however, extremely scarce and limited basically to the works of Braga [31], Janzen et al. [24], Ackerman [36], Diaz Torres & Vale González [25], and Krahl et al. [37, 38]. Janzen et al. [24] presented a series of information related to the reproductive system of *E. cordigera* in Costa Rica. They indicated that it is self-compatible and has been visited by females of *Xylocopa* spp., who have been removing pollinaria in search of the nectar. Ackerman [36] reported some information on the reproductive system of *E. krugii* (Bello) Britton & P. Wilson in Puerto Rico. He indicated that the species does not produce pollinator rewards, is visited infrequently, and matures few fruits. What is more, hand-pollinations showed that flowers are self-incompatible. Diaz Torres & Vale González [25] demonstrated in their work that *E. phoenicea* (Lindl.) Neumann is pollinated by *Xylocopa cubaecola* Lucas, which has been proven to be an efficient pollinated agent since all their visits resulted in effective pollination. The authors also demonstrate that due to the absence of nectar production or other floral rewards, the frequency of visitation of this bee is low, which also results in low reproductive success. For *E. mapuerae* (Huber) Brade *ex* Brade & Pabst, the pollinators have been identified thanks to the visits of two representatives of the order Hymenoptera, *Rubrica nasuta* (Christ.) and *Agelaia* cf. *pallipes* (Olivier). Although, the first one is considered the legitimate pollinator, and the second is only a nectar thief [33]. Later, Krahl et al. [37] observed that *E. mapuerae* has a cuniculus (nectariferous cavity parallel to the ovary) that produces sugar in small amounts. They also found that females of *Centris varia* pollinate it, which seems to be in contrast to the previous reports [33]. The visitation frequency was low in addition to the inefficient floral pollinators, and the species offered nectar in a low amount. According to van den Berg & Carnevali [1] the occurrence of pollination visits tends to be generally rare in many *Encyclia* species, which results in low fruit set. This seems to be supported by several independent research. Damon & Salas-Roblero [39] observed eight species of the genus during their flowering period and natural fruit formation was recorded only in *E. adenocarpa* (La Llave & Lex.) Schltr. (0–5%). For *E. mapuerae*, Krahl et al. [37] observed a natural fruit formation rate of 6.9% for the year 2011. In the genus, self-compatibility has already been recorded in *E. cordigera* [24] and *E. mapuerae* [37, 38], however, self-incompatibility is also recorded, as seen in *E. krugii* [36].

Hybridization contributes to speciation by generating new hybrid taxa, whereas the introgression of several loci can enhance adaptive divergence and facilitate speciation [40]. This is a typical phenomenon in angiosperm plants and has several evolutionary implications. The newly formed polyploid lineages are reproductively isolated from their diploid ancestors. In natural, mixed-ploidy populations of *Gymnadenia conopsea* (L.) R. Br., tetraploids had larger flowers with different scent bouquets than diploids, although the cytotypes differed only slightly in flower colour. In addition, tetraploids had higher reproductive success compared to diploids [41]. Another example is the deceptive orchids of the genus *Orchis* L., which are distinguished by a wide range of floral signals, such as colour and scent. The fragrance composition of *O. mascula* L., *O. pauciflora* Ten. and their hybrid, *O.* × *colemanii* Cortesi had a significant level of quantitative and qualitative diversity. The aroma compositions of the species were significantly distinguished, but the majority of hybrid individuals produced an intermediate odour [42].

Distinct phenotypes observed within *Encyclia *sensu stricto may include crucial innovations in flower morphology such as: (1) floral traits directly involved in reproductive isolation, (2) traits associated with pollinator shifts [43, 44], (3) traits known to be linked to differential adaptation and thus to ecological speciation [45, 46], and (4) simple interspecific differences [47], many of which are now maintained by divergent natural selection [48, 49]. Morphological variations are usually thought to be adaptive [50]. Natural selection within populations must be strong enough to overcome gene flow from morphologically different groups in order to sustain phenotypic diversity [51]. Local adaptation, on the other hand, necessitates a species expressing many morphotypes, each of which has higher fitness in its microhabitat than in other niches [52, 53].

The presented study is the first one to investigate the micromorphology of *Encyclia* species*.* Bearing this in mind, we have described in detail the structures observed on the lip surfaces of the examined species (see Additional file 3). Features that undoubtedly caught our attention were trichomes, stomata, and residues of secretion as all of them can be directly associated with the increased attractiveness for the pollinators. The first ones were present on the lip surface of eight species (see Additional file 1; Fig. S1). Trichomes of four species, namely *E. ambigua*, *E. bracteata*, *E. nematocaulon*, and *E. pollardiana*, resembled by their external appearance as food hairs. Indeed, in orchids, they usually occur on the lip surface and they contain rich reserves of food, mainly protein, and are gathered or nibbled by insects [54]. Food hairs have been previously described for e.g., genus *Maxillaria* Ruiz and Pav. and *Polystachya* Hook. [54]. Naturally, to be able to verify if observed trichomes are indeed food hairs, histochemical tests, and field observations would need to be conducted in the future. Another interesting aspect is the presence of floral stomata that were observed in 15 species. Their presence in other plant groups has been associated with nectar excretion. In floral nectaries, secretion is performed mainly by a subepidermal secreting-parenchyma, the secretion is then usually accumulated in intercellular spaces and is exuded to the external surface through stomata [55, 56]. Indeed, in some investigated species (see Additional file 1; Fig. S2A, C), stomata are visibly covered with secreted material. However, Hew et al. [57] stated that orchid floral stomata are non-functional thus with similar rates of transpiration in light and in dark observed in orchid flowers, what would account for the observations that the transpiration rate of orchid flowers is comparable to the cuticular transpiration of leaves. On the other hand, more modern research states that the volatilization of fragrance compounds in some orchid species seems to be associated with the occurrence of floral stomata (e.g. [58]). Stomatal pores are frequently observed on the surface of the nectaries that are involved in exogenous secretion (e.g. [59]), and as Vogel [59] suggests they could work as possible routes for volatile secretions. De Melo et al. [58] found evidence that the secretion products of species of *Acianthera* Scheidw. are liberated by the cells of the osmophores and accumulated in the periplasmatic and intercellular spaces. These compounds are probably volatilized by daylight temperatures and finally reach the outside environment through the stomatal pores. The scents and chemical compositions of only three species of *Encyclia *sensu stricto have been investigated so far and two of them were studied in presented research. These are *E. adenocarpa* [60], which gives off a particularly ionone-rich scent due to the presence of β-ionone and its derivatives, and *E. cordigera* [61] with scent characterized as a blend of ionone-floral and aromatic-floral notes. In none of them, the floral stomata were present. However, almost all species (14 out of 15) in which we have observed the presence of stomata, are known to be fragrant, usually strongly. It is possible then that the scent emission may occur in different ways within *Encyclia* representatives, one of which could possibly be via stomata. If the floral stomata in *Encyclia* species can be associated with scent emission, or in other words the function of osmophores, or with nectar production, should be further investigated with both transmission electron microscopy and GC–MS analysis.

## Conclusion

Our study did not provide a clear answer on how the classification of *Encyclia* should be resolved. Macromorphological characters clearly divided it into two main groups, however, these groups do not coincides with any of the phylogeographic units distinguished either by Carnevali et al. [20] or in our paper. Nevertheless, our study indicates that this topic should be further investigated – with bigger number of species investigated and perhaps, with a wider selection of molecular markers.

## Materials and methods

### Plant materials

Plant material for micromorphological and molecular analyses has been obtained from the collections of Peter Szebó (Hungary) and the Botanical Garden belonging to the University of Vienna (Austria). For morphological research, samples were examined in accordance with standard procedures. The initial phase involved digitally photographing the material and building a database with the data from the labels. Using a stereoscopic microscope, the blooms were separated from the inflorescence and thoroughly examined (Additional file 4). If possible, a larger sample size of flowers has been investigated in cases where there are questions about a specific trait. Each perianth component was precisely measured, shown, and discussed. The lip and the column received extra attention during the investigation. Particular focus has been placed on the morphology of the lip`s shape, nervousness, size, and the presence of various kinds of swellings, folds, and narrowing, and column’s viscidium, rostellum, clinandrium, and ratios of the foot’s length to the column, presumably indicating its adherence with the perianth’s lateral outer petals.

The gathered data has been compared to type specimens, diagnoses, and original drawings if any were accessible. Leaf, inflorescence architecture, floral bract size and form, flower morphology, and gynostemium structure were all evaluated and compared to type materials of other members of the genus for both vegetative and generative characteristics. The majority of the reference materials were collected and analysed while visiting herbaria in Europe and America: AMES, B, C, CAY, COL, HJBG, HUA, K, MA, MO, NY, P, RPSC, QCA, QCNE, US, VEN, W, WU (acronyms adopted from Index Herbariorum, [62]).

### Micromorphology

Lips from flowers of 61 species classified in *Encyclia *sensu stricto were studied. Formal identification of the plant material has been performed by Dariusz L. Szlachetko, Peter Szebó and Monika M. Lipińska. The flowers were preserved in Kew Mix (53% ethanol:5% formaldehyde:5% glycerol:37% water) and deposited in the Department of Plant Taxonomy and Nature Conservation of the University of Gdańsk (UGDA). The list of vouchers is presented in the Additional file 5 (Table S6). Floral material preserved in Kew Mixture was dehydrated using an ethanol series. Following critical-point drying in a Critical Point Dryer Emitech K850 apparatus, specimens were mounted onto SEM stubs. The stubs were coated with gold using a Sputter Coater SpiModule. The samples were examined and photographed using a Philips XL-30 Scanning Electron Microscope. In the SEM study, the terminology of surface characters was used in accordance with previously published literature (e.g. [63–65]).

Samples for the scanning electron microscopy (SEM) were preserved in 2,5% (*v*/*v*), glutaraldehyde (GA) in 0,05 M cacodylate buffer (pH 7,0). Following dehydration in an ethanol series, they were dried by the critical point method using liquid CO_2_, coated with gold and observed by means of a Philips XL-30 scanning electron microscope.

### Analysis of morphological variation

To describe morphological variation within *Encyclia*, three sets of characters were used: 1) seven micromorphological traits; 2) seven macromorphological traits; and 3) 22 floral morphometric features. According to Table S2 (Additional file 2), the seven qualitative features in micromorphology were coded with consecutive numbers. Taxonomic similarity and relationships were calculated using Gower’s general similarity coefficient [66] and displayed as a dendrogram. The UPGMA method was used to cluster taxa based on their micromorphological similarities. A data matrix for 61 *Encyclia* species was created for this section of the calculation (Table S3 in Additional file 2). The seven characters in macromorphology were also qualitative and were coded exclusively for the presence (1 – yes) or absence (0 – no) of a specific feature. Similarity and cluster analyses were computed in the same way as previously. The data matrix was created for 57 *Encyclia* species (Table S4 in Additional file 2). In addition, a cluster analysis based on Gower’s overall similarity coefficient for micro- and macromorphological features was performed.

Only 22 *Encyclia* species were evaluated in the flower morphometric investigation, with measurements obtained for 2–4 individuals. 57 specimens were evaluated to define patterns of morphological variation based on 22 floral features (Table S2 and Fig. S11 in Additional file 2). All investigated features were quantitative, and they were all included in the principal component analysis (PCA) and visualized using a radar chart, which allows the representation of multidimensional data in the form of a two-dimensional chart for quantitative variables, presented on axes starting from the same point [67]. Furthermore, a principal coordinate analysis (PCoA) was performed on the combined data which included: mean values for the 22 measured floral characters (Table S5 in Additional file 2); as well as 7 qualitative micro- and macromorphological features that could be assigned to the 22 species studied here. Gower’s similarity matrix was used to perform taxonomic location ordinations.

We used a percentage similarity distribution approach (SIMPER) [68] to estimate the average percentage of each variable’s dissimilarity between specimens in the Bray–Curtis similarity matrix for each set of analysed features. This allows us to identify the variables that are most likely to contribute to species differences.

All multivariate analyses were performed using software packages: STATISTICA v. 13 (TIBCO Software Inc., 2017 [69]) and PAST v. 4.03 [70].

### DNA extraction and amplification

DNA was extracted using the Genomic Mini Plant Kit (A&A Biotechnology, Poland). The extraction was performed according to the manufacturer’s protocol. The pairs of the same primers for each marker were used for amplification and sequencing. The 101F and 102R [71] for the nrITS (ITS1 + 5.8S + ITS2), and 551F and 1591R [72] for the *Xdh* gene. The PCR reaction was performed using a commercial kit (MyTaq HS DNA Polymerase Mix; BIOLINE Ltd., UK) for a total of 25 µl containing 1 µL template DNA (~ 10–100 ng), 1 µL of 10 µM of each primer, 11.5 µL Polymerase Mix, and water. Amplification parameters for the ITS marker were the following: 94 °C, 4 min; 30 × (94 °C, 45 s; 52 °C, 45 s; 72 °C, 1 min); and 72 °C, 7 min. However, for the low-copy gene *Xdh* we used a touchdown method. The initial denaturation (95 °C, 5 min) was followed by 7 cycles of 94 °C, 45 s; 59 °C, 45 s (reducing 1 °C per cycle); 72 °C, 90 s. The next step involved 30 × (94 °C, 45 s; 52 °C, 45 s; 72 °C, 90 s) and 72 °C, 7 min.. Then the obtained products were purified with Clean-Up Concentrator Kit (A&A Biotechnology, Poland) and sequenced by Macrogen (Seoul, South Korea – http://dna.macrogen.com/eng/).

### Phylogenetic analysis and molecular clock

The Finch TV (https://digitalworldbiology.com/FinchTV) was used to verify the quality of obtained chromatograms. The sequences were aligned using Mafft v. 7 [73]. In addition, minor errors were corrected in SeaView v. 5.0 [74]. Highly variable and ambiguously aligned characters of *trn*L-*trn*F were excluded from the analysis. The phylogeny and divergence time estimates of the studied group were reconstructed based on nuclear Internal Transcribed Spacer (ITS), low-copy nuclear genes: *Xdh* and *Phy*C, and several plastid markers: *mat*K*, ycf*1*, rpl*32 and *trn*L*-trn*F*.* We used both sequences available from GenBank and newly obtained sequences (mainly of *Xdh* and some of ITS). All accession numbers are given in the tables in Additional file 6, where the new accessions (from this research; OQ867206–OQ867219, OQ918681–OQ918694) are included in the second tab (Table S10).

*Arpophyllum giganteum* was chosen as an outgroup based on the results of Givnish et al. [28], where the *Arpohyllum* sample represents the basal taxa of the Laeliinae clade. In the first step of our analysis, two datasets were prepared: nuclear-combined (3656 bp) and plastid-combined (4298 bp), and then analysed.

Models of nucleotide substitution were calculated using the PhyML website (http://www.atgc-montpellier.fr) and were based on AIC (Akaike Information Criterion). The following models: HKY+G+I (nuclear-combined dataset) and GTR+G+I (plastid-combined dataset) were selected as the best ones for the studied datasets.

The relationships within the studied group were tested using different phylogenetic methods. We performed the maximum likelihood analyses using raxmlGUI 2.0 [75], with 1000 bootstrap replicates. Additionally, the bootstrap analysis, with 500 replicates (and maximum 100 trees per replication) was performed in PAUP v. 4.0. The BS (bootstrap support) values higher than 75 were considered as reliable ones. The Bayesian reconstruction was performed in MrBayes 3.2.7a using Markov Chain Monte Carlo (MCMC) on CIPRES Science Gateway [76]. Two simultaneous runs of four chains for 20 million generations, sampling one of each 100 trees were performed until the average standard deviation of split ranges reached a value below 0.02. Then the maximum clade credibility trees were constructed in TreeAnnotator v. 1.8.4. [77], with a burn-in of 25%. The PP (posterior probability) values equal to or greater than 95 were considered credible.

To estimate the divergence times of particular lineages within the studied group, a Bayesian uncorrelated relaxed molecular clock (lognormal) approach and the Yule model of speciation were implemented in BEAST v. 1.8.4. [77] on the CIPRES Science Gateway. The molecular clock was chosen based on the Bayes factor (2log_e_(BF)) using the marginal likelihoods of the model estimated by the stepping-stone/path-sampling methods. Evidence against the null model (with lower marginal likelihood) was estimated based on Kass and Raftery [78]. The value 2 > 2log_e_(BF) > 6 indicates positive evidence; 6 > 2log_e_(BF) > 10 indicates strong evidence and 2log_e_(BF) > 10 indicates very strong. The age of the root of the tree was set to 15.37 Mya, according to Givnish et al. [28]. Two independent runs, each with 80 million generations, were conducted. Results quality from each run was verified in Tracer v. 1.6. [79] and then the .log files were combined into the one (with burn-in = 25%) using LogCombiner v. 1.8.4 [77]. TreeAnnotator v. 1.8.4. were used to generate the maximum clade credibility tree was obtained in TreeAnnotator v. 1.8.4.

Our analysis, on only ITS, nuclear-combined or plastid-combined datasets resulted in low support of particular clades and generally high polytomy. We were not able to observe the topology conflict within the genus *Encyclia*, as indicated by Leopardi-Verde et al. [19], or between any other genera included in the analysis. Although the *P*-value obtained from the Partition-Homogenity test performed in PAUP v. 4.0 suggested the presence of the conflict, we decided to combine nuclear and plastid datasets into one matrix and perform the analysis of new, due to the high % of missing characters and parsimony uninformative characters in our matrixes. As postulated by e.g. Cunningham [80], missing data in the matrix can artificially increase the *P*-value, as same as the high number of parsimony uninformative characters [81].

### Ancestral state reconstruction of micro- and macromorphological features

The ancestral states of micromorphological and macromorphological characters were reconstructed for 47 *Encyclia* species, using the parsimony ancestral state reconstruction method, implemented in Mesquite v. 3.70 [82]. The input phylogenetic tree was obtained from the maximum likelihood analyses performed in raxmlGUI 2.0 [75]. Analysis parameters were set the same, as given above (in the section ‘Phylogenetic analysis and molecular clock’). *Arpophyllum gigantum* was again chosen as an outgroup*.* Taxa characters were coded for the presence (1 – —yes) or absence (0 – —no) of a feature and are presented in Table S11 (Additional file 7).

### Supplementary Information

**Additional file 1. **Summary of micromorphological features.** Table S1.** A summary of micromorphological features of the studied species. **Fig. S1.** Different trichome types. A - *Encyclia ambigua*; B - *E. bracteata*; C - *E. bractescens*; D - *E. hanburyi*; E - *E. incumbens*; F - *E. microtos*; G - *E. nematocaulon*; H - *E. pollardiana*. Scale bars: A, C, D - 50 μm; B, E-H - 20 μm. Phot. D. Łuszczek. **Fig. S2.** Stomata in different *Encyclia* species. A - *Encyclia adenocaula*; B - *E. alata*; C - *E. altissima*; D - *E. belizensis*; E - *E. bracteata*; F - *E. diurna*; G - *E. inaguensis*; H - *E. microtos*. Scale bars: A-C, F-H - 20 μm; D, E - 50 μm. Phot. D. Łuszczek. **Fig. S3.** Stomata in different *Encyclia* species. A - *Encyclia osmantha*; B - *E. parviflora*; C - *E. patens*; D - *E. phoenicea*; E - *E. pollardiana*; F - *E. rufa*; G - *E. selligera*. Scale bars: 20 μm. Phot. D. Łuszczek. **Fig. S4.** Residues of secretion in investigated *Encyclia* species. A - *Encyclia adenocarpa*; B - *E. adenocaula*; C - *E. altissima*; D - *E. aenicta*; E - *E. amanda*; F - *E. ambigua*; G - *E. andrichii*; H - *E. belizensis*; I - *E. bicalhoi*. Scale bars: A, E-I - 20 μm; B, C - 50 μm; D - 100 μm. Phot. D. Łuszczek. **Fig. S5.** Residues of secretion in investigated *Encyclia* species. A - *Encyclia bocourtii*; B - *E. bracteata*; C - *E. ceratistes*; D - *E. cordigera*; E - *E. diota*; F - *E. fehlingii*; G - *E. fucata*; H - *E. belizensis*; I - *E. hanburyi*. Scale bars: A, C-D, I - 20 μm; B - 200 μm; E - 500 μm; F-G - 50 μm. Phot. D. Łuszczek. **Fig. S6.** Residues of secretion in investigated *Encyclia* species. A - *Encyclia howardii*; B - *E. huertae*; C - *E. inaguensis*; D - *E. ivonae*; E - *E. meliosma*; F - *E. microtos*; G - *E. mooreana*; H - *E. naranjapatensis*; I - *E. nematocaulon*; J - *E. odoratissima*; K - *E. oncidioides*; L - *E. osmatha*. Scale bars: A-B, E-K - 20 μm; C-D - 100 μm; L - 50 μm. Phot. D. Łuszczek. **Fig. S7.** Residues of secretion in investigated *Encyclia* species. A - *Encyclia parviflora*; B - *E. patens*; C - *E. phoenicea*; D - *E. plicata*; E - *E. pollardiana*; F - *E. profusa*; G - *E. selligera*; H - *E. spiritusanctensis*; I - *E. trachycarpa*. Scale bars: A - 50 μm; B - 100 μm; C-H - 20 μm. Phot. D. Łuszczek. **Fig. S8.** Cristal-like structures (presumably crystallized waxes) found on the lip surface of some *Encyclia* species. A - *Encyclia acutifolia*; B - *E. adenocaula*; C - *E. altissima*; D - *E. microtes*; E - *E. odoratissima*; F - *E. osmatha*; G - *E. profusa*; H - *E. virens*. Scale bars: A-F, I - 20 μm; G-H - 100 μm. Phot. D. Łuszczek.

**Additional file 2. **Micro- and macromorphological variation.** Fig. S9.** UPGMA cluster analysis of *Encyclia sensu stricto* based on the Gower’s general coefficient for seven qualitative macromorphological characters (according to Table S4 in Additional file 2). **Fig. S10.** UPGMA cluster analysis of *Encyclia sensu stricto* based on the Gower’s general coefficient for the combined 14 qualitative micro- and macromorphological traits (according to Tables S3 and S4 in Additional file 2). **Table S2.** Codes for the micromorphological characters of E*ncyclia sensu stricto* species included in the qualitive analysis. **Table S3.** Data matrix of seven micromorphological characters used in the analysis of morphological variation. Data was transformed as shown in Table S2 in Additional file 2. In turn, a detailed list of micromorphological characters for individual species is provided in Table S1 in Additional file 1. **Table S4.** Data matrix of seven macromorphological (external) characters used in the analysis of morphological variation, where 0 - feature is not present, 1 - feature is present. Lml - lip middle lobe; Lll - lip lateral lobe. **Table S5.** Average values for measured floral characters. For detailed description of traits, see Table 2 in the main text and Fig. S11 in Additional file 2. **Fig. S11.** Graphical presentation of the measured floral characters used in the multivariate analyses (see Table 2 in the main text for a detailed description of traits).

**Additional file 3.** Detailed descriptions of labellar micromorphology.

**Additional file 4. **Flowers of Encyclia species investigated in the study.** Fig. S12.** Flowers of *Encyclia* species investigated in the study: A, B – *E. acutifolia*; C – *E. adenocaula*; D – *E. alata*; E – *E. altissima*; F – *E. amanda*; G – *E. ambigua*. Phot. M. Speckmaier. **Fig. S13.** Flowers of *Encyclia* species investigated in the study: A – *E. andrichii*; B – *E. aspera*; C – *E. atrorubens*; D – *E. belizensis*; E – *E. bracteata*; F – *E. candollei*; G – *E. ceratistes*; H – *E. chapadensis*. Phot. M. Speckmaier. **Fig. S14.** Flowers of *Encyclia* species investigated in the study: A – *E. caximboensis*; B – *E. conchaechila*; C – *E. cordigera*; D – *E. cordigera fo. leucantha*; E – *E. cordigera var. rosea*; F – *E. correllii*; G – *E. cyperifolia*. Phot. M. Speckmaier. **Fig. S15.** Flowers of *Encyclia* species investigated in the study: A – *E. dichroma*; B – *E. dickinsoniana*; C – *E. diota*; D – *E. diurna*; E – *E. elegantula*; F – *E. fehlingii*; G – *E. flabellata*; H – *E. fowliei*. Phot. M. Speckmaier. **Fig. S16.** Flowers of *Encyclia* species investigated in the study: A – *E. fucata*; B – *E. garciaeesquivelii*; C – *E. granitica*; D – *E. halbingeriana*; E – *E. hanburyi*; F – *E. howardii*; G – *E. huertae*; H – *E. incumbens*. Phot. M. Speckmaier. **Fig. S17.** Flowers of *Encyclia* species investigated in the study: A – *E. ivonae*; B – *E. kennedyi* (right) and *E. adenocaula* (left); C – *E. leucantha*; D – *E. linearifolioides*; E – *E. megalantha*; F – *E. microbulbon*; G – E*. moebusii*. Phot. M. Speckmaier. **Fig. S18.** Flowers of *Encyclia* species investigated in the study: A – *E. mooreana*; B – *E. naranjapatensis*; C – *E. nematocaulon*; D – *E. oncidioides*; E – *E. osmantha*; F – *E. oxypetala*; G – *E. parviflora*; H – *E. patens*. Phot. M. Speckmaier. **Fig. S19.** Flowers of *Encyclia* species investigated in the study: A – *E. pauciflora*; B – *E. pflanzii*; C – *E. phoenica*; D – *E. plicata*; E – *E. pollardiana*; F – *E. powellii*; G – *E. profusa*; H – *E. randii*. Phot. M. Speckmaier. **Fig. S20.** Flowers of *Encyclia* species investigated in the study: A – *E. rzedowskiana*; B – *E. saltensis*; C – *E. seidelii*; D – *E. selligera*; E – *E. spiritusanctensis*; F – *E. stellata*; G – *E. tampensis*; H – *E. thienii*. Phot. M. Speckmaier. **Fig. S21.** Flowers of *Encyclia* species investigated in the study: A – *E. virens*; B – *E. sp*. Brazil. Phot. M. Speckmaier.

**Additional file 5. **Voucher information for the taxa examined.** Table S6.** Voucher information for the taxa examined in the micromorphological analysis. **Table S7.** Voucher information for the taxa examined in the molecular analysis (fresh material). **Table S8.** Voucher information for the taxa examined in the multivariate analysis.

**Additional file 6. **A list of GenBank ID numbers.** Table S9.** A list of GenBank ID numbers for all DNA sequences used in the phylogenetic analyses, both from the NCBI database and obtained by the one of the co-authors. **Table S10.** A list of GenBank ID numbers only for new DNA sequences used in the phylogenetic analyses obtained by the one of the co-authors.

**Additional file 7.** Ancestral state reconstruction of micro- and macromorphological features.** Table S11.** Data matrix for ancestral state reconstruction of micro- and macromorphological traits, where taxa characters were coded for the presence (1 - yes) or absence (0 - no) of a feature.

## Data Availability

All additional images, data, tables supporting the presented results are included as supplementary files. A list of GenBank ID numbers for all DNA sequences used in the phylogenetic analyses, both from the NCBI database (Table S9) and obtained by the one of the co-author (OQ867206-OQ867219 and OQ918681-OQ918694; Table S10), is available in the supplementary material (Additional file 6).
