# Supplementary material for: Characters evolution of Encyclia (Laeliinae-Orchidaceae) reveals a complex pattern not phylogenetically determined: insights from macro- and micromorphology
Source: BMC Plant Biol. 2023 Dec 20;23:661. doi: 10.1186/s12870-023-04664-3 (PMC10731901; doi:10.1186/s12870-023-04664-3)
Supplement: Supplementary file 1 — Additional file 1. Summary of micromorphological features. Table S1. A summary of micromorphological features of the studied species. Fig. S1. Different trichome types. A - Encyclia ambigua; B - E. bracteata; C - E. bractescens; D - E. hanburyi; E - E. incumbens; F - E. microtos; G - E. nematocaulon; H - E. pollardiana. Scale bars: A, C, D - 50 μm; B, E-H - 20 μm. Phot. D. Łuszczek. Fig. S2. Stomata in different Encyclia species. A - Encyclia adenocaula; B - E. alata; C - E. altissima; D - E. belizensis; E - E. bracteata; F - E. diurna; G - E. inaguensis; H - E. microtos. Scale bars: A-C, F-H - 20 μm; D, E - 50 μm. Phot. D. Łuszczek. Fig. S3. Stomata in different Encyclia species. A - Encyclia osmantha; B - E. parviflora; C - E. patens; D - E. phoenicea; E - E. pollardiana; F - E. rufa; G - E. selligera. Scale bars: 20 μm. Phot. D. Łuszczek. Fig. S4. Residues of secretion in investigated Encyclia species. A - Encyclia adenocarpa; B - E. adenocaula; C - E. altissima; D - E. aenicta; E - E. amanda; F - E. ambigua; G - E. andrichii; H - E. belizensis; I - E. bicalhoi. Scale bars: A, E-I - 20 μm; B, C - 50 μm; D - 100 μm. Phot. D. Łuszczek. Fig. S5. Residues of secretion in investigated Encyclia species. A - Encyclia bocourtii; B - E. bracteata; C - E. ceratistes; D - E. cordigera; E - E. diota; F - E. fehlingii; G - E. fucata; H - E. belizensis; I - E. hanburyi. Scale bars: A, C-D, I - 20 μm; B - 200 μm; E - 500 μm; F-G - 50 μm. Phot. D. Łuszczek. Fig. S6. Residues of secretion in investigated Encyclia species. A - Encyclia howardii; B - E. huertae; C - E. inaguensis; D - E. ivonae; E - E. meliosma; F - E. microtos; G - E. mooreana; H - E. naranjapatensis; I - E. nematocaulon; J - E. odoratissima; K - E. oncidioides; L - E. osmatha. Scale bars: A-B, E-K - 20 μm; C-D - 100 μm; L - 50 μm. Phot. D. Łuszczek. Fig. S7. Residues of secretion in investigated Encyclia species. A - Encyclia parviflora; B - E. patens; C - E. phoenicea; D - E. plicata; E - E. pollardiana; F - E. [file 12870_2023_4664_MOESM1_ESM.pdf]

**Table S1** A summary of micromorphological features of the studied species

| | Species | Lip surface | Lateral lobes | Callus | Middle lobe | Secretion | Stomata | Trichomes | Cuticle |
|---|---|---|---|---|---|---|---|---|---|
| 1 | *Encyclia acutifolia* | Papillose | Obpyriform papillae | Glabrous | Obpyriform papillae | Not present | Not present | Not present | Striate |
| 2 | *Encyclia adenocarpa* | Papillose | Conical papillae | Conical papillae | Conical, villiform papillae | Present | Not present | Not present | Striate |
| 3 | *Encyclia adenocaula* | Papillose | Conical papillae | Conical papillae | Conical papillae | Present | Present | Not present | Striate |
| 4 | *Encyclia aenicta* | Papillose | Glabrous | Villiform papillae | Glabrous | Present | Not present | Not present | Striate |
| 5 | *Encyclia alata* | Glabrous | Glabrous | Glabrous | Glabrous | Not present | Present | Not present | Striate |
| 6 | *Encyclia altissima* | Papillose | Obpyriform papillae | Glabrous | Obpyriform papillae | Present | Present | Not present | Striate |
| 7 | *Encyclia amanda* | Glabrous | Glabrous | Conical, villiform papillae | Glabrous | Present | Not present | Not present | Striate |
| 8 | *Encyclia ambigua* | Papillose | Obpyriform papillae | Obpyriform papillae | Conical, obpyriform papillae | Present | Not present | 1-2 celled | Striate |
| 9 | *Encyclia andrichii* | Papillose | Glabrous | Conical, obpyriform papillae | Glabrous | Present | Not present | Not present | Striate |
| 10 | *Encyclia aspera* | Glabrous | Glabrous | Glabrous | Glabrous | Not present | Not present | Not present | Striate |
| 11 | *Encyclia asperula* | Glabrous | Glabrous | Glabrous | Glabrous | Not present | Not present | Not present | Striate |
| 12 | *Encyclia belizensis* | Papillose | Obpyriform, conical papillae | Obpyriform papillae | Obpyriform papillae | Present | Present | Not present | Striate |
| 13 | *Encyclia bicalhoi* | Glabrous | Glabrous | Glabrous | Glabrous | Present | Not present | Not present | Striate |
| 14 | *Encyclia bocourtii* | Glabrous | Glabrous | Glabrous | Glabrous | Present | Not present | Not present | Striate |
| 15 | *Encyclia bracteata* | Glabrous | Glabrous | Villiform papillae | Glabrous | Present | Present | Multicellular | Striate |
| 16 | *Encyclia bractescens* | Papillose | Glabrous | Obpyriform, villiform papillae | Obpyriform papillae | Not present | Not present | 1-celled | Striate |
| 17 | *Encyclia candollei* | Glabrous | Glabrous | Conical, villiform papillae | Glabrous | Not present | Not present | Not present | Striate |
| 18 | *Encyclia ceratistes* | Glabrous | Glabrous | Glabrous | Glabrous | Present | Not present | Not present | Striate |
| 19 | *Encyclia chapadensis* | Glabrous | Glabrous | Glabrous | Glabrous | Not present | Not present | Not present | Striate |
| 20 | *Encyclia cordigera* | Papillose | Conical papillae | Conical, villiform papillae | Conical, obpyriform papillae | Present | Not present | Not present | Striate |

| | | | | | | | | | |
|---|---|---|---|---|---|---|---|---|---|
| 21 | *Encyclia dichroma* | Papillose | Conical papillae | Conical papillae | Conical, villiform papillae | Not present | Not present | Not present | Striate |
| 22 | *Encyclia diota* | Papillose | Conical papillae | Villiform papillae | Glabrous | Present | Not present | Not present | Striate |
| 23 | *Encyclia diurna* | Glabrous | Obpyriform papillae | Glabrous | Glabrous | Not present | Present | Not present | Striate |
| 24 | *Encyclia fehlingii* | Glabrous | Glabrous | Glabrous | Glabrous | Present | Not present | Not present | Striate |
| 25 | *Encyclia fucata* | Papillose | Obpyriform papillae | Conical papillae | Obpyriform papillae | Present | Not present | Not present | Striate |
| 26 | *Encyclia garciae-esquivelii* | Glabrous | Glabrous | Obpyriform papillae | Glabrous | Not present | Not present | Not present | Striate |
| 27 | *Encyclia guatemalensis* | Papillose | Glabrous | Glabrous | Glabrous | Not present | Not present | Not present | Striate |
| 28 | *Encyclia hanburyi* | Papillose | Conical papillae | Conical, villiform papillae | Conical papillae | Present | Not present | 1-celled | Striate |
| 29 | *Encyclia howardii* | Glabrous | Glabrous | Glabrous | Glabrous | Present | Not present | Not present | Striate |
| 30 | *Encyclia huertae* | Glabrous | Glabrous | Conical papillae | Glabrous | Present | Not present | Not present | Striate |
| 31 | *Encyclia inaguensis* | Glabrous | Glabrous | Glabrous | Glabrous | Present | Present | Not present | Striate |
| 32 | *Encyclia incumbens* | Papillose | Conical, obpyriform papillae | Conical, villiform papillae | Conical, obpyriform papillae | Not present | Not present | 2-celled | Striate |
| 33 | *Encyclia ivonae* | Papillose | Glabrous | Glabrous | Obpyriform papillae | Present | Not present | Not present | Striate |
| 34 | *Encyclia meliosma* | Papillose | Conical papillae | Villiform papillae | Glabrous | Present | Not present | Not present | Striate |
| 35 | *Encyclia microtos* | Glabrous | Glabrous | Glabrous | Glabrous | Present | Present | 1-celled | Striate |
| 36 | *Encyclia moebusii* | Glabrous | Obpyriform papillae | Glabrous | Glabrous | Not present | Not present | Not present | Striate |
| 37 | *Encyclia mooreana* | Glabrous | Glabrous | Conical, villiform papillae | Glabrous | Present | Not present | Not present | Striate |
| 38 | *Encyclia naranjapatensis* | Papillose | Obpyriform papillae | Villiform papillae | Glabrous | Present | Not present | Not present | Striate |
| 39 | *Encyclia nematocaulon* | Papillose | Glabrous | Villiform papillae | Conical, obpyriform papillae | Present | Not present | 1-celled | Striate |
| 40 | *Encyclia oblongata* | Glabrous | Obpyriform papillae | Glabrous | Glabrous | Not present | Not present | Not present | Striate |
| 41 | *Encyclia odoratissima* | Glabrous | Glabrous | Glabrous | Glabrous | Present | Not present | Not present | Striate |
| 42 | *Encyclia oncidioides* | Glabrous | Glabrous | Glabrous | Glabrous | Present | Not present | Not present | Striate |
| 43 | *Encyclia osmatha* | Glabrous | Glabrous | Glabrous | Glabrous | Present | Not present | Not present | Striate |

| | | | | | | | | | |
|---|---|---|---|---|---|---|---|---|---|
| 44 | *Encyclia papillosa* | Papillose | Conical papillae | Conical papillae | Conical, obpyriform papillae | Not present | Not present | Not present | Striate |
| 45 | *Encyclia parviflora* | Glabrous | Obpyriform papillae | Glabrous | Glabrous | Present | Not present | Not present | Striate |
| 46 | *Encyclia patens* | Glabrous | Glabrous | Glabrous | Glabrous | Present | Not present | Not present | Striate |
| 47 | *Encyclia pauciflora* | Glabrous | Glabrous | Villiform papillae | Glabrous | Not present | Not present | Not present | Striate |
| 48 | *Encyclia phoenicea* | Glabrous | Glabrous | Glabrous | Obpyriform papillae | Present | Not present | Not present | Striate |
| 49 | *Encyclia plicata* | Glabrous | Glabrous | Glabrous | Glabrous | Present | Not present | Not present | Striate |
| 50 | *Encyclia pollardiana* | Glabrous | Glabrous | Conical, villiform papillae | Glabrous | Present | Not present | Multicellular | Striate |
| 51 | *Encyclia powellii* | Glabrous | Glabrous | Glabrous | Glabrous | Not present | Not present | Not present | Striate |
| 52 | *Encyclia profusa* | Glabrous | Glabrous | Glabrous | Glabrous | Present | Not present | Not present | Striate |
| 53 | *Encyclia pyriformis* | Glabrous | Glabrous | Glabrous | Glabrous | Not present | Not present | Not present | Striate |
| 54 | *Encyclia rufa* | Glabrous | Glabrous | Glabrous | Glabrous | Not present | Present | Not present | Striate |
| 55 | *Encyclia seidelii* | Glabrous | Glabrous | Glabrous | Obpyriform papillae | Not present | Not present | Not present | Striate |
| 56 | *Encyclia selligera* | Glabrous | Glabrous | Conical papillae | Glabrous | Present | Not present | Not present | Striate |
| 57 | *Encyclia spiritusanctensis* | Papillose | Conical papillae | Glabrous | Conical papillae | Present | Not present | Not present | Striate |
| 58 | *Encyclia tampensis* | Glabrous | Glabrous | Glabrous | Glabrous | Not present | Not present | Not present | Striate |
| 59 | *Encyclia trachycarpa* | Papillose | Conical papillae | Conical papillae | Conical, obpyriform papillae | Present | Not present | Not present | Striate |
| 60 | *Encyclia trachychila* | Papillose | Conical papillae | Conical, villiform papillae | Glabrous | Not present | Not present | Not present | Striate |
| 61 | *Encyclia virens (trachycarpa)* | Papillose | Conical, obpyriform papillae | Glabrous | Obpyriform papillae | Not present | Not present | Not present | Striate |

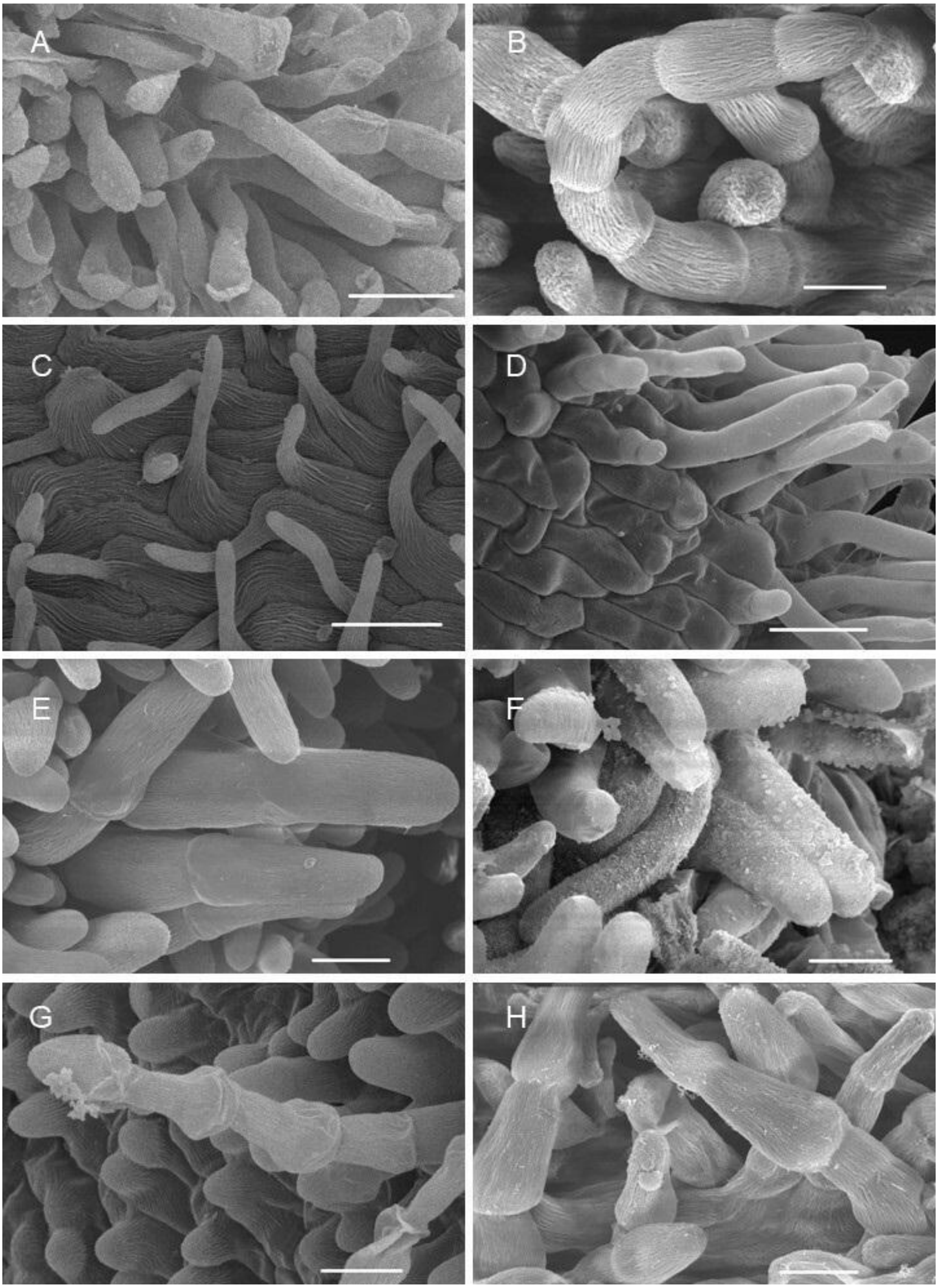


**Fig. S1** Different trichome types. **A** - *Encyclia ambigua*; **B** - *E. bracteata*; **C** - *E. bractescens*; **D** - *E. hanburyi*; **E** - *E. incumbens*; **F** - *E. microtos*; **G** - *E. nematocaulon*; **H** - *E. pollardiana*. Scale bars: A, C, D - 50 μm; B, E-H - 20 μm. Phot. D. Łuszczek

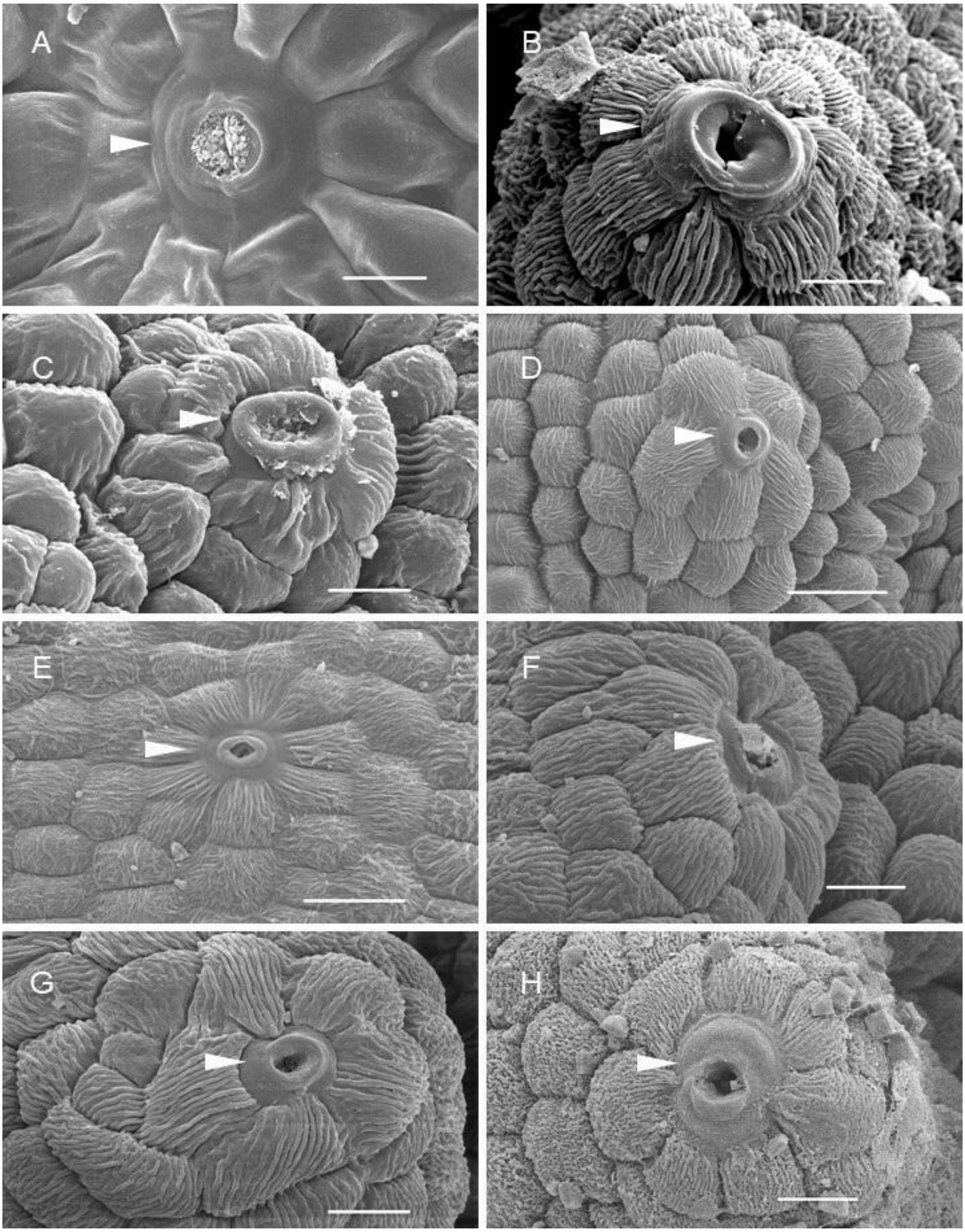


**Fig. S2** Stomata in different *Encyclia* species. **A** - *Encyclia adenocaula*; **B** - *E. alata*; **C** - *E. altissima*; **D** - *E. belizensis*; **E** - *E. bracteata*; **F** - *E. diurna*; **G** - *E. inaguensis*; **H** - *E. microtos*. Scale bars: A-C, F-H - 20 μm; D, E - 50 μm. Phot. D. Łuszczek

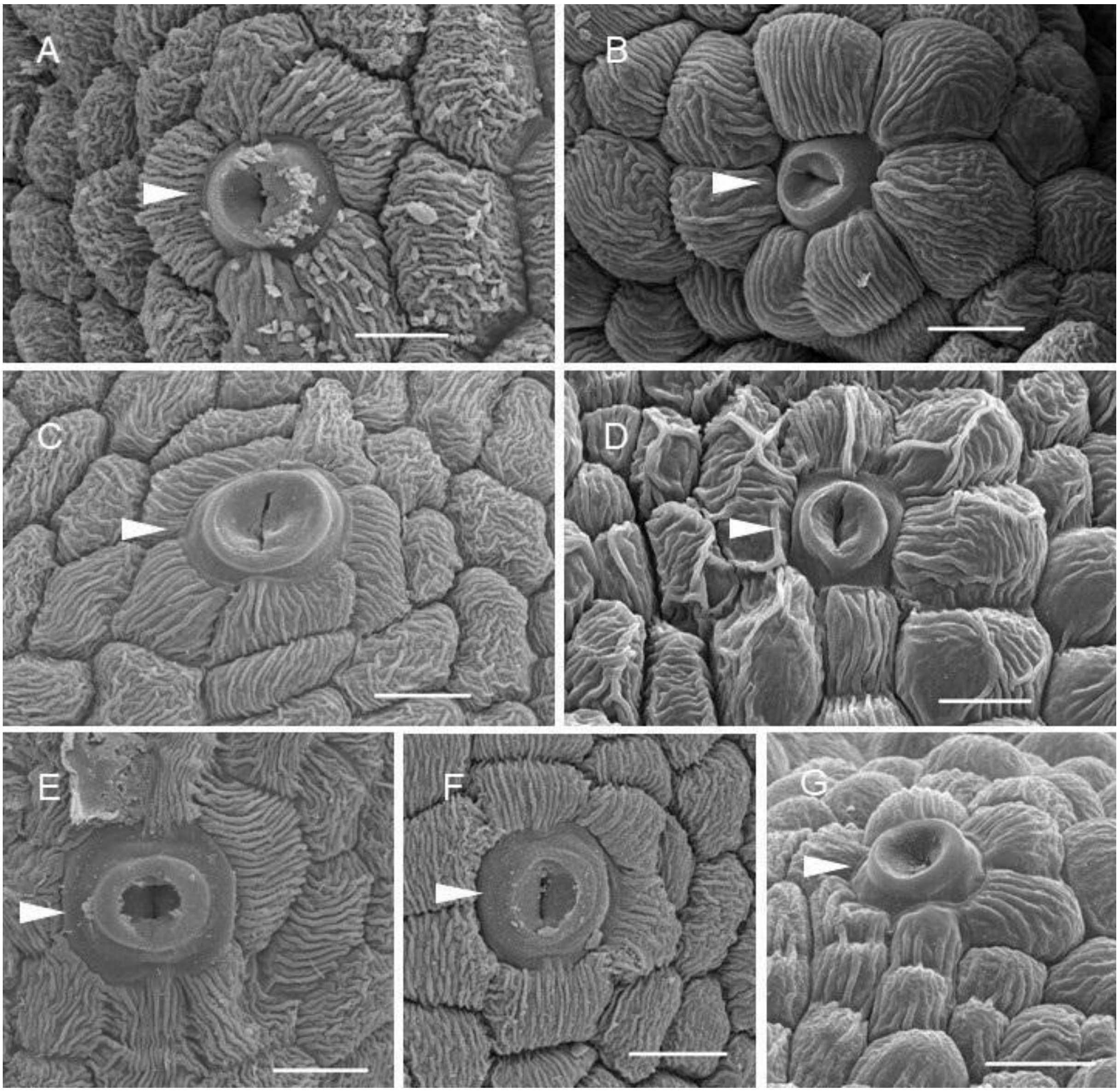


**Fig. S3** Stomata in different *Encyclia* species. **A** - *Encyclia osmantha*; **B** - *E. parviflora*; **C** - *E. patens*; **D** - *E. phoenicea*; **E** - *E. pollardiana*; **F** - *E. rufa*; **G** - *E. selligera.* Scale bars: 20 µm. Phot. D. Łuszczek

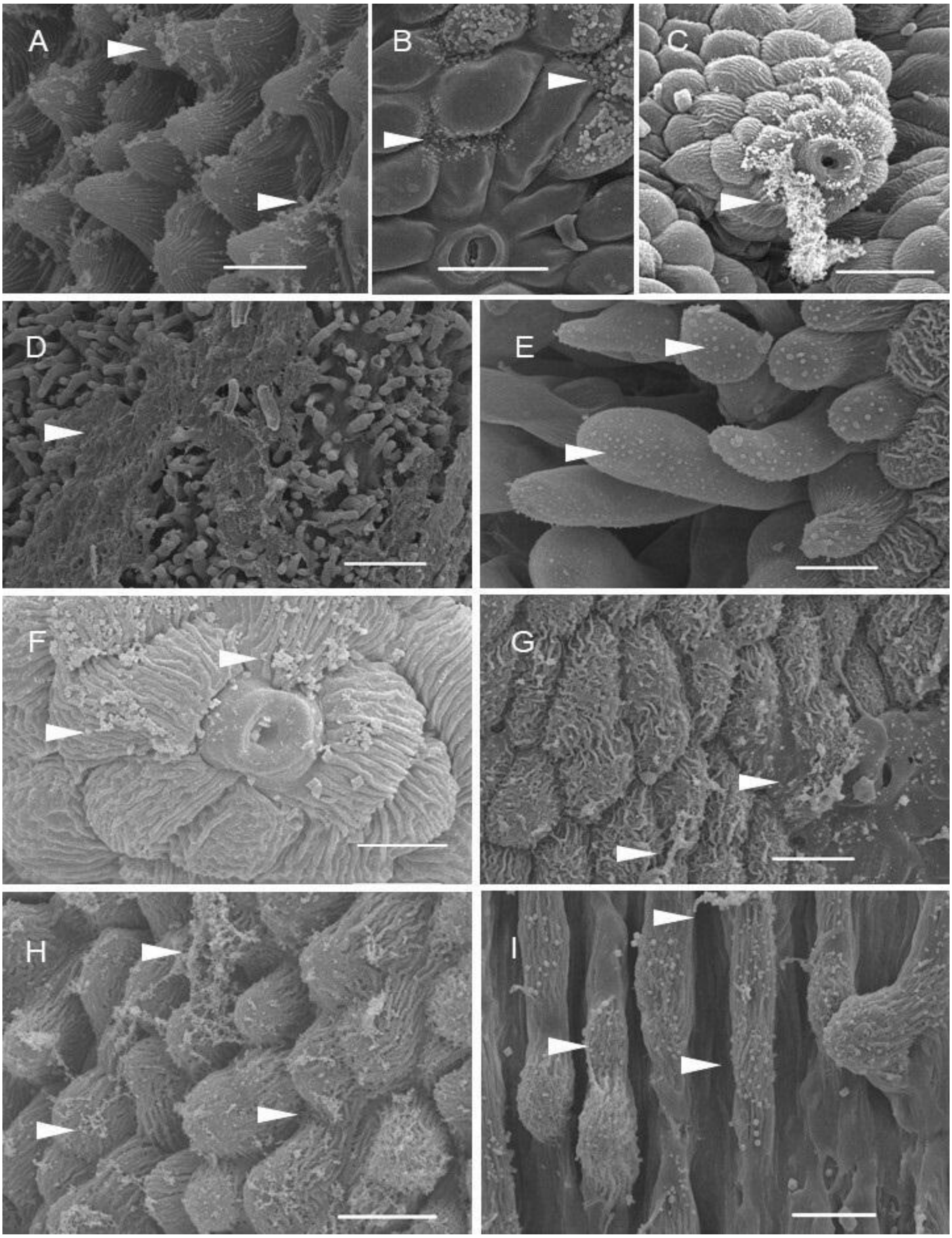


**Fig. S4** Residues of secretion in investigated *Encyclia* species. **A** - *Encyclia adenocarpa*; **B** - *E. adenocaula*; **C** - *E. altissima*; **D** - *E. aenicta*; **E** - *E. amanda*; **F** - *E. ambigua*; **G** - *E. andrichii*; **H** - *E. belizensis*; **I** - *E. bicalhoi.* Scale bars: A, E-I - 20 µm; B, C - 50 µm; D - 100 µm. Phot. D. Łuszczek

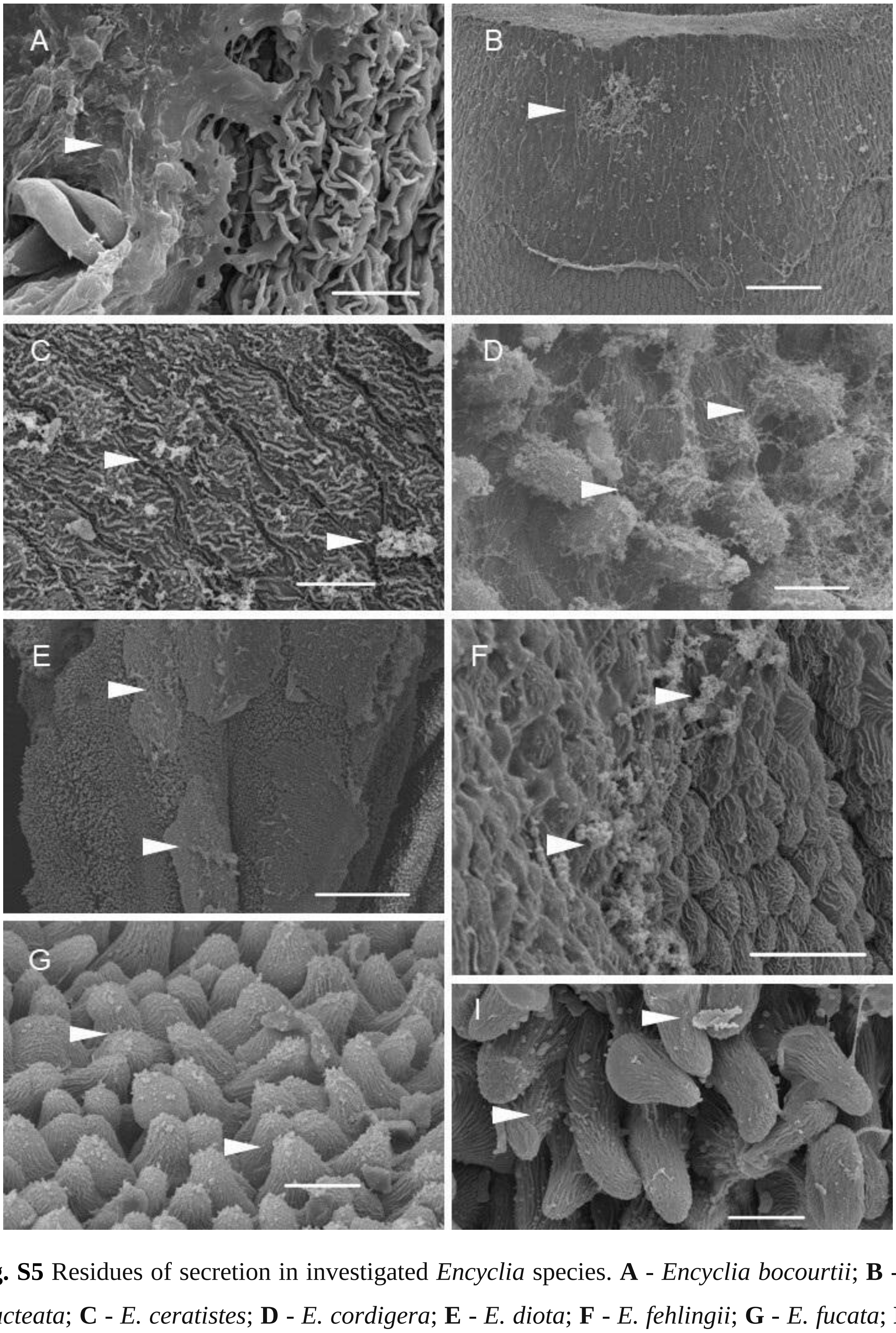


**Fig. S5** Residues of secretion in investigated *Encyclia* species. **A** - *Encyclia bocourtii*; **B** - *E. bracteata*; **C** - *E. ceratistes*; **D** - *E. cordigera*; **E** - *E. diota*; **F** - *E. fehlingii*; **G** - *E. fucata*; **H** - *E. belizensis*; **I** - *E. hanburyi*. Scale bars: A, C-D, I - 20 μm; B - 200 μm; E - 500 μm; F-G - 50 μm. Phot. D. Łuszczek

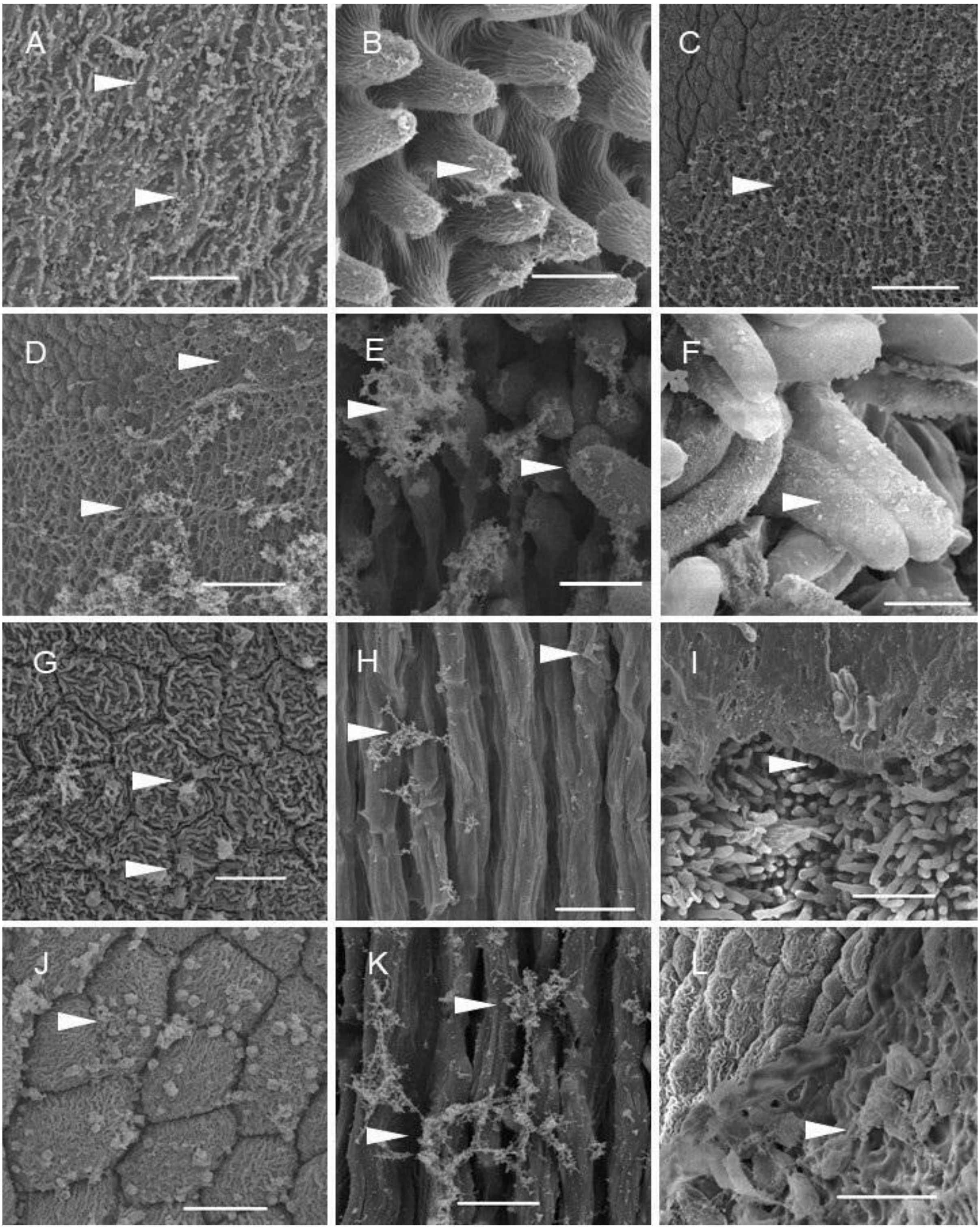


**Fig. S6** Residues of secretion in investigated *Encyclia* species. **A** - *Encyclia howardii*; **B** - *E. huertae*; **C** - *E. inaguensis;* **D** - *E. ivonae*; **E** - *E. meliosma*; **F** - *E. microtos*; **G** - *E. mooreana*; **H** - *E. naranjapatensis*; **I** - *E. nematocaulon*; **J** - *E. odoratissima*; **K** - *E. oncidioides*; **L** - *E. osmatha.* Scale bars: A-B, E-K - 20 µm; C-D - 100 µm; L - 50 µm. Phot. D. Łuszczek

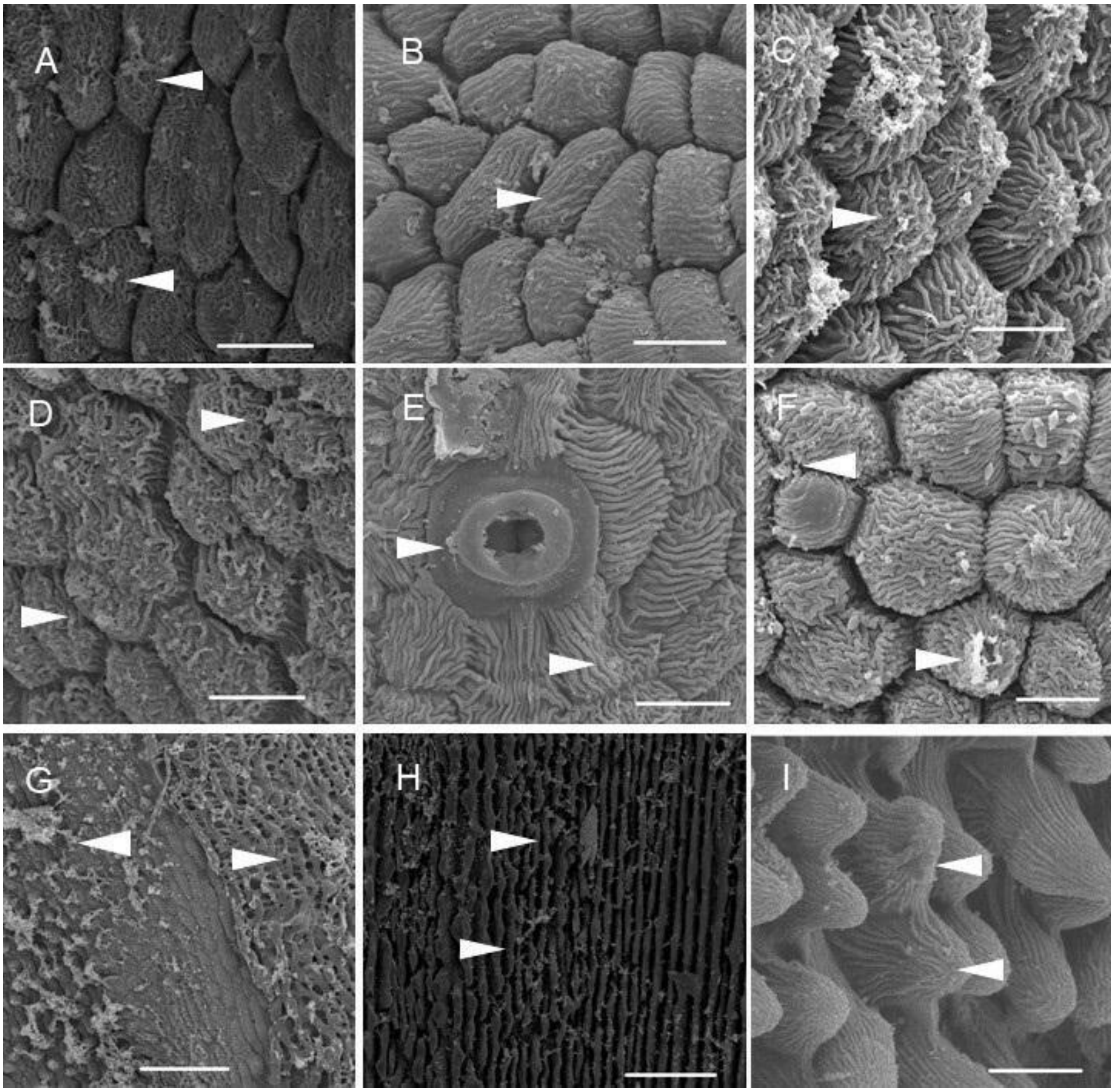


**Fig. S7** Residues of secretion in investigated *Encyclia* species. **A** - *Encyclia parviflora*; **B** - *E. patens*; **C** - *E. phoenicea*; **D** - *E. plicata*; **E** - *E. pollardiana*; **F** - *E. profusa*; **G** - *E. selligera*; **H** - *E. spiritusanctensis*; **I** - *E. trachycarpa*. Scale bars: A - 50 µm; B - 100 µm; C-H - 20 µm. Phot. D. Łuszczek

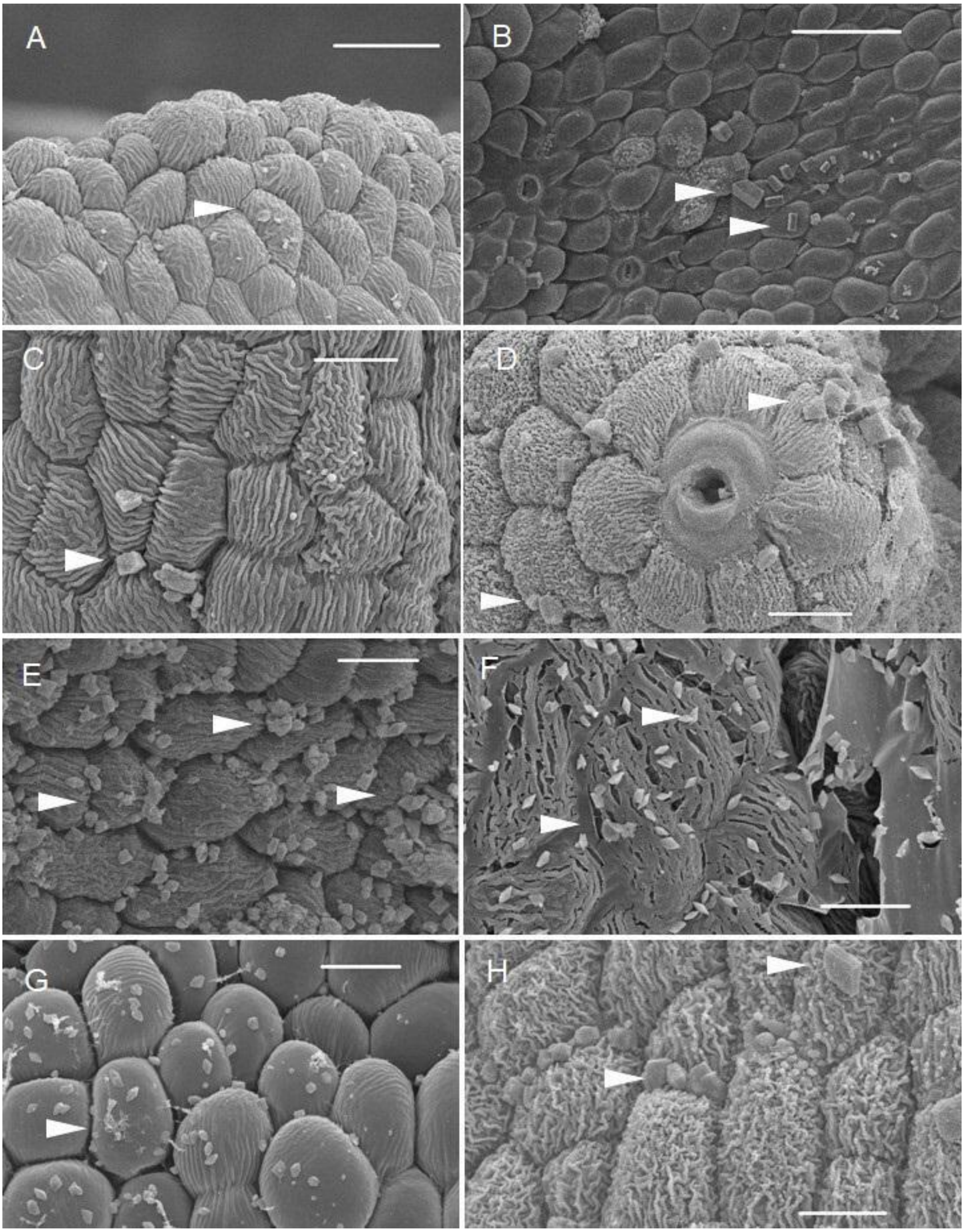


**Fig. S8** Cristal-like structures (presumably crystallized waxes) found on the lip surface of some *Encyclia* species. **A** - *Encyclia acutifolia*; **B** - *E. adenocaula*; **C** - *E. altissima*; **D** - *E. microtes*; **E** - *E. odoratissima*; **F** - *E. osmatha*; **G** - *E. profusa*; **H** - *E. virens*. Scale bars: A-F, I - 20 µm; G-H - 100 µm. Phot. D. Łuszczek
