# Supplementary material for: Characters evolution of Encyclia (Laeliinae-Orchidaceae) reveals a complex pattern not phylogenetically determined: insights from macro- and micromorphology
Source: BMC Plant Biol. 2023 Dec 20;23:661. doi: 10.1186/s12870-023-04664-3 (PMC10731901; doi:10.1186/s12870-023-04664-3)
Supplement: Supplementary file 2 — Additional file 2. Micro- and macromorphological variation. Fig. S9. UPGMA cluster analysis of Encyclia sensu stricto based on the Gower’s general coefficient for seven qualitative macromorphological characters (according to Table S4 in Additional file 2). Fig. S10. UPGMA cluster analysis of Encyclia sensu stricto based on the Gower’s general coefficient for the combined 14 qualitative micro- and macromorphological traits (according to Tables S3 and S4 in Additional file 2). Table S2. Codes for the micromorphological characters of Encyclia sensu stricto species included in the qualitive analysis. Table S3. Data matrix of seven micromorphological characters used in the analysis of morphological variation. Data was transformed as shown in Table S2 in Additional file 2. In turn, a detailed list of micromorphological characters for individual species is provided in Table S1 in Additional file 1. Table S4. Data matrix of seven macromorphological (external) characters used in the analysis of morphological variation, where 0 - feature is not present, 1 - feature is present. Lml - lip middle lobe; Lll - lip lateral lobe. Table S5. Average values for measured floral characters. For detailed description of traits, see Table 2 in the main text and Fig. S11 in Additional file 2. Fig. S11. Graphical presentation of the measured floral characters used in the multivariate analyses (see Table 2 in the main text for a detailed description of traits). [file 12870_2023_4664_MOESM2_ESM.pdf]

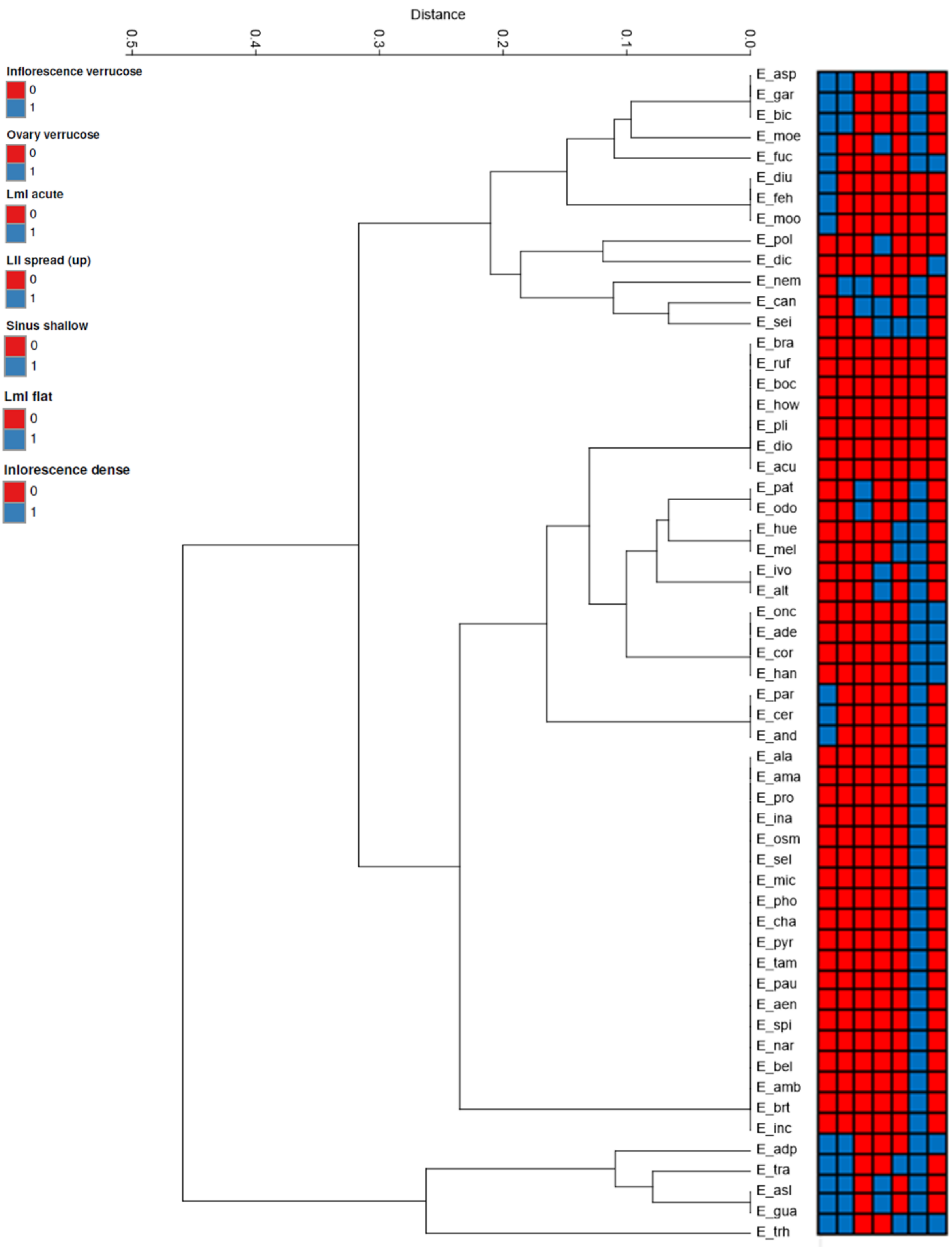

Fig. S9 UPGMA cluster analysis of *Encyclia sensu stricto* based on the Gower's general coefficient for seven qualitative macromorphological characters (according to Table S4 in Additional file 2)

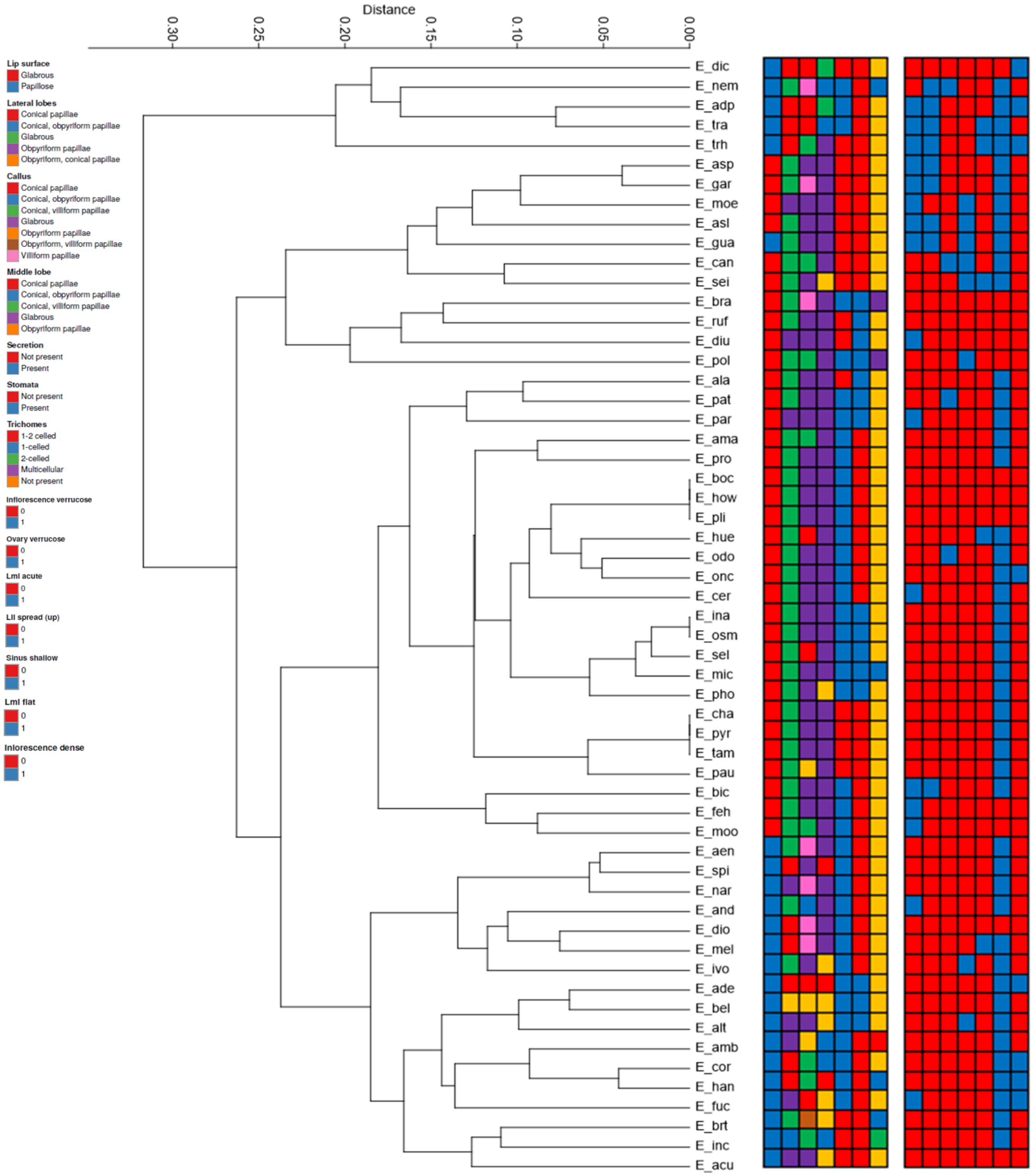

Fig. S10 UPGMA cluster analysis of *Encyclia sensu stricto* based on the Gower's general coefficient for the combined 14 qualitative micro- and macromorphological traits (according to Tables S3 and S4 in Additional file 2)

Table S2 Codes for the micromorphological characters of *Encyclia sensu stricto* species included in the qualitive analysis

**Lip surface**		**Lateral lobes**		**Callus**		**Middle lobe**		**Secretion**		**Stomata**		**Trichomes**	
Glabrous	0	Glabrous	0	Glabrous	0	Glabrous	0	Not present	0	Not present	0	Not present	0
Papillose	1	Conical papillae	1	Conical papillae	1	Conical papillae	1	Present	1	Present	1	1-celled	1
		Obpyriform papillae	2	Obpyriform papillae	2	Obpyriform papillae	2					2-celled	2
		Conical, obpyriform papillae	3	Villiform papillae	3	Conical, obpyriform papillae	3					1-2-celled	3
		Obpyriform, conical papillae	4	Conical, obpyriform papillae	4	Conical, villiform papillae	4					Multicellular	4
				Conical, villiform papillae	5								
				Obpyriform, villiform papillae	6								

Table S3 Data matrix of seven micromorphological characters used in the analysis of morphological variation. Data was transformed as shown in Table S2 in Additional file 2. In turn, a detailed list of micromorphological characters for individual species is provided in Table S1 in Additional file 1

Taxon	Code	Lip surface	Lateral lobes	Callus	Middle lobe	Secretion	Stomata	Trichomes
Encyclia acutifolia	E_acu	1	2	0	2	0	0	0
Encyclia adenocarpa	E_adp	1	1	1	4	1	0	0
Encyclia adenocaulon	E_ade	1	1	1	1	1	1	0
Encyclia aenicta	E_aen	1	0	3	0	1	0	0
Encyclia alata	E_ala	0	0	0	0	0	1	0
Encyclia altissima	E_alt	1	2	0	2	1	1	0
Encyclia amanda	E_ama	0	0	5	0	1	0	0
Encyclia ambigua	E_amb	1	2	2	3	1	0	3
Encyclia andrichii	E_and	1	0	4	0	1	0	0
Encyclia aspera	E_asp	0	0	0	0	0	0	0
Encyclia asperula	E_asl	0	0	0	0	0	0	0
Encyclia belizensis	E_bel	1	4	2	2	1	1	0
Encyclia bicalhoi	E_bic	0	0	0	0	1	0	0
Encyclia bocourtii	E_boc	0	0	0	0	1	0	0
Encyclia bracteata	E_bra	0	0	3	0	1	1	4
Encyclia bractescens	E_brt	1	0	6	2	0	0	1
Encyclia candollei	E_can	0	0	5	0	0	0	0
Encyclia ceratistes	E_cer	0	0	0	0	1	0	0
Encyclia chapadensis	E_cha	0	0	0	0	0	0	0
Encyclia cordigera	E_cor	1	1	5	3	1	0	0
Encyclia dichroma	E_dic	1	1	1	4	0	0	0
Encyclia diota	E_dio	1	1	3	0	1	0	0
Encyclia diurna	E_diu	0	2	0	0	0	1	0
Encyclia fehlingii	E_feh	0	0	0	0	1	0	0
Encyclia fucata	E_fuc	1	2	1	2	1	0	0
Encyclia garciae-esquivelii	E_gar	0	0	2	0	0	0	0
Encyclia guatemalensis	E_gua	1	0	0	0	0	0	0
Encyclia hanburyi	E_han	1	1	5	1	1	0	1
Encyclia howardii	E_how	0	0	0	0	1	0	0
Encyclia huertae	E_hue	0	0	1	0	1	0	0
Encyclia inaguensis	E_ina	0	0	0	0	1	1	0
Encyclia incumbens	E_inc	1	3	5	3	0	0	2
Encyclia ivonae	E_ivo	1	0	0	2	1	0	0

Encyclia meliosma	E_mel	1	1	3	0	1	0	0
Encyclia microtos	E_mic	0	0	0	0	1	1	1
Encyclia moebusii	E_moe	0	2	0	0	0	0	0
Encyclia mooreana	E_moo	0	0	5	0	1	0	0
Encyclia naranjapatensis	E_nar	1	2	3	0	1	0	0
Encyclia nematocaulon	E_nem	1	0	3	3	1	0	1
Encyclia oblongata	E_obl	0	2	0	0	0	0	0
Encyclia odoratissima	E_odo	0	0	0	0	1	0	0
Encyclia oncidioides	E_onc	0	0	0	0	1	0	0
Encyclia osmatha	E_osm	0	0	0	0	1	1	0
Encyclia papillosa	E_pap	1	1	1	3	0	0	0
Encyclia parviflora	E_par	0	2	0	0	1	1	0
Encyclia patens	E_pat	0	0	0	0	1	1	0
Encyclia pauciflora	E_pau	0	0	3	0	0	0	0
Encyclia phoenicea	E_pho	0	0	0	2	1	1	0
Encyclia plicata	E_pli	0	0	0	0	1	0	0
Encyclia pollardiana	E_pol	0	0	5	0	1	1	4
Encyclia powellii	E_pow	0	0	0	0	0	0	0
Encyclia profusa	E_pro	0	0	0	0	1	0	0
Encyclia pyriformis	E_pyr	0	0	0	0	0	0	0
Encyclia rufa	E_ruf	0	0	0	0	0	1	0
Encyclia seidelii	E_sei	0	0	0	2	0	0	0
Encyclia selligera	E_sel	0	0	1	0	1	1	0
Encyclia spiritusanctensis	E_spi	1	1	0	1	1	0	0
Encyclia tampensis	E_tam	0	0	0	0	0	0	0
Encyclia trachycarpa	E_tra	1	1	1	3	1	0	0
Encyclia trachychila	E_trh	1	1	5	0	0	0	0
Encyclia virens	E_vir	1	3	0	2	0	0	0

Table S4 Data matrix of seven macromorphological (external) characters used in the analysis of morphological variation, where 0 - feature is not present, 1 - feature is present. Lml - lip middle lobe; Lll - lip lateral lobe

**Taxon**	**Code**	**Inflorescence verrucose**	**Ovary verrucose**	**Lml acute**	**Lll spread (up)**	**Sinus shallow**	**Lml flat**	**Inlorescence dense**
Encyclia acutifolia	E_acu	0	0	0	0	0	0	0
Encyclia adenocarpa	E_adp	1	1	0	0	0	1	1
Encyclia adenocaulon	E_ade	0	0	0	0	0	1	1
Encyclia aenicta	E_aen	0	0	0	0	0	1	0
Encyclia alata	E_ala	0	0	0	0	0	1	0
Encyclia altissima	E_alt	0	0	0	1	0	1	0
Encyclia amanda	E_ama	0	0	0	0	0	1	0
Encyclia ambigua	E_amb	0	0	0	0	0	1	0
Encyclia andrichii	E_and	1	0	0	0	0	1	0
Encyclia aspera	E_asp	1	1	0	0	0	1	0
Encyclia asperula	E_asl	1	1	0	1	0	1	0
Encyclia belizensis	E_bel	0	0	0	0	0	1	0
Encyclia bicalhoi	E_bic	1	1	0	0	0	1	0
Encyclia bocourtii	E_boc	0	0	0	0	0	0	0
Encyclia bracteata	E_bra	0	0	0	0	0	0	0
Encyclia bractescens	E_brt	0	0	0	0	0	1	0
Encyclia candollei	E_can	0	0	1	1	0	1	0
Encyclia ceratistes	E_cer	1	0	0	0	0	1	0
Encyclia chapadensis	E_cha	0	0	0	0	0	1	0
Encyclia cordigera	E_cor	0	0	0	0	0	1	1
Encyclia dichroma	E_dic	0	0	0	0	0	0	1
Encyclia diota	E_dio	0	0	0	0	0	0	0
Encyclia diurna	E_diu	1	0	0	0	0	0	0
Encyclia fehlingii	E_feh	1	0	0	0	0	0	0
Encyclia fucata	E_fuc	1	0	0	0	0	1	1
Encyclia garciae-esquivelii	E_gar	1	1	0	0	0	1	0
Encyclia guatemalensis	E_gua	1	1	0	1	0	1	0
Encyclia hanburyi	E_han	0	0	0	0	0	1	1
Encyclia howardii	E_how	0	0	0	0	0	0	0
Encyclia huertae	E_hue	0	0	0	0	1	1	0

Encyclia inaguensis	E_ina	0	0	0	0	0	1	0
Encyclia incumbens	E_inc	0	0	0	0	0	1	0
Encyclia ivonae	E_ivo	0	0	0	1	0	1	0
Encyclia meliosma	E_mel	0	0	0	0	1	1	0
Encyclia microtos	E_mic	0	0	0	0	0	1	0
Encyclia moebusii	E_moe	1	0	0	1	0	1	0
Encyclia mooreana	E_moo	1	0	0	0	0	0	0
Encyclia naranjapatensis	E_nar	0	0	0	0	0	1	0
Encyclia nematocaulon	E_nem	0	1	1	0	0	1	0
Encyclia odoratissima	E_odo	0	0	1	0	0	1	0
Encyclia oncidioides	E_onc	0	0	0	0	0	1	1
Encyclia osmatha	E_osm	0	0	0	0	0	1	0
Encyclia parviflora	E_par	1	0	0	0	0	1	0
Encyclia patens	E_pat	0	0	1	0	0	1	0
Encyclia pauciflora	E_pau	0	0	0	0	0	1	0
Encyclia phoenicea	E_pho	0	0	0	0	0	1	0
Encyclia plicata	E_pli	0	0	0	0	0	0	0
Encyclia pollardiana	E_pol	0	0	0	1	0	0	0
Encyclia profusa	E_pro	0	0	0	0	0	1	0
Encyclia pyriformis	E_pyr	0	0	0	0	0	1	0
Encyclia rufa	E_ruf	0	0	0	0	0	0	0
Encyclia seidelii	E_sei	0	0	0	1	1	1	0
Encyclia selligera	E_sel	0	0	0	0	0	1	0
Encyclia spiritusanctensis	E_spi	0	0	0	0	0	1	0
Encyclia tampensis	E_tam	0	0	0	0	0	1	0
Encyclia trachycarpa	E_tra	1	1	0	0	1	1	0
Encyclia trachychila	E_trh	1	1	0	0	1	1	1

Table S5 Average values for measured floral characters. For detailed description of traits, see Table 2 in the main text and Fig. S11 in Additional file 2

Taxon	Code	PL1	PL2	PL3	PL4	DS1	DS2	DS3	DS4	LS1	LS2	LS3	LS4	LIP1	LIP2	LIP3	LIP4	LIP5	LIP6	LIP7	GYN 1	GYN 2	GYN 3
E. acutifolia	E_acu	1,5	5,0	2,0	18,5	1,5	6,0	3,0	19,5	1,5	5,8	3,0	19,5	4,5	2,0	2,0	7,5	5,0	10,0	16,0	3,5	2,5	10,0
E. altissima	E_alt	1,5	7,0	2,0	24,5	1,5	7,0	3,0	25,0	1,5	7,0	3,5	24,5	8,0	3,0	3,5	17,0	7,0	13,5	24,5	5,0	3,0	10,0
E. ambigua	E_amb	2,0	7,5	2,0	22,0	2,0	7,0	2,5	24,5	2,0	7,0	4,0	23,0	10,0	3,0	4,5	13,5	5,5	10,5	21,0	5,0	3,0	12,0
E. asperula	E_asp	1,5	2,8	1,0	12,5	1,5	4,3	2,5	16,5	1,5	4,0	2,8	14,5	3,5	1,5	1,8	6,3	2,3	5,0	13,0	3,5	2,0	6,5
E. belizensis	E_bel	1,5	6,0	1,8	19,0	2,0	6,5	3,0	20,0	2,0	6,5	3,0	18,0	5,5	2,0	3,5	11,0	4,0	10,5	16,0	5,0	3,0	10,0
E. bracteata	E_bra	1,5	4,0	1,5	14,5	1,5	5,0	3,0	15,5	1,5	5,0	3,3	15,5	3,5	2,0	3,0	9,5	2,0	5,5	16,0	4,0	2,5	8,5
E. candollei	E_can	3,5	6,3	2,0	17,0	1,5	5,5	2,0	19,0	1,5	5,5	3,0	17,0	5,0	2,0	4,0	7,0	5,0	11,0	14,5	3,0	2,0	7,0
E. ceratistes	E_cer	1,3	4,4	1,1	17,3	1,3	4,5	2,5	17,8	1,0	4,3	2,8	18,0	4,5	2,0	2,1	6,8	3,3	7,0	16,5	3,3	2,0	8,5
E. diota	E_dio	3,5	10,0	2,0	15,5	3,0	7,5	4,0	16,5	2,5	7,5	4,0	17,0	6,5	2,5	2,5	7,5	5,0	10,0	14,0	4,0	3,0	10,0
E. diurna	E_diu	2,5	6,5	1,8	20,0	2,5	6,0	3,0	21,0	2,5	6,0	3,0	20,5	6,5	3,0	3,0	11,0	4,0	9,5	18,0	4,0	3,0	10,0
E. garciae-esquivelii	E_gar	1,7	4,3	1,3	18,0	2,0	5,0	2,8	17,7	2,0	5,0	3,0	17,0	5,7	2,7	2,5	8,3	4,3	9,0	15,7	3,7	2,3	9,7
E. hanburyi	E_han	2,3	8,5	1,8	21,0	2,0	8,0	3,3	22,3	1,7	7,7	4,0	22,0	7,0	3,0	3,0	15,3	4,3	13,0	22,3	3,7	2,7	11,0
E. moebusii	E_moe	1,7	4,7	2,2	18,3	1,7	5,3	3,0	19,3	1,7	6,0	3,7	19,7	7,7	2,3	3,3	16,0	5,7	10,0	18,7	5,0	3,3	12,0
E. mooreana	E_moo	2,0	4,0	1,3	12,0	2,0	4,0	3,0	13,5	2,0	4,5	3,0	13,5	3,0	2,0	2,0	8,0	3,5	7,0	12,5	2,5	1,8	6,0
E. parviflora	E_par	2,3	6,3	1,7	20,7	2,3	7,0	3,0	22,7	2,7	6,7	3,3	21,7	5,7	2,3	3,0	11,0	4,7	11,3	16,3	4,7	2,7	9,7
E. patens	E_pat	2,0	5,7	2,0	18,0	1,7	5,0	3,0	16,3	1,7	5,7	3,2	16,0	4,7	2,3	2,5	8,3	3,0	8,3	14,7	4,0	2,0	7,7
E. phoenicea	E_pho	1,7	8,3	2,3	22,7	1,7	9,3	4,0	24,0	1,3	9,3	4,0	24,0	9,0	3,0	4,7	21,7	7,0	14,3	26,3	6,7	3,7	13,3
E. plicata	E_pli	2,0	11,3	2,2	23,3	3,0	9,3	3,7	25,0	2,3	9,0	4,3	23,7	10,0	2,7	4,3	14,3	5,7	15,3	20,0	6,3	3,3	12,7
E. pyriformis	E_pyr	2,0	7,5	2,5	23,0	2,5	8,8	4,0	23,5	2,0	8,0	4,5	23,5	7,5	4,0	5,0	19,5	6,5	14,5	26,5	7,5	4,0	13,0
E. rufa	E_ruf	1,0	4,0	1,8	17,0	2,0	5,5	2,0	18,0	1,5	5,0	2,5	17,0	4,0	2,0	3,0	12,0	3,5	8,0	17,5	3,5	2,5	6,5
E. spiritusanctensis	E_spi	2,5	11,5	2,5	21,0	3,0	7,5	4,5	21,5	3,0	7,8	4,5	21,0	6,0	4,0	3,0	14,5	5,0	11,5	23,0	5,0	3,0	11,0
E. tampensis	E_tam	2,3	4,7	1,8	17,7	2,3	5,7	2,7	18,3	2,0	6,3	3,5	19,0	4,7	2,0	2,5	7,7	5,7	8,0	15,3	4,3	2,7	10,0

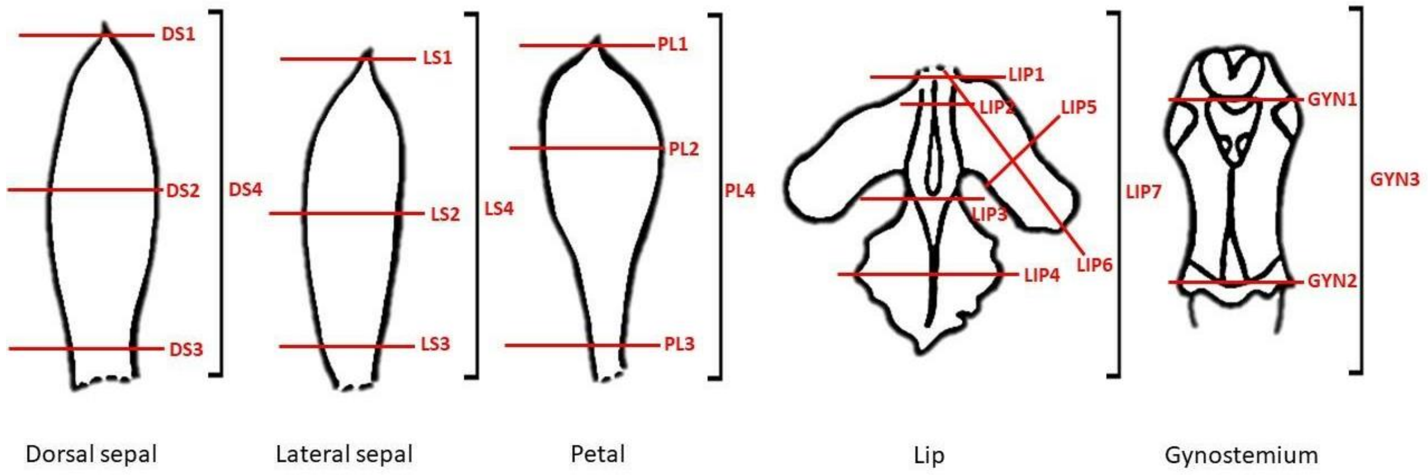

Fig. S11 Graphical presentation of the measured floral characters used in the multivariate analyses (see Table 2 in the main text for a detailed description of traits)
