# Supplementary material for: Characters evolution of Encyclia (Laeliinae-Orchidaceae) reveals a complex pattern not phylogenetically determined: insights from macro- and micromorphology
Source: BMC Plant Biol. 2023 Dec 20;23:661. doi: 10.1186/s12870-023-04664-3 (PMC10731901; doi:10.1186/s12870-023-04664-3)
Supplement: Supplementary file 3 — Additional file 3. Detailed descriptions of labellar micromorphology. [file 12870_2023_4664_MOESM3_ESM.pdf]

ADDITIONAL FILE 3

Detailed descriptions of labellar micromorphology

Encyclia acutifolia

Lip surface mostly papillose. Base of the lip covered with palisade like cells. Upper parts of lateral lobes papillose, with obpyriform, striate papillae. Lower parts glabrous with striate cuticle. Callus glabrous, with striate cuticle. Middle lobe covered with obpyriform papillae with striate cuticle.

Encyclia adenocarpos

Lip surface mostly papillose. Base of the lip ribbed. Lateral lobes covered with striate cuticle, loosely scattered conical papillae and residues of secretion. Callus papillose with striate cuticle, densely covered with conical papillae and remnants of secretion. Middle lobe covered densely with conical to short villiform papillae with striate surface, occurring on both inner and outer surface.

Encyclia adenocaulon

Lip surface papillose. Basal of the lip covered with conical papillae with gently striate surface, some of them have slightly swollen tips. Lateral lobes covered with short, conical papillae with striate cuticle, which are getting longer towards the callus. Callus densely covered with conical papillae and with visible residues of secretion. Middle lobe covered with conical papillae with striate cuticle, distal parts rugose to verrucose. Several stoma and residues of secretion present.

Encyclia aenicta

Lip surface papillose. Lip base covered with scattered conical papillae at different stages of development. Lateral lobes rugose, with striate cuticle. Callus papillose, covered with villiform papillae with striate surface and visible residues of secretion. Middle lobe glabrous, with striate cuticle.

Encyclia alata

Lip surface glabrous. Lip base with striate cuticle. Lateral lobes with striate cuticle and elevations of cells with scattered stoma. Callus glabrous, with palisade-like cells and striate cuticle. Middle lobe strongly verrucose, with striate cuticle and cell elevations with stomata.

Encyclia altissima

Lip surface rather papillose. Base of the lip and lateral lobes covered with obpyriform papillae with striate cuticle. Callus ridged, with striate cuticle and visible residues of secretion. Middle lobe rugose to verrucose, covered with obpyriform papillae with striate cuticle. Several stomata located on the top of cells aggregations present, visible residues of viscid secretion.

Encyclia amanda

Lip surface rather glabrous. Lip base covered by ribbed cuticle. Lateral lobes glabrous, with slightly striate cuticle and remnants of secretion. Callus papillose with striate conical papillae in the central part, villiform on the margins with residues of secretion, and glabrous, strongly striate surface (needle-like structure) on the remaining part. Middle lobe glabrous with striate cuticle.

Encyclia ambigua

Lip surface papillose. Lateral lobes densely covered with obpyriform papillae with striate cuticle. Callus papillose with striate obpyriform papillae in the central part and single to 2-celled trichomes on the margins with residues of secretion. Middle lobe strongly verrucose, densely covered by conical to obpyriform papillae with striate cuticle, visible residues of secreted material and cell elevations with stoma.

Encyclia andrichii

Lip surface rather papillose. Lip base covered with loosely arranged obpyriform papillae and striate cuticle. Lateral lobes glabrous with striate cuticle. Callus covered with conical to obpyriform papillae and strongly striate cuticle with a significant amount of secretion. Middle lobe rugose to verrucose in the central part, with striate cuticle.

Encyclia aspera

Lip surface glabrous. Lateral lobes glabrous with slightly striate cuticle. Callus glabrous with striate cuticle and slightly pointed cells. Middle lobe rugose to strongly verrucose, with smooth cuticle.

Encyclia asperula

Lip surface glabrous. Lip base with palisade-like cells and residues of secretion. Lateral lobes glabrous with striate cuticle. Callus glabrous with striate cuticle and a significant amount of probably wax-like secretion. Middle lobe rugose to strongly verrucose in the central part, covered with striate cuticle.

Encyclia belizensis

Lip surface papillose. Base of the lip covered with conical papillae with striate cuticle and residues of secretion, some of them have swollen tips. Lateral lobes densely covered with obpyriform to conical papillae with striate cuticle and residues of secretion. Callus densely covered with obpyriform papillae with striate cuticle and significant amount of secretion. Middle lobe rugose to verrucose, densely covered with obpyriform papillae, main veins glabrous, stomata present at the elevations of cells.

Encyclia bicalhoi

Lip surface mostly glabrous. Base of the lip ridged, covered with loosely scattered conical papillae with remnants of secreted material. Lateral lobes glabrous, rugose to verrucose, with striate cuticle. Callus glabrous, with striate cuticle. Middle lobe strongly verrucose, covered with striate cuticle.

Encyclia bocourtii

Lip surface glabrous. Lateral lobes rugose to verrucose, glabrous, with striate cuticle. Callus glabrous, with striate cuticle and significant amount of probably wax-like secretion. Middle lobe rugose to verrucose, glabrous with striate cuticle.

Encyclia bracteata

Lip mostly glabrous. Lateral lobes glabrous with palisade like cells with striate cuticle. Upper part of the callus strongly papillose with villiform papillae and multicellular trichomes with striate cuticle. Central part covered with palisade like cells with intensely striate cuticle and visible layer of viscid, probably wax-like material. Middle lobe glabrous, covered in cells with striate cuticle and with several stoma present.

Encyclia bractescens

Lip surface papillose. Lateral lobes glabrous, covered with striate cuticle. Callus in its upper part covered with numerous single celled trichomes and residues of secretion, and in lower covered densely in villiform and obpyriform papillae. Middle lobe rugose to verrucose, covered with obpyriform papillae on both inner and outer surfaces. Papillae on the outer side of the lip with more less striate surface.

Encyclia candollei

Lip surface mostly glabrous. Lateral lobes glabrous with striate cuticle. Callus densely papillose, covered with conical to villiform papillae with striate surface and secretion residues. Middle lobe strongly verrucose, covered with grooved cuticle.

Encyclia ceratistes

Lip surface glabrous. Lip base striate, with palisade-like cells. Lateral lobes glabrous, with slightly striate cuticle. Callus glabrous with strongly striate cuticle and residues of secretion. Middle lobe glabrous with striate cuticle and significant amount of secretion.

Encyclia chapadensis

Lip surface glabrous. Lip base striate. Lateral lobes glabrous with striate cuticle. Callus glabrous with striate cuticle. Middle lobe glabrous with striate cuticle.

Encyclia cordigera

Lip surface papillose. Base of the lip striate, with conical papillae and short presumably secretory trichomes, both with striate cuticle and loosely scattered, visible residues of secretion. Lateral lobes densely papillose with conical papillae with striate cuticle and residues of secretion, glabrous in the distal part. Callus papillose with conical to villiform papillae with striate cuticle, densely covered with secreted material, probably wax-like. Middle lobe ribbed, generally glabrous, conical papillae present on the surface between the ribs. Distal part of the middle lobe covered with obpyriform papillae.

Encyclia dichroma

Lip surface papillose. Base of the lip ribbed, with scattered conical papillae with striate cuticle. Lateral lobes densely covered with conical papillae with striate cuticle, shorter and better organized on the distal part. Callus densely covered with conical papillae with striate cuticle and viscid secretions in places. Middle lobe densely covered with conical to villiform papillae with striate cuticle.

Encyclia diota

Lip surface mostly papillose. Base of the lip covered with few scattered conical papillae. Upper parts of lateral lobes densely covered with conical papillae with striate surface. Lower parts glabrous with grooved cells. Callus densely covered with villiform papillae and significant amount of probably wax like secretion. Middle lobe rugose to verrucose, glabrous, covered with striate cuticle.

Encyclia diurna

Lip surface mostly glabrous. Lip base with striate cuticle. Upper parts of lateral lobes covered with obpyriform papillae with striate surface. Lower glabrous with striate cuticle. Callus glabrous with strongly striate cuticle. Middle lobe rugose to verrucose, covered with striate cuticle. Single stomata present.

Encyclia fehlingii

Lip surface glabrous. Lip base covered with palisade-like cells. Lateral lobes glabrous with striate cuticle. Callus rugose, glabrous, with striate cuticle and significant residues of secretion. Middle lobe rugose on the margins to strongly verrucose on the central part, covered with striate cuticle.

Encyclia fucata

Lip surface mostly papillose with different type of papilla. Base of the lip covered with obpyriform papillae and visible residues of secretion. Lateral lobes with scattered cuticular blisters, covered in obpyriform papillae with striate surface and remnants of secretion. Callus densely papillose, covered with typical conical and conical with swallowed top cells papillae, both with striate surface and secretion residues. Middle lobe rugose to verrucose, covered with obpyriform papillae with striate surface.

Encyclia garciae-esquivelii

Lip surface mostly glabrous. Lip base in central part glabrous, with few papillae in early stage of development on the sides, with striate cuticle. Lateral lobes glabrous, with smooth cuticle. Callus generally smooth, with striate cuticle and residues of probably wax-like secretion central part, on sides covered with obpyriform papillae. Middle lobe glabrous, with striate cuticle.

Encyclia guatemalensis

Lip surface glabrous. Lip base ribbed with striate cuticle and scattered conical papillae, some fused together in three. Lateral lobes glabrous, some cells with slightly striate cuticle. Callus glabrous, epidermal cells with striate cuticle. Middle lobe rugose to verrucose, glabrous, epidermal cells with slightly striate cuticle.

Encyclia hamburyi

Lip surface papillose. Base of the lip striate with scattered conical to short villiform papillae. Lateral lobes densely covered with rounded conical papillae with striate cuticle in proximal part, glabrous with slightly striate cuticle in distal. Callus densely covered with conical to villiform papillae with striate cuticle and with significant amount of secreted material, and single celled trichomes. Middle lobe ribbed, with most of the surface covered with conical papillae with striate cuticle and residues of secretion.

Encyclia howardii

Lip surface glabrous. Lip base with striate cuticle and scattered obpyriform papillae. Lateral lobes glabrous with striate cuticle. Callus with striate cuticle, significant amount of dense, probably wax-like secretion and structures resembling moniliform trichomes in its central part. Middle lobe glabrous with striate cuticle.

Encyclia huertae

Lip surface mostly glabrous. Base of the lip covered with elongated conical papillae, some of them with few thickenings. Lateral lobes glabrous, with striate cuticle. Callus papillose in upper part, covered with conical papillae (sometimes with swallowed tips) and significant amount of probably secretion, striate cuticle. Middle lobe rugose to slightly verrucose, glabrous with striate cuticle.

Encyclia inaguensis

Lip surface glabrous. Lateral lobes glabrous with striate cuticle. Callus glabrous, with striate cuticle and significant amount of secretion. Middle lobe strongly verrucose, with striate cuticle and few stoma present.

Encyclia incumbens

Lip surface papillose. Lateral lobes papillose with obpyriform to conical papillae with striate surface. Callus densely papillose, covered with conical to villiform papillae with striate surfaces and scattered two-celled trichomes. Middle lobes strongly verrucose and the whole surface densely covered with conical to obpyriform papillae with striate surfaces.

Encyclia ivonae

Lip surface rather papillose. Base of the lip densely covered with conical papillae with striate cuticle. Lateral lobes glabrous with striate cuticle. Callus glabrous, with striate cuticle and significant amount of secreted material. Middle lobe rugose to slightly verrucose, covered with obpyriform papillae with striate cuticle, glabrous towards the margins.

Encyclia meliosma

Lip surface papillose. Lip base covered with conical to villiform papillae and significant amount of secretion. Lateral lobes covered with striate cuticle. Upper parts with loosely arranged conical papillae, center and lower parts glabrous, rugose. Callus papillose, densely covered with villiform papillae and secretion. Middle lobe glabrous, rugose to verrucose with margins covered by palisade-like, striate cells.

Encyclia microtes

Lip surface glabrous. Lateral lobes rugose, glabrous, epidermal cells with slightly striate cuticle. Callus verrucose, glabrous with striate cuticle in central part, on sides present aggregations of trichomes covered in secretion. Middle lobe verrucose, with striate cuticle, several stomata and secretion.

Encyclia moebusii

Lip surface rather glabrous. Lateral lobes densely covered with obpyriform papillae, slightly striate. Callus glabrous with strongly striated cuticle. Middle lobe glabrous with striate cuticle.

Encyclia mooreana

Lip surface glabrous. Lateral lobes glabrous with striate cuticle. Callus mostly glabrous with significant amount of probably wax like secretion, conical to villiform papillae present on the sides in upper part. Middle lobe glabrous with striate cuticle.

Encyclia naranjapatensis

Lip surface papillose. Base of the lip glabrous with striate cuticle in the center, and on the sides with rounded papillae arranged in rows, visible residues of secretion. Lateral lobes covered in some places with obpyriform papillae. Callus glabrous, with striate cuticle, on sides with villiform papillae covered in residues of secretion. Middle lobe glabrous, with striate cuticle.

Encyclia nematocaulon

Lip surface papillose. Base of the lip striated, with few scattered obpyriform to conical papillae. Lateral lobes glabrous with rather smooth cuticle. Callus densely papillose, covered with villiform papillae with swellings and significant amount of probably wax like secretion. Middle lobe papillose, covered with obpyriform to conical and villiform papillae with swellings, with slightly striate cuticle.

Encyclia oblongata

Lip surface rather glabrous. Base of the lip with small, obpyriform papillae with striate cuticle. Lateral lobes glabrous, with striate cuticle. Callus glabrous with striate surface. Middle lobe slightly rugose, glabrous, with striate cuticle.

Encyclia odoratissima

Lip surface rather glabrous. Lip base covered with small, obpyriform to conical papillae with striate cuticle. Lateral lobes glabrous with slightly striate cuticle and significant number of crystal-like structures Callus glabrous, with palisade-like cells and striate cuticle, covered with significant amount of secretion and crystal-like structures. Middle lobe glabrous with striate cuticle and significant amount of crystal-like structures.

Encyclia oncidioides

Lip surface glabrous. Base of the lip strongly striated, covered with significant amount of secretion residues. Lateral lobes slightly rugose, glabrous, with smooth cuticle. Callus glabrous, with striate cuticle and residues of secretion. Middle lobe glabrous with striate cuticle.

Encyclia osmantha

Lip surface glabrous. Base of the lip glabrous with striate cuticle and visible layer of viscid secretion, probably wax-like. Lateral lobes glabrous with striate cuticle. Callus glabrous with striate cuticle and visible layer of viscid secretion, probably wax-like and undetermined grains scattered in central part. Middle lobe rugose, with palisade-like cells and striate cuticle, several stoma present. Surface covered in grains of unknown origin, probably secretion.

Encyclia papillosa

Lip surface papillose. Lip base covered with scarce conical papillae. Lateral lobes covered densely with striate conical papillae at different stages of development. Callus with loosely arranged rounded to conical papillae and striate cuticle. Middle lobe densely covered with obpyriform to conical papillae with striate cuticle.

Encyclia parviflora

Lip surface glabrous. Lateral lobes, rugose to slightly verrucose, ribbed, with striate cuticle and residues of secreted material. Generally glabrous, distal part papillose with obpyriform papillae. Callus glabrous with striate cuticle, visible layer of viscid substance – probably wax-like secretion. Middle lobe strongly verrucose, ribbed, glabrous with striate cuticle, several stoma present.

Encyclia patens

Lip surface glabrous. Base of the lip covered in palisade-like, striate cells. Lateral lobes glabrous with striate cuticle. Callus glabrous with strongly striate surface and significant amount of probably wax-like secretion. Middle lobe glabrous with striate cuticle, residues od secretion and stoma present.

Encyclia pauciflora

Lip surface glabrous. Lateral lobes glabrous, with palisade-like cells and slightly striate cuticle. Callus glabrous with some villiform short papillae on sides and outer surface of the lip. Middle lobe rugose, with striate cuticle.

Encyclia phoenicea

Lip surface rather glabrous. Lateral lobes and callus glabrous with striate cuticle. Middle lobe slightly verrucose, densely covered with obpyriform papillae with striate cuticle and residues of secretion. Single stomata present.

Encyclia plicata

Lip surface mostly glabrous. Base of the lip densely covered with conical papilla with striate cuticle and somewhat swollen apices. Lateral lobes glabrous with striate cuticle and scattered verrucae. Callus glabrous with striate cuticle and secretion residues. On distal part present some remnants of probably wax-like substances. Middle lobe rugose to slightly verrucose, with striate cuticle.

Encyclia pollardiana

Lip surface glabrous. Lateral lobes glabrous with slightly striate cuticle. Callus densely papillose with conical to villiform papillae, and multicellular trichomes, both with striate cuticle. Middle lobe glabrous with striate cuticle, several stomata and residues of secreted material, palisade-like cells in upper part.

Encyclia powellii

Lip surface rather glabrous. Lip base striate. Lateral lobes glabrous with striate cuticle. Callus glabrous with striate cuticle. Middle lobe glabrous with striate cuticle.

Encyclia profusa

Lip surface glabrous, Lateral lobes glabrous with striate cuticle and significant amount of secretion residues. Callus glabrous, rugose, with striate cuticle and significant amount of secretion. Middle lobe glabrous, rugose, with striate cuticle and significant amount of secretion

Encyclia pyriformis

Lip surface glabrous. Base of the lip ribbed. Lateral lobes, callus, and middle lobe glabrous with striate cuticle.

Encyclia rufa

Lip surface glabrous. Lateral lobes glabrous with striate cuticle. Callus glabrous, covered with secretion. Middle lobe glabrous, rugose, in places with blisters and striate cuticle, single stoma present.

Encyclia seidelli

Lip surface glabrous. Lip base striate, with few villiform papillae near callus. Lateral lobes glabrous with striate cuticle. Callus glabrous with striate cuticle. Middle lobe verrucose, cowered with striate cuticle and obpyriform papillae at different stage of development.

Encyclia selligera

Lip surface mostly glabrous. Base of the lip covered with striate conical to villiform papillae. Lateral lobes glabrous with striate cuticle. Callus papillose, with loosely scattered striate rounded to conical papillae and significant amount of secretion. Middle lobe glabrous, with striate cuticle and single stoma.

Encyclia spiritusanctensis

Lip surface papillose. Base of the striate, covered with significant amount of secretion. Lateral lobes covered with loosely arranged conical papillae in early stage of development and striate cuticle. Callus glabrous with striate cuticle, densely covered with secretion. Middle lobe covered with conical papillae in rather early stage of development.

Encyclia tampensis

Lip surface glabrous. Lateral lobes and callus glabrous with striate cuticle. Callus glabrous. Middle lobe strongly verrucose, covered with very gently striate to smooth cuticle.

Encyclia trachycarpa

Lip surface papillose. Lateral lobes mostly glabrous, with some scattered conical papillae on early stage of development, cuticle striate. Callus densely papillose with conical papillae and residues of secretion. Middle lobe densely covered with obpyriform to conical papillae with striate cuticle and several stomata scattered.

Encyclia trachychila

Lip surface papillose. Lateral lobes rugose, mostly glabrous, with striate cuticle, covered in places with scattered conical papillae. Callus papillose, with conical papillae in early stage of development and significant amount of secretion. On its sides several longer, probably villiform papillae are present. Middle lobe strongly verrucose, glabrous with striate cuticle.

Encyclia virens

Lip surface papillose. Base of the lip striate with rows of conical papillae. Lateral lobes rugose, densely covered by obpyriform papillae in distal part, and conical nearby callus, both with striate cuticle. Callus

rather glabrous, with striate cuticle and residues of secretion. Middle lobe ribbed, strongly verrucose, covered with obpyriform papillae with striate cuticle.
