# Supplementary material for: Characters evolution of Encyclia (Laeliinae-Orchidaceae) reveals a complex pattern not phylogenetically determined: insights from macro- and micromorphology
Source: BMC Plant Biol. 2023 Dec 20;23:661. doi: 10.1186/s12870-023-04664-3 (PMC10731901; doi:10.1186/s12870-023-04664-3)
Supplement: Supplementary file 4 — Additional file 4. Flowers of Encyclia species investigated in the study. Fig. S12. Flowers of Encyclia species investigated in the study: A, B – E. acutifolia; C – E. adenocaula; D – E. alata; E – E. altissima; F – E. amanda; G – E. ambigua. Phot. M. Speckmaier. Fig. S13. Flowers of Encyclia species investigated in the study: A – E. andrichii; B – E. aspera; C – E. atrorubens; D – E. belizensis; E – E. bracteata; F – E. candollei; G – E. ceratistes; H – E. chapadensis. Phot. M. Speckmaier. Fig. S14. Flowers of Encyclia species investigated in the study: A – E. caximboensis; B – E. conchaechila; C – E. cordigera; D – E. cordigera fo. leucantha; E – E. cordigera var. rosea; F – E. correllii; G – E. cyperifolia. Phot. M. Speckmaier. Fig. S15. Flowers of Encyclia species investigated in the study: A – E. dichroma; B – E. dickinsoniana; C – E. diota; D – E. diurna; E – E. elegantula; F – E. fehlingii; G – E. flabellata; H – E. fowliei. Phot. M. Speckmaier. Fig. S16. Flowers of Encyclia species investigated in the study: A – E. fucata; B – E. garciaeesquivelii; C – E. granitica; D – E. halbingeriana; E – E. hanburyi; F – E. howardii; G – E. huertae; H – E. incumbens. Phot. M. Speckmaier. Fig. S17. Flowers of Encyclia species investigated in the study: A – E. ivonae; B – E. kennedyi (right) and E. adenocaula (left); C – E. leucantha; D – E. linearifolioides; E – E. megalantha; F – E. microbulbon; G – E. moebusii. Phot. M. Speckmaier. Fig. S18. Flowers of Encyclia species investigated in the study: A – E. mooreana; B – E. naranjapatensis; C – E. nematocaulon; D – E. oncidioides; E – E. osmantha; F – E. oxypetala; G – E. parviflora; H – E. patens. Phot. M. Speckmaier. Fig. S19. Flowers of Encyclia species investigated in the study: A – E. pauciflora; B – E. pflanzii; C – E. phoenica; D – E. plicata; E – E. pollardiana; F – E. powellii; G – E. profusa; H – E. randii. Phot. M. Speckmaier. Fig. S20. Flowers of Encyclia species investigated in the study: A – E. rzedowskiana; B [file 12870_2023_4664_MOESM4_ESM.pdf]

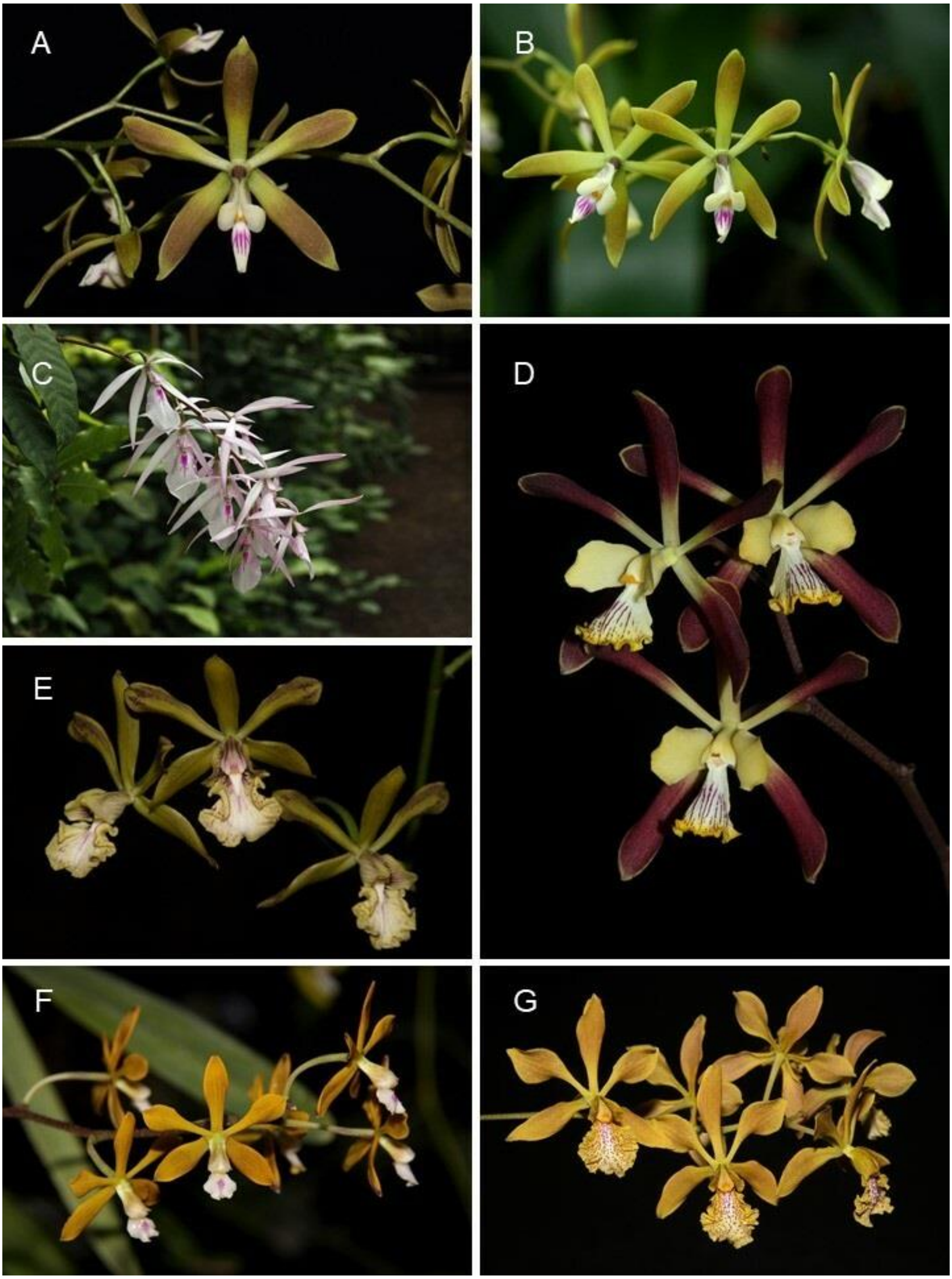

Fig. S12 Flowers of *Encyclia* species investigated in the study: **A**, **B** – *E. acutifolia*; **C** – *E. adenocaula*; **D** – *E. alata*; **E** – *E. altissima*; **F** – *E. amanda*; **G** – *E. ambigua*. Phot. M. Speckmaier

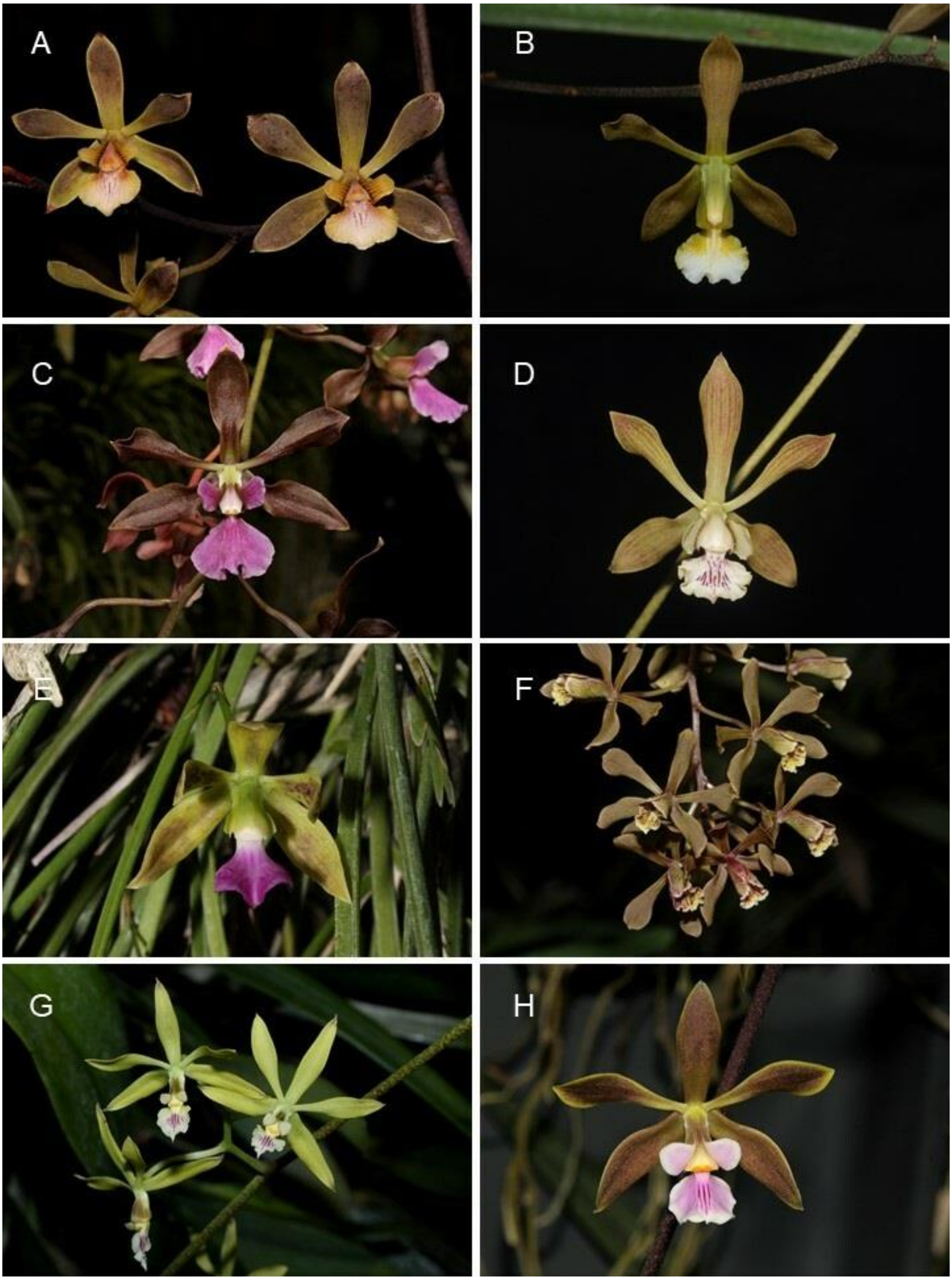

Fig. S13 Flowers of *Encyclia* species investigated in the study: **A** – *E. andrichii*; **B** – *E. aspera*; **C** – *E. atrorubens*; **D** – *E. belizensis*; **E** – *E. bracteata*; **F** – *E. candollei*; **G** – *E. ceratistes*; **H** – *E. chapadensis*. Phot. M. Speckmaier

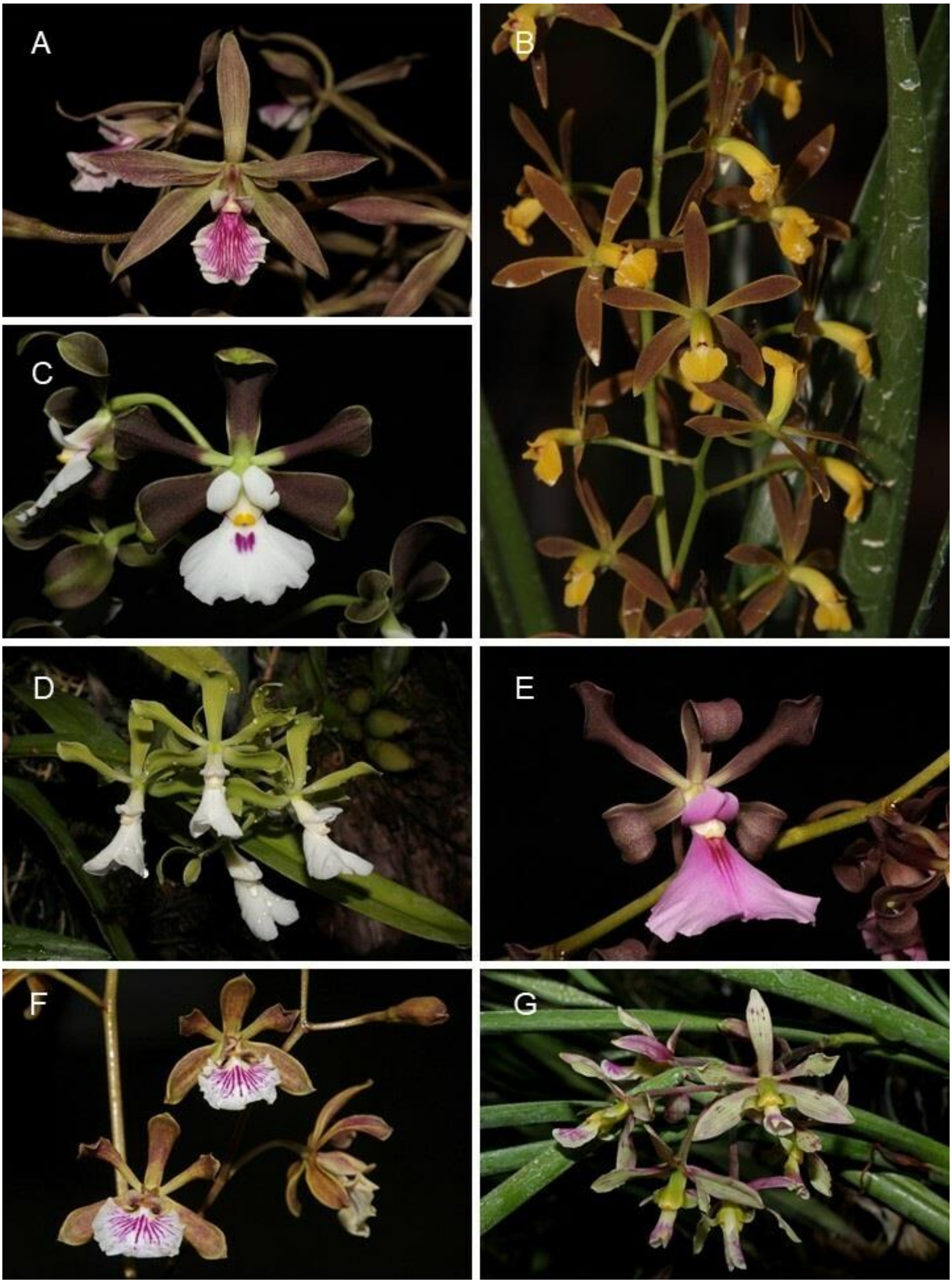

Fig. S14 Flowers of *Encyclia* species investigated in the study: **A** – *E. caximboensis*; **B** – *E. conchaechila*; **C** – *E. cordigera*; **D** – *E. cordigera f*o. *leucantha*; **E** – *E. cordigera* var. *rosea*; **F** – *E. correllii*; **G** – *E. cyperifolia*. Phot. M. Speckmaier

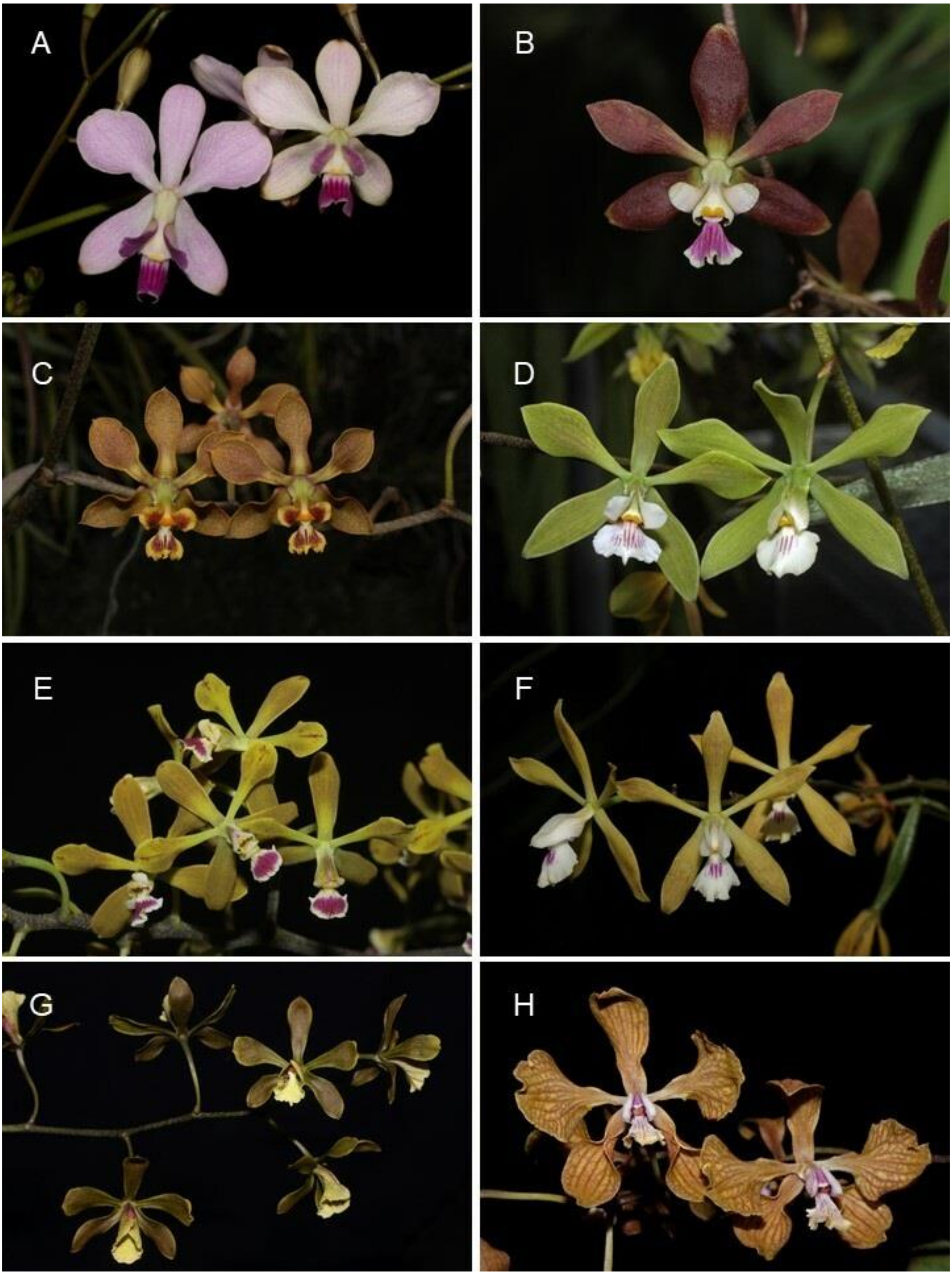

Fig. S15 Flowers of *Encyclia* species investigated in the study: **A** – *E. dichroma*; **B** – *E. dickinsoniana*; **C** – *E. diota*; **D** – *E. diurna*; **E** – *E. elegantula*; **F** – *E. fehlingii*; **G** – *E. flabellata*; **H** – *E. fowliei*. Phot. M. Speckmaier

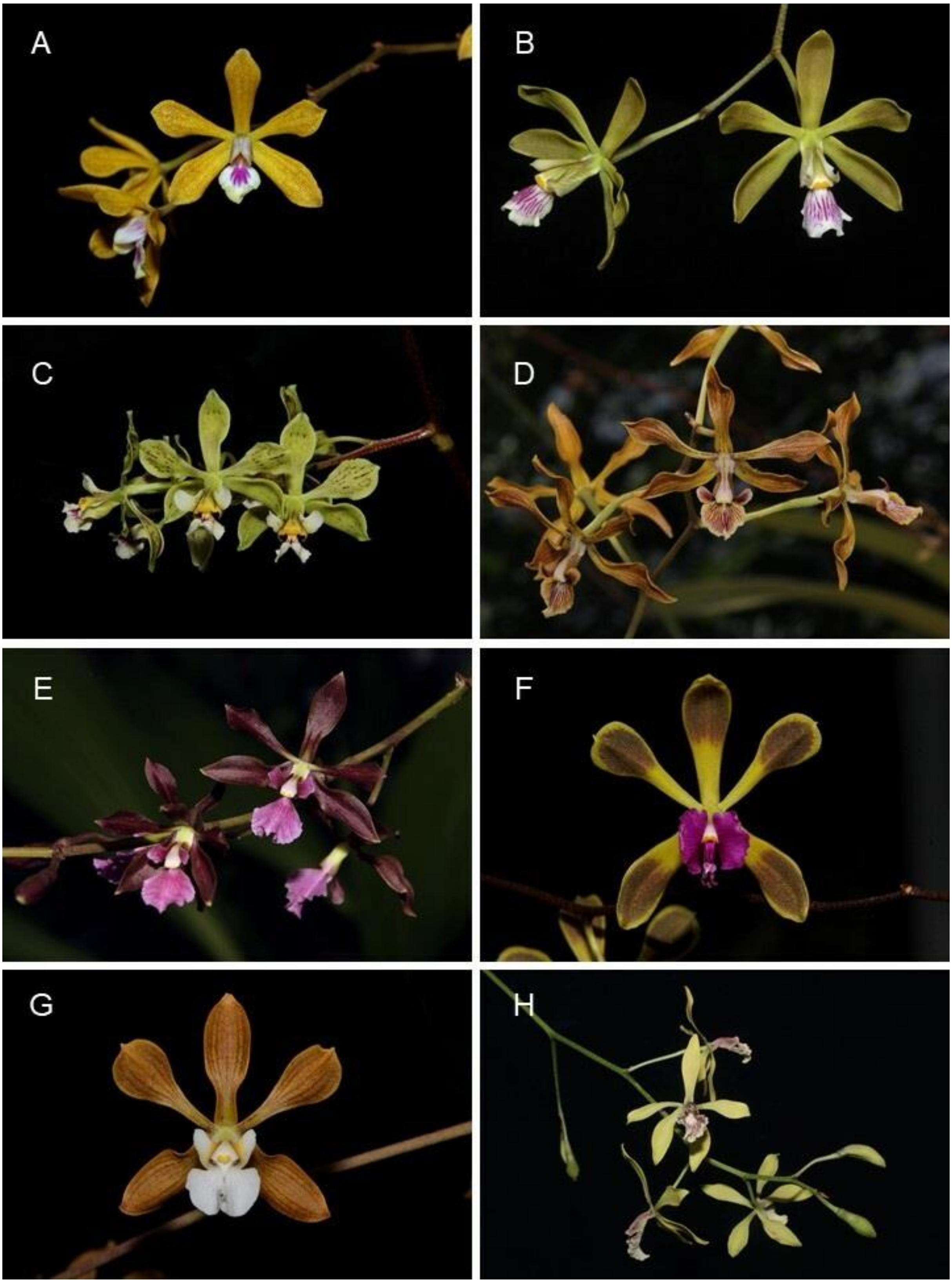

Fig. S16 Flowers of *Encyclia* species investigated in the study: **A** – *E. fucata*; **B** – *E. garciae-esquivelii*; **C** – *E. granitica*; **D** – *E. halbingeriana*; **E** – *E. hanburyi*; **F** – *E. howardii*; **G** – *E. huertae*; **H** – *E. incumbens.* Phot. M. Speckmaier

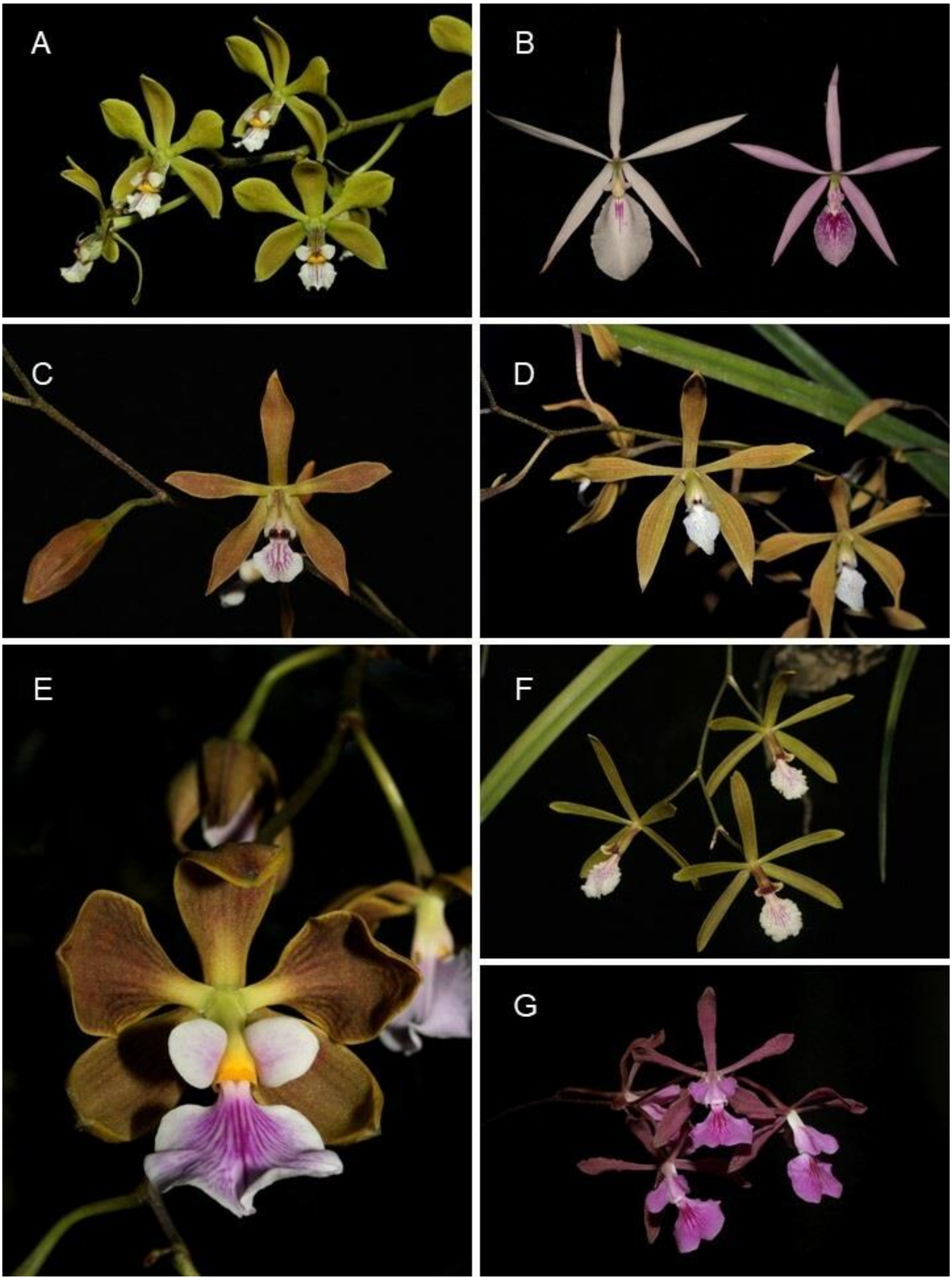

Fig. S17 Flowers of *Encyclia* species investigated in the study: **A** – *E. ivonae*; **B** – *E. kennedyi* (right) and *E. adenocaula* (left); **C** – *E. leucantha*; **D** – *E. linearifolioides*; **E** – *E. megalantha*; **F** – *E. microbulbon*; **G** – *E. moebusii*. Phot. M. Speckmaier

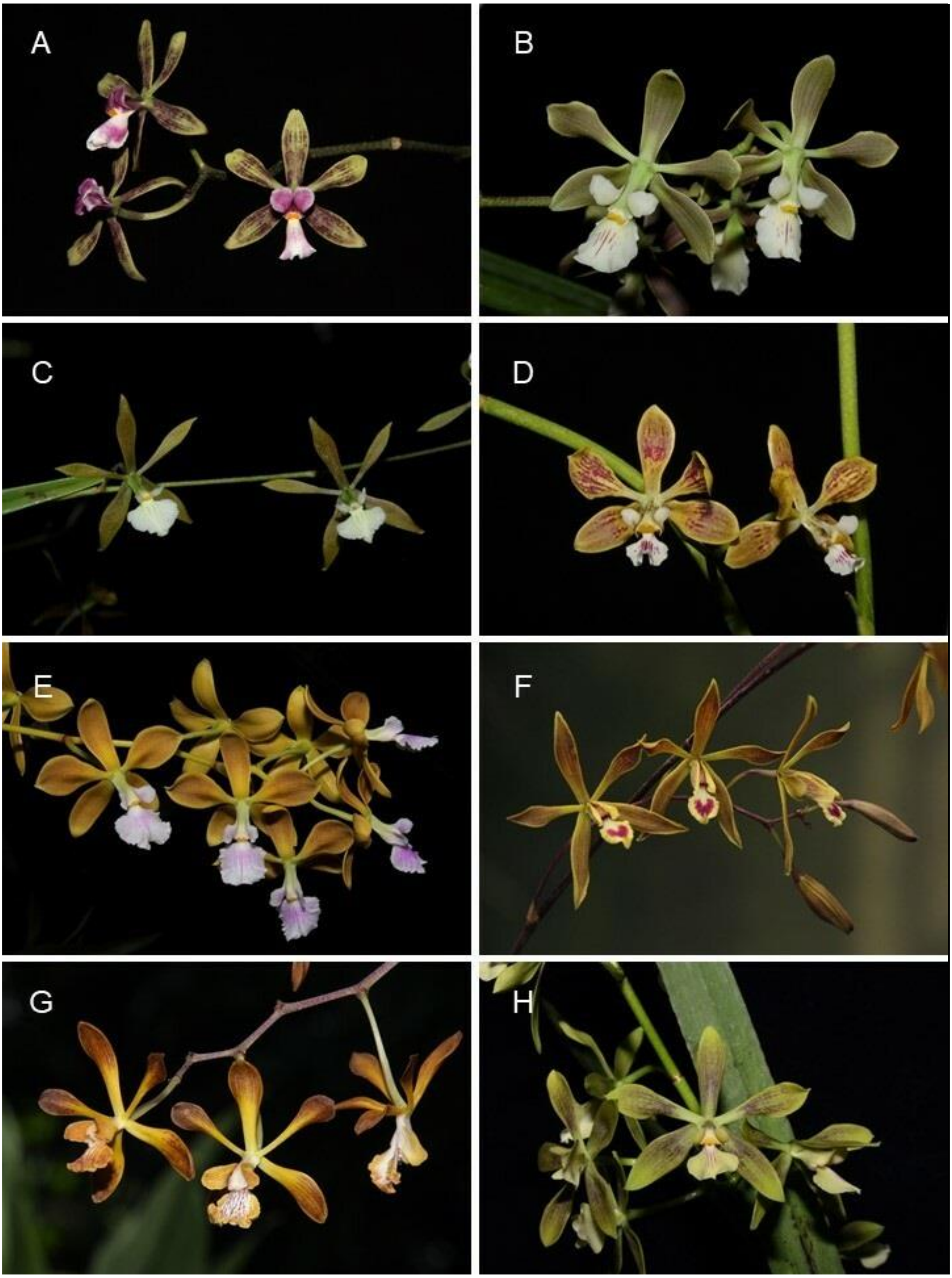

Fig. S18 Flowers of *Encyclia* species investigated in the study: **A** – *E. mooreana*; **B** – *E. naranjapatensis*; **C** – *E. nematocaulon*; **D** – *E. oncidioides*; **E** – *E. osmantha*; **F** – *E. oxypetala*; **G** – *E. parviflora*; **H** – *E. patens.* Phot. M. Speckmaier

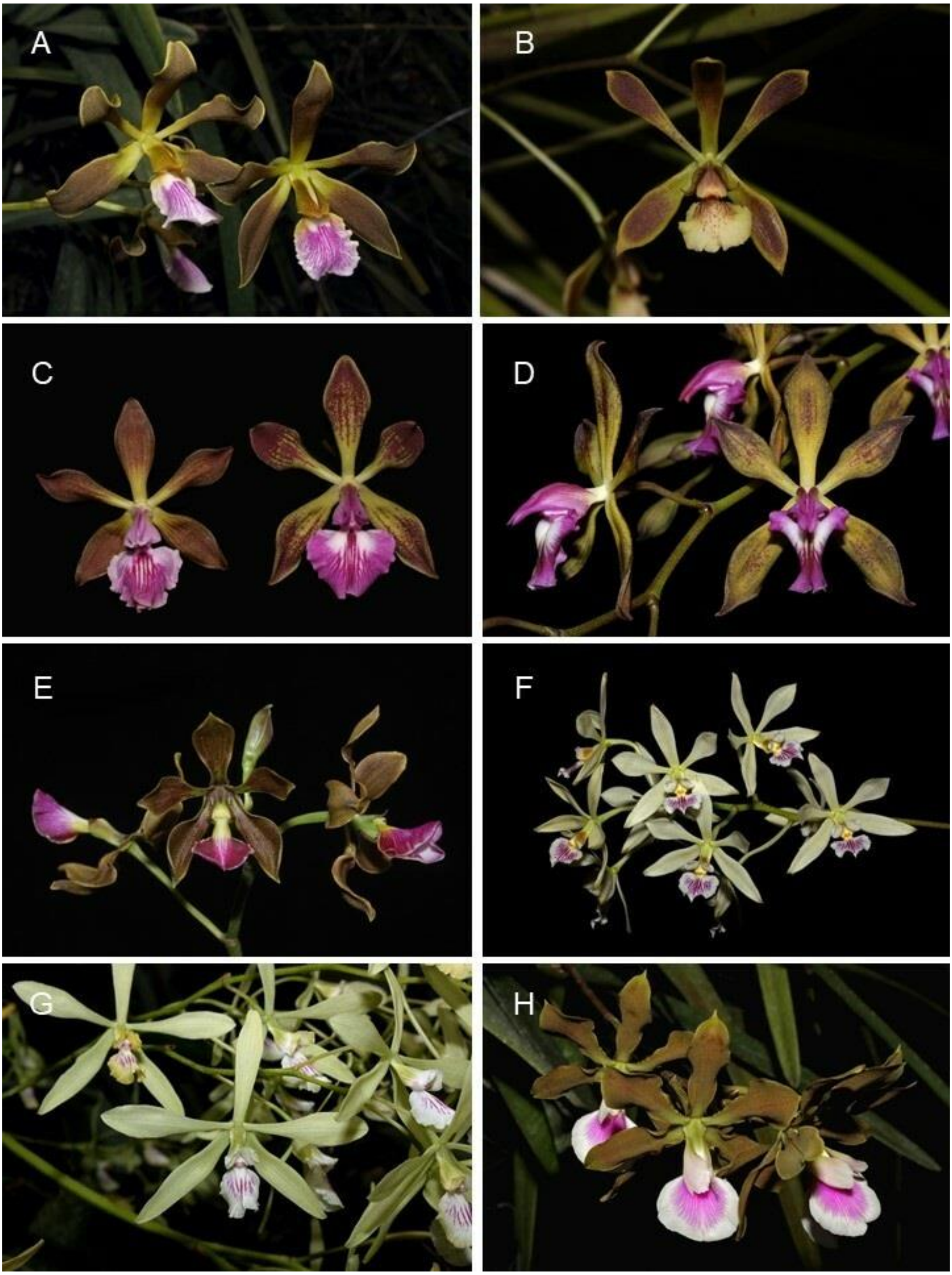

Fig. S19 Flowers of *Encyclia* species investigated in the study: **A** – *E. pauciflora*; **B** – *E. pflanzii*; **C** – *E. phoenica*; **D** – *E. plicata*; **E** – *E. pollardiana*; **F** – *E. powellii*; **G** – *E. profusa*; **H** – *E. randii*. Phot. M. Speckmaier

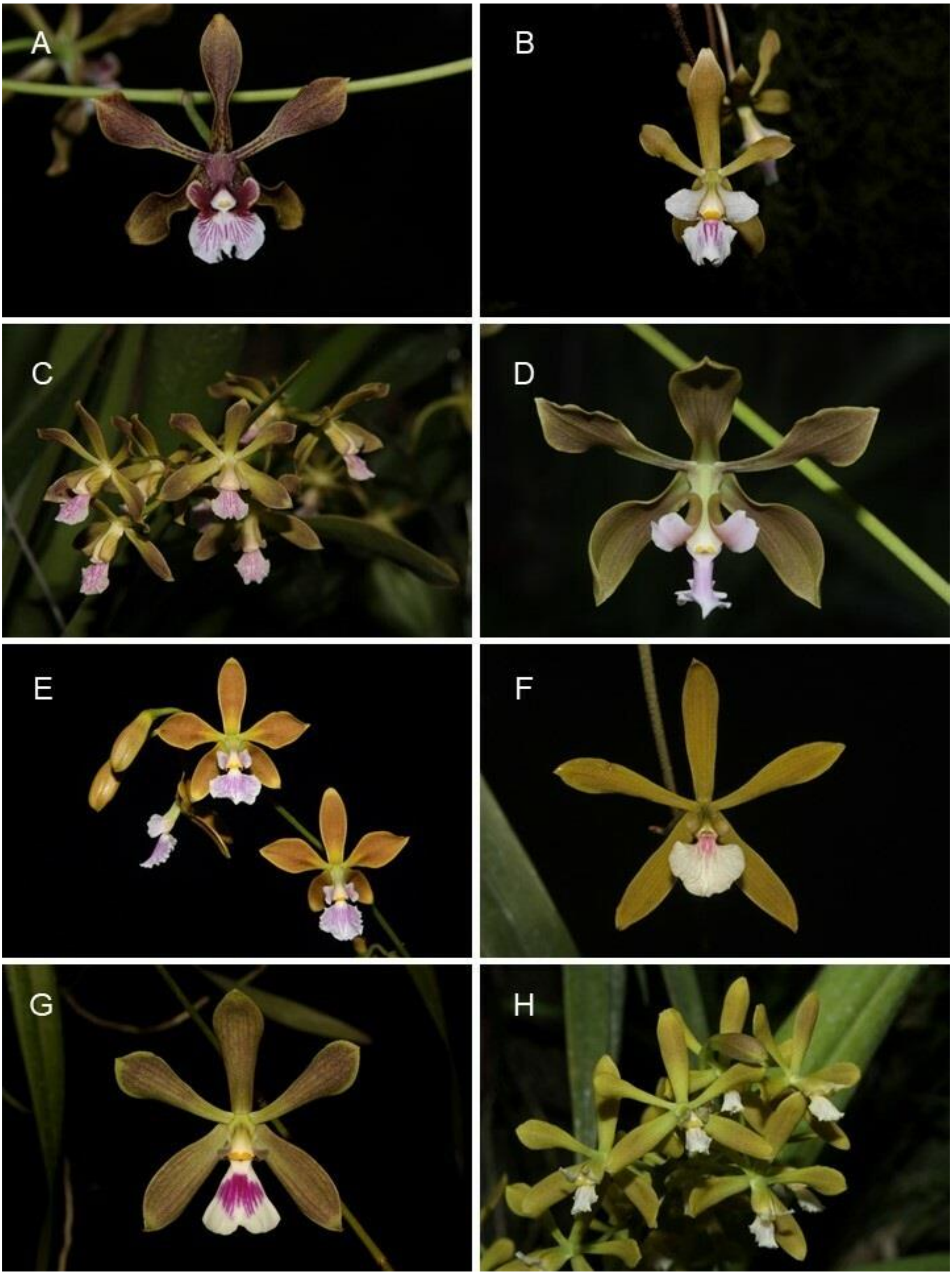

Fig. S20 Flowers of *Encyclia* species investigated in the study: **A** – *E. rzedowskiana*; **B** – *E. saltensis*; **C** – *E. seidelii*; **D** – *E. selligera*; **E** – *E. spiritusanctensis*; **F** – *E. stellata*; **G** – *E. tampensis*; **H** – *E. thienii*. Phot. M. Speckmaier

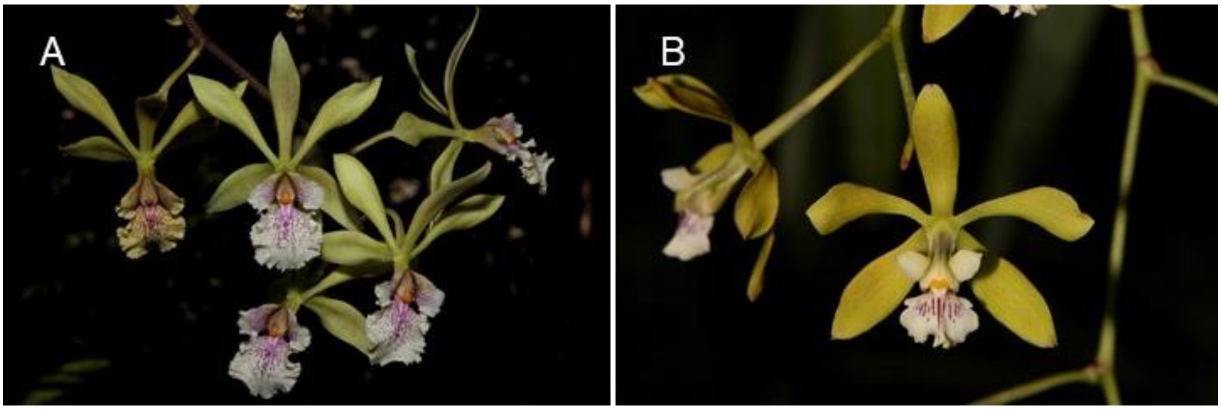

Fig. S21 Flowers of *Encyclia* species investigated in the study: **A** – *E. virens*; **B** – *E*. sp. Brazil. Phot. M. Speckmaier
