# Supplementary material for: Characters evolution of Encyclia (Laeliinae-Orchidaceae) reveals a complex pattern not phylogenetically determined: insights from macro- and micromorphology
Source: BMC Plant Biol. 2023 Dec 20;23:661. doi: 10.1186/s12870-023-04664-3 (PMC10731901; doi:10.1186/s12870-023-04664-3)
Supplement: Supplementary file 5 — Additional file 5. Voucher information for the taxa examined. Table S6. Voucher information for the taxa examined in the micromorphological analysis. Table S7. Voucher information for the taxa examined in the molecular analysis (fresh material). Table S8. Voucher information for the taxa examined in the multivariate analysis. [file 12870_2023_4664_MOESM5_ESM.pdf]

Table S6 Voucher information for the taxa examined in the micromorphological analysis

Species	Voucher	Source
Encyclia acutifolia	ORCH060621	Botanical Garden of the University of Vienna
Encyclia adenocarpa	35/2014	Peter Szebó
Encyclia aenicta	194/2015	Peter Szebó
Encyclia alata	97/2011	Peter Szebó
Encyclia amanda	ORCH121035	Botanical Garden of the University of Vienna
Encyclia andrichii	127b/2014	Peter Szebó
Encyclia aspera	72/2010	Peter Szebó
Encyclia asperula	130453	Botanical Garden of the University of Vienna
Encyclia bicalhoi	223/2017	Peter Szebó
Encyclia bocourtii	188b/2013	Peter Szebó
Encyclia bractescens	196/2014	Peter Szebó
Encyclia candollei	229/2014	Peter Szebó
Encyclia ceratistes	ORCH121033	Botanical Garden of the University of Vienna
Encyclia chapadensis	233/2014	Peter Szebó
Encyclia diota	ORCH070265	Botanical Garden of the University of Vienna
Encyclia diurna	39/2009	Peter Szebó
Encyclia fehlingii	155/2013	Peter Szebó
Encyclia fucata	ORCH070257	Botanical Garden of the University of Vienna
Encyclia garciae-esquivelii	ORCH070004	Botanical Garden of the University of Vienna
Encyclia hanburyi	20/2009	Peter Szebó
Encyclia howardii	200b/2014	Peter Szebó
Encyclia huertae	38/2015	Peter Szebó
Encyclia inaguensis	136/2015	Peter Szebó
Encyclia incumbens	94/2015	Peter Szebó
Encyclia ivonae	ORCH070260	Botanical Garden of the University of Vienna
Encyclia meliosma	sn.	Peter Szebó
Encyclia moebusii	ORCH110930	Botanical Garden of the University of Vienna
Encyclia mooreana	22029	Botanical Garden of the University of Vienna
Encyclia naranjapatensis	169/2013	Peter Szebó
Encyclia nematocaulon	06/2005	Peter Szebó
Encyclia odoratissima	ORCH120960	Botanical Garden of the University of Vienna

Encyclia oncidioides	ORCH110535	Botanical Garden of the University of Vienna
Encyclia papillosa	106/2017	Peter Szebó
Encyclia patens	7866171	Botanical Garden of the University of Vienna
Encyclia pauciflora	75/2011	Peter Szebó
Encyclia plicata	158/2013	Peter Szebó
Encyclia powellii	ORCH090178	Botanical Garden of the University of Vienna
Encyclia profusa	113/2012	Peter Szebó
Encyclia rufa	148/2013	Peter Szebó
Encyclia seidelii	ORCH110582	Botanical Garden of the University of Vienna
Encyclia selligera	36/2009	Peter Szebó
Encyclia spiritusanctensis	20506	Botanical Garden of the University of Vienna
Encyclia tampensis	70/2009	Peter Szebó
Encyclia trachychila	198/2015	Peter Szebó

Table S7 Voucher information for the taxa examined in the molecular analysis (fresh material)

Species	Voucher	Source
Encyclia acutifolia	ORCH060621	Botanical Garden of the University of Vienna
Encyclia altissima	22059	Botanical Garden of the University of Vienna
Encyclia amanda	ORCH121035	Botanical Garden of the University of Vienna
Encyclia ambigua	ORCH100320	Botanical Garden of the University of Vienna
Encyclia aspera	ORCH080134	Botanical Garden of the University of Vienna
Encyclia asperula	130453	Botanical Garden of the University of Vienna
Encyclia atropurpurea	sn.	Tadeusz Kusibab
Encyclia bracteata	ORCH060398	Botanical Garden of the University of Vienna
Encyclia ceratistes	ORCH121033	Botanical Garden of the University of Vienna
Encyclia ceratistes	ORCH110591	Botanical Garden of the University of Vienna
Encyclia ceratistis	ORCH070360	Botanical Garden of the University of Vienna
Encyclia cordigera	sn.	Tadeusz Kusibab
Encyclia dichroma	ORCH080286	Botanical Garden of the University of Vienna
Encyclia diota	ORCH070265	Botanical Garden of the University of Vienna
Encyclia diurna	ORCH130565	Botanical Garden of the University of Vienna
Encyclia diurna	sn.	Tadeusz Kusibab
Encyclia fucata	ORCH070257	Botanical Garden of the University of Vienna
Encyclia garcia-esquivelii	ORCH100419	Botanical Garden of the University of Vienna
Encyclia garcia-esquivelii	ORCH090329	Botanical Garden of the University of Vienna
Encyclia garcia-esquivelii	80309	Botanical Garden of the University of Vienna
Encyclia guatemalensis	956	Tadeusz Kusibab
Encyclia guatemalensis	sn.	Tadeusz Kusibab
Encyclia hamburyi	ORCH080302	Botanical Garden of the University of Vienna
Encyclia hanburyi	955	Tadeusz Kusibab
Encyclia hanburyi	sn.	Tadeusz Kusibab
Encyclia ivonae	ORCH070260	Botanical Garden of the University of Vienna
Encyclia microtos	sp.	Botanical Garden of the University of Vienna
Encyclia moebusii	ORCH110930	Botanical Garden of the University of Vienna
Encyclia mooreana	22029	Botanical Garden of the University of Vienna
Encyclia mooreana	ORCH090180	Botanical Garden of the University of Vienna
Encyclia odoratissima	ORCH120960	Botanical Garden of the University of Vienna

Encyclia oncidioides	ORCH110535	Botanical Garden of the University of Vienna
Encyclia oncidioides	sn.	Tadeusz Kusibab
Encyclia osmatha	20507	Botanical Garden of the University of Vienna
Encyclia parviflora	ORCH080305	Botanical Garden of the University of Vienna
Encyclia patens	7866171	Botanical Garden of the University of Vienna
Encyclia plicata	ORCH110941	Botanical Garden of the University of Vienna
Encyclia pollardiana	25711	Botanical Garden of the University of Vienna
Encyclia powellii	ORCH090178	Botanical Garden of the University of Vienna
Encyclia randii	sn.	Tadeusz Kusibab
Encyclia seidelii	ORCH110582	Botanical Garden of the University of Vienna
Encyclia sp.	26327	Botanical Garden of the University of Vienna
Encyclia sp.	ORCH080204	Botanical Garden of the University of Vienna
Encyclia sp.	sn.	Botanical Garden of the University of Vienna
Encyclia spiritusanctensis	20506	Botanical Garden of the University of Vienna
Encyclia virens	ORCH090595	Botanical Garden of the University of Vienna

Table S8 Voucher information for the taxa examined in the multivariate analysis

Species	Voucher	Source
Encyclia acutifolia	33/2016	Peter Szebó
Encyclia altissima	22059	Botanical Garden of the University of Vienna
Encyclia altissima	150/2013	Peter Szebó
Encyclia ambigua	ORCH100320	Botanical Garden of the University of Vienna
Encyclia ambigua	ORCH110653	Botanical Garden of the University of Vienna
Encyclia asperula	144/2013	Peter Szebó
Encyclia belizensis	184/2013	Peter Szebó
Encyclia belizensis	210/2014	Peter Szebó
Encyclia bracteata	ORCH060398	Botanical Garden of the University of Vienna
Encyclia bracteata	262/2017	Peter Szebó
Encyclia candollei	229/2014	Peter Szebó
Encyclia candollei	170/2017	Peter Szebó
Encyclia ceratistes	ORCH110591	Botanical Garden of the University of Vienna
Encyclia ceratistes aff.	ORCH080265	Botanical Garden of the University of Vienna
Encyclia ceratistis	ORCH070360	Botanical Garden of the University of Vienna
Encyclia diota	79/2013	Peter Szebó
Encyclia diurna	39/2009	Peter Szebó
Encyclia diurna	ORCH130565	Botanical Garden of the University of Vienna
Encyclia garciae-esquivelii	ORCH070004	Botanical Garden of the University of Vienna
Encyclia garcia-esquivelii	ORCH100419	Botanical Garden of the University of Vienna
Encyclia garcia-esquivelii	ORCH090329	Botanical Garden of the University of Vienna
Encyclia hamburyi	ORCH080302	Botanical Garden of the University of Vienna
Encyclia hamburyi `dwart`	96/2015	Peter Szebó
Encyclia hanburyi	20/2009	Peter Szebó
Encyclia moebusii	51/2010	Peter Szebó
Encyclia moebusii	162c/2013	Peter Szebó
Encyclia mooreana	ORCH090180	Botanical Garden of the University of Vienna
Encyclia parviflora	ORCH080305	Botanical Garden of the University of Vienna
Encyclia parviflora `dark`	10/2006	Peter Szebó
Encyclia parviflora 'twisted'	13/2007	Peter Szebó
Encyclia patens	128/2012	Peter Szebó

Encyclia patens	ORCH121011	Botanical Garden of the University of Vienna
Encyclia phoenicea	92/2017	Peter Szebó
Encyclia phoenicea	98b/2013	Peter Szebó
Encyclia phoenicea	sn.	Currlin Orchideen
Encyclia plicata	158/2013	Peter Szebó
Encyclia plicata	ORCH110941	Botanical Garden of the University of Vienna
Encyclia plicata	180a/2012	Peter Szebó
Encyclia pyriformis	56/2015	Peter Szebó
Encyclia pyriformis	178/2013	Peter Szebó
Encyclia rufa 'bronze`	148/2013	Peter Szebó
Encyclia rufa 'green'	290/2016	Peter Szebó
Encyclia spiritusanctuensis	43/2009	Peter Szebó
Encyclia tampensis	70/2009	Peter Szebó
Encyclia tampensis	71/2010	Peter Szebó
Encyclia tampensis	114/2014	Peter Szebó
