# Supplementary material for: Characters evolution of Encyclia (Laeliinae-Orchidaceae) reveals a complex pattern not phylogenetically determined: insights from macro- and micromorphology
Source: BMC Plant Biol. 2023 Dec 20;23:661. doi: 10.1186/s12870-023-04664-3 (PMC10731901; doi:10.1186/s12870-023-04664-3)
Supplement: Supplementary file 7 — Additional file 7. Ancestral state reconstruction of micro- and macromorphological features. Table S11. Data matrix for ancestral state reconstruction of micro- and macromorphological traits, where taxa characters were coded for the presence (1 - yes) or absence (0 - no) of a feature. [file 12870_2023_4664_MOESM7_ESM.pdf]

Table S11 Data matrix for ancestral state reconstruction of micro- and macromorphological traits, where taxa characters were coded for the presence (1 - yes) or absence (0 - no) of a feature

Species name	Inflorescence verrucose	Ovary verrucose	Sepals verrucose	Lip middle lobe acute	Lip lateral lobes spread	Callus papillate	Callus sulcate	Sinus shallow	Lip middle lobe flat	Inlorescence dense	Lip papillose	Secrection	Stomata	Trichomes
Encyclia adenocarpa	1	1	0	0	0	0	1	0	1	1	1	1	0	?
Encyclia adenocaula	0	0	0	0	0	0	1	0	1	1	1	1	1	?
Encyclia aenicta	0	0	0	0	0	0	1	0	1	0	1	1	0	?
Encyclia alata	0	0	0	0	0	0	1	0	1	0	0	0	1	?
Encyclia amanda	0	0	0	0	0	0	1	0	1	0	0	1	0	?
Encyclia ambigua	0	0	0	0	0	0	1	0	1	0	1	1	0	0
Encyclia andrichii	1	0	0	0	0	0	1	0	1	0	1	1	0	?
Encyclia aspera	1	1	0	0	0	0	0	0	1	0	0	0	0	?
Encyclia asperula	1	1	0	0	1	0	1	0	1	0	0	0	0	?
Encyclia belizensis	0	0	0	0	0	0	1	0	1	0	1	1	1	?
Encyclia bocourtii	0	0	0	0	0	0	1	0	0	0	0	1	0	?
Encyclia bractescens	0	0	0	0	0	0	1	0	1	0	1	0	0	0
Encyclia candollei	0	0	0	1	1	0	1	0	1	0	0	0	0	?
Encyclia ceratistes	1	0	0	0	0	0	1	0	1	0	0	1	0	?
Encyclia chapadensis	0	0	0	0	0	0	0	0	1	0	0	0	0	?
Encyclia cordigera	0	0	0	0	0	0	1	0	1	1	1	1	0	?
Encyclia dichroma	0	0	0	0	0	0	1	0	0	1	1	0	0	?
Encyclia diota	0	0	0	0	0	0	1	0	0	0	1	1	0	?
Encyclia diurna	1	0	0	0	0	0	1	0	0	0	0	0	1	?
Encyclia fucata	1	0	0	0	0	0	1	0	1	1	1	1	0	?
Encyclia garciae-esquivelii	1	1	0	0	0	0	1	0	1	0	0	0	0	?
Encyclia guatemalensis	1	1	0	0	1	0	1	0	1	0	1	0	0	?
Encyclia hanburyi	0	0	0	0	0	0	1	0	1	1	1	1	0	0
Encyclia huertae	0	0	0	0	0	0	1	1	1	0	0	1	0	?
Encyclia inaguensis	0	0	0	0	0	0	1	0	1	0	0	1	1	?
Encyclia incumbens	0	0	0	0	0	0	1	0	1	0	1	0	0	0
Encyclia ivonae	0	0	0	0	1	0	0	0	1	0	1	1	0	?
Encyclia meliosma	0	0	0	0	0	0	1	1	1	0	1	1	0	?
Encyclia microtos	1	0	0	0	0	0	1	0	1	0	0	1	1	0
Encyclia mooreana	1	0	0	0	0	0	1	0	0	0	0	1	0	?
Encyclia naranjapatensis	0	0	0	0	0	0	0	0	1	0	1	1	0	?
Encyclia nematocaulon	0	1	0	1	0	0	0	0	1	0	1	1	0	0
Encyclia oncidioides	0	0	0	0	0	0	0	0	1	1	0	1	0	?
Encyclia osmantha	0	0	0	0	0	0	0	0	1	0	0	1	1	?
Encyclia parviflora	1	0	0	0	0	0	1	0	1	0	0	1	1	?
Encyclia patens	0	0	0	1	0	0	1	0	1	0	0	0	0	?

Encyclia pauciflora	0	0	0	0	0	0	1	0	1	0	0	0	0	?
Encyclia plicata	0	0	0	0	0	0	1	0	0	0	0	1	0	?
Encyclia pollardiana	0	0	0	0	1	0	1	0	0	0	0	1	1	1
Encyclia profusa	0	0	0	0	0	0	1	0	1	0	0	1	0	?
Encyclia rufa	0	0	0	0	0	0	1	0	0	0	0	0	1	?
Encyclia seidelii	0	0	0	0	1	0	1	1	1	0	0	0	0	?
Encyclia selligera	0	0	0	0	0	0	1	0	1	0	0	1	1	?
Encyclia spiritusanctensis	0	0	0	0	0	0	1	0	1	0	1	1	0	?
Encyclia tampensis	0	0	0	0	0	0	1	0	1	0	0	0	0	?
Encyclia trachycarpa	1	1	0	0	0	0	1	1	1	0	1	1	0	?
Encyclia trachychila	1	1	0	0	0	0	1	1	1	1	1	0	0	?
